# Tissue- and time-dependent metabolite profiles during early grain development under normal and high night-time temperature conditions

**DOI:** 10.1186/s12870-024-05190-6

**Published:** 2024-06-18

**Authors:** Nathan Abshire, Andrew L. Hauck, Harkamal Walia, Toshihiro Obata

**Affiliations:** 1 Department of Biochemistry, University of Nebraska-Lincoln, Lincoln, NE USA; 2 Center for Plant Science Innovation, University of Nebraska-Lincoln, Lincoln, NE USA; 3 Department of Agronomy and Horticulture, University of Nebraska-Lincoln, Lincoln, NE USA; 4 Department of Biochemistry, Center for Plant Science Innovation, University of Nebraska-Lincoln, 1901 Vine Street, Lincoln, Nebraska, 68588 USA

**Keywords:** Wheat, Early grain development, Day/night oscillation, Source-sink metabolism, Bract, Flag leaf, High night temperature

## Abstract

**Background:**

Wheat grain development in the first few days after pollination determines the number of endosperm cells that influence grain yield potential and is susceptible to various environmental conditions, including high night temperatures (HNTs). Flag leaves and seed-associated bracts (glumes, awn, palea, and lemma) provide nutrients to the developing seed. However, the specific metabolic roles of these tissues are uncertain, especially their dynamics at different developmental stages and the time in a day. Tissue- and time-dependent metabolite profiling may hint at the metabolic roles of tissues and the mechanisms of how HNTs affect daytime metabolic status in early grain development.

**Results:**

The metabolite profiles of flag leaf, bract, seed (embryo and endosperm), and entire spike were analyzed at 12:00 (day) and 23:00 (night) on 2, 4, and 6 days after fertilization under control and HNT conditions. The metabolite levels in flag leaves and bracts showed day/night oscillations, while their behaviors were distinct between the tissues. Some metabolites, such as sucrose, cellobiose, and succinic acid, showed contrasting oscillations in the two photosynthetic tissues. In contrast, seed metabolite levels differed due to the days after fertilization rather than the time in a day. The seed metabolite profile altered earlier in the HNT than in the control condition, likely associated with accelerated grain development caused by HNT. HNT also disrupted the day/night oscillation of sugar accumulation in flag leaves and bracts.

**Conclusions:**

These results highlight distinct metabolic roles of flag leaves and bracts during wheat early seed development. The seed metabolite levels are related to the developmental stages. The early metabolic events in the seeds and the disruption of the day/night metabolic cycle in photosynthetic tissues may partly explain the adverse effects of HNT on grain yield.

**Supplementary Information:**

The online version contains supplementary material available at 10.1186/s12870-024-05190-6.

## Background

An abundant and high-quality supply of wheat is needed, as wheat accounts for 20% of calories consumed by humans [1]. The quantity and quality of wheat seeds are significantly affected during wheat grain development after anthesis. Wheat grain development can be divided into three phases: Early grain development (0–14 days after fertilization; DAF), grain filling (14–28 DAF), and maturation and desiccation (28 DAF-maturity) [2, 3]. During the early grain development phase, the seed undergoes rapid mitotic division and uptake of water in order to grow and differentiate the various parts of the seed, such as the endosperm, embryo, and aleurone [3]. The first six days of early grain development are especially crucial to forming endosperm. Immediately after fertilization to 2 DAF is the coenocytic stage, where free endosperm nuclei undergo division without cytokinesis and line the central endosperm vacuole. In the cellularization stage, which occurs from 2 DAF until 6 DAF, the free nuclei develop alveolar cell walls which are open towards the central vacuole. This layer of cells then divides until the central vacuole is filled with endosperm cells. In the third stage, starting at 6 DAF, rapid mitotic cell division of endosperm cells and the accumulation of starch and storage proteins begin to occur [4]. These early developmental events, especially cellularization, determines the number and size of endosperm cells which limits the space for storage molecule accumulation in subsequent stages. During the second phase of grain development, grain filling, it was previously found that seed physiological, morphological, and transcriptional changes were mirrored by dynamic changes in metabolite profiles [4]. Therefore, we assume that the events of early grain development can also be seen in the metabolite profile. The developing seed is heterotrophic and depends on source tissues for the delivery of metabolic substrates for growth and development. The flag leaves as well as the seed-associated bracts (palea, lemma, awn, and glumes) are important source tissues for supplying the developing seed with nutrients [5]. Previous research has suggested that each of these two sources has unique roles [6, 7], and metabolite profiles [8]. The flag leaf supplies the seed but also other heterotrophic parts of the plant, such as the roots. The flag leaf also exports nutrients to the entire plant at night [9]. The bracts may play a more specialized role as a local source to the proximal seed [10]. The bracts also have an important role in recycling carbon dioxide released by the seed’s respiration [11, 12]. In sorghum and wild grasses, spikelets and associated bracts not only act as direct sources for the seed but also as intermediate sinks. The seed-associated bracts collect metabolic substrates produced by sterile spikelets for distribution to the seed and its own metabolic requirements [13]. Besides photosynthates, bracts may also act as an intermediate sink for nitrogen containing metabolites, as 42% of grain nitrogen content comes from metabolites translocated from the bract in durum wheat [14].

Day and night cycles are a very important aspect of the source-sink relationships that occur throughout a plant’s life cycle. During the day, photosynthetic tissues are autotrophic, with much ATP and reducing power being derived from photosynthesis. A surplus of fixed carbon during the day allows the accumulation of storage carbon, such as starch. Some plant tissues are heterotrophic sinks and depend on photosynthetic source tissues for their metabolic needs [15, 16]. Also, the entire plant becomes heterotrophic during the night and depends on the photosynthetic surplus accumulated during the day. Despite the importance of the diurnal aspect of plant metabolism, very few studies of metabolism in developing seeds have taken day and night differences into account. One study on tomato showed that many metabolites exhibit day/night oscillations in the accumulations in both leaf source tissues and the developing fruit sink tissues [17]. Day and night differences have also been observed during wheat seed development at the physiological level, where it was shown that mitotic cell division occurred primarily during the night [18]. However, studies investigating the day/night oscillation of metabolite profiles during early grain development are scarce.

Early grain development is also a time when crops are especially sensitive to stress, such as high night temperatures (HNTs). HNT is a growing concern in agriculture affecting wheat yield [19, 20]. Also, night-time temperatures are recently increasing more quickly than daytime temperatures [21, 22]. Night-time heat stress has also been shown to have different effects from daytime heat stress [23]. It has been previously shown that HNT stress accelerates wheat seed development, causing fewer days until physiological maturity [24]. In rice, this accelerated maturation by HNT during early grain development leads to incomplete cellularization and lower grain quality [25]. Metabolic profiling has revealed that HNT alters the metabolism of sugars, amino acids, and phenolics during early grain development in wheat [24].

The metabolic aspect of source-sink relationships changes throughout the day/night cycle, yet there has not been a metabolic profiling study that has taken day/night time measurements as well as inter-day measurements to characterize early seed development. Day and night metabolism are different, and the two time periods affect each other. Therefore, sampling time points during both day and night should be used for a complete understanding of early grain development. Although the HNT effects on grain development are different from those of daytime heat stress, metabolic profiling has never been reported at both day and night time points in the same study. It has also been demonstrated in rice that when HNT stress is applied, metabolism is affected during the daytime as well, a time when no stress is being applied [26]. Here, we aimed to characterize how HNT changes the way that day and night metabolisms transit and interact. By addressing these knowledge gaps, we found that developmental events are associated with the metabolite profile, and flag leaves and bracts have distinct roles during early wheat grain development. HNT accelerated the metabolic time courses in seeds and perturbated day and night oscillations in the flag leaves and bracts, which may partly contribute to the grain yield penalty by HNT.

## Results

### Metabolites accumulated differentially across four tissues during the early grain development, with many bract and flag leaf metabolites showing day/night oscillation

We examined the day and night metabolite accumulation during early seed development in wheat. We sampled four wheat tissues (flag leaf, bract, seed, and spike) to characterize source-sink relationships during early seed development. We selected three sampling days to represent two development transitions. 2 DAF represents the end of the coenocytic division of endosperm nuclei and the transition into cellularization. 4 DAF represents the peak of cellularization. 6 DAF represents the transition into early grain filling. We sampled at two time points on each of these days (12:00 and 23:00) in order to investigate the metabolite profiles during the day and night, respectively. We analyzed the accumulation time course of 48 metabolites commonly detected among all tested tissues (Supplemental Data 1). Principal component analysis (PCA) indicated distinctive tissue metabolite profiles (Supplemental Fig. S1). The spike metabolite profile was similar to bract, likely because bract is the major tissue in the spike in this developmental stage (Supplemental Fig. S1). Global seed metabolome altered between DAF rather than the time in a day. Especially, the metabolite profile of 2 DAF seed are clearly separated from 4 to 6 DAF seeds in PCA. The differences of global metabolite profiles depending either on DAF and the time in the day were not apparent in other tissues (Supplemental Fig. S1).

**Fig. 1:**
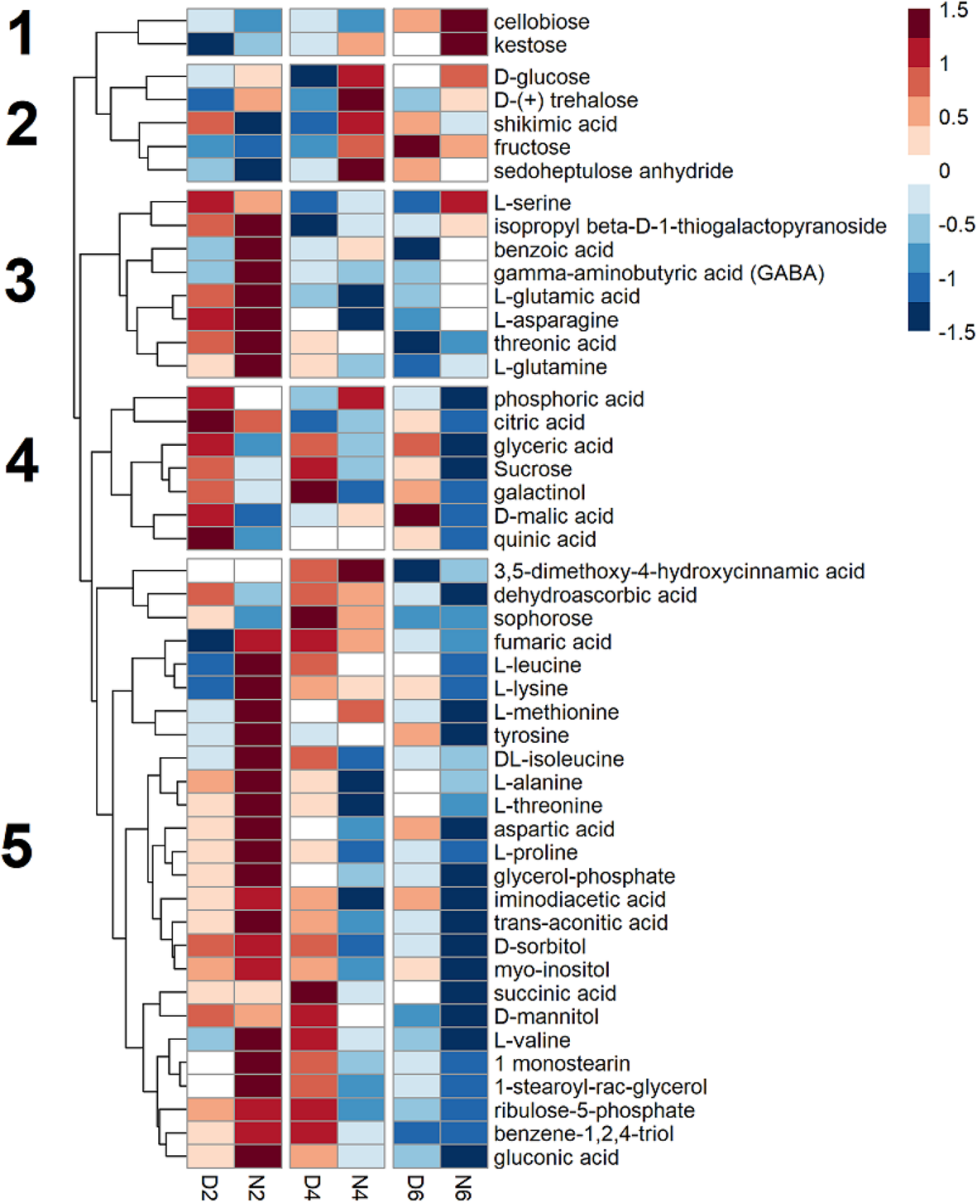
Time courses of metabolite abundance in flag leaves during early seed development. Each row represents a metabolite. Columns represent time points, where D or N represent day or night time sampling and 2, 4, and 6 represent the days after fertilization (DAF) at sampling. Each square is a mean of up to five biological replicates of relative metabolic abundances. Values in each row (metabolite) were log_2_ transformed, and Z transformed with a mean of zero and a standard deviation of 1. A color scale of red to blue represents metabolite abundances above and below the row mean, respectively. Numbers to the left of the heatmap identify hierarchical clusters

Hierarchical cluster analysis was performed to group metabolites in individual tissues showing similar accumulation time courses. In the flag leaves, the metabolites were clustered into five groups (Fig. 1). Metabolites in Cluster 4 tended to accumulate during the day and decrease during the night, including citric acid, sucrose, glyceric acid, and malic acid. Metabolites in Cluster 5, including leucine, methionine, isoleucine, and aspartic acid, also showed higher accumulation during the day after 4 DAF, but they highly accumulated during the night at 2 DAF and decreased afterward. Cluster 3 includes the metabolites, such as benzoic acid, GABA, glutamic acid, and threonic acid, that tended to accumulate during the night and showed decreasing trend following 4 DAF. Metabolites in Clusters 1 (cellobiose and kestose) and 2 (glucose, trehalose, shikimic acid, fructose, and sedoheptulose anhydride) accumulated in the nights of 6 DAF and 4 DAF, respectively.

**Fig. 2:**
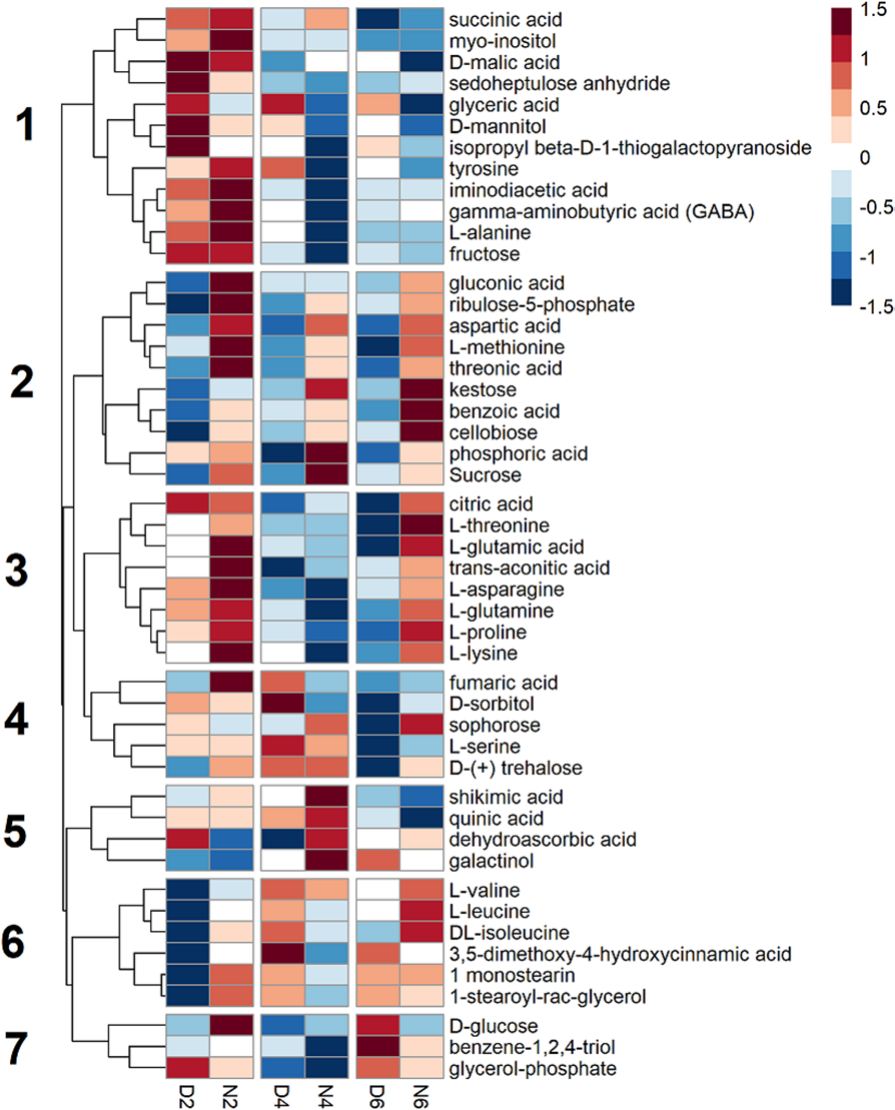
Time courses of metabolite abundance in bracts during early seed development. Each row represents a metabolite. Columns represent time points, where D or N represent day or night time sampling and 2, 4, and 6 represent the days after fertilization (DAF) at sampling. Each square is a mean of up to five biological replicates of relative metabolic abundances. Values in each row (metabolite) were log_2_ transformed, and Z transformed with a mean of zero and a standard deviation of 1. A color scale of red to blue represents metabolite abundances above and below the row mean, respectively. Numbers to the left of the heatmap identify hierarchical clusters

Bracts metabolites were clustered into seven groups depending on their accumulation time courses (Fig. 2). Metabolites in Cluster 2, such as gluconic acid, ribulose-5-phosphate, aspartic acid, methionine, threonic acid, kestose, benzoic acid, and sucrose, accumulated during the night. Cluster 3 metabolites, including citric acid, threonine, glutamic acid, trans-aconitic acid, glutamine, and proline, also accumulated during the night, except for 4 DAF. Cluster 6 includes branched chain amino acids that showed similar night-time accumulation as Cluster 3 metabolites but showed increasing tendency over the experiment. Cluster 1 includes metabolites that tended to accumulate during the day but decreased through the experimental period, such as succinic acid, malic acid, tyrosine, GABA, alanine, and fructose. Metabolites in Cluster 5 (shikimic acid, quinic acid, dehydroascorbic acid, and galactinol) and 7 (glucose, benzene-1,2,4-triol, and glycerol-phosphate) accumulated specifically at 4 DAF night and 6 DAF day, respectively.

**Fig. 3:**
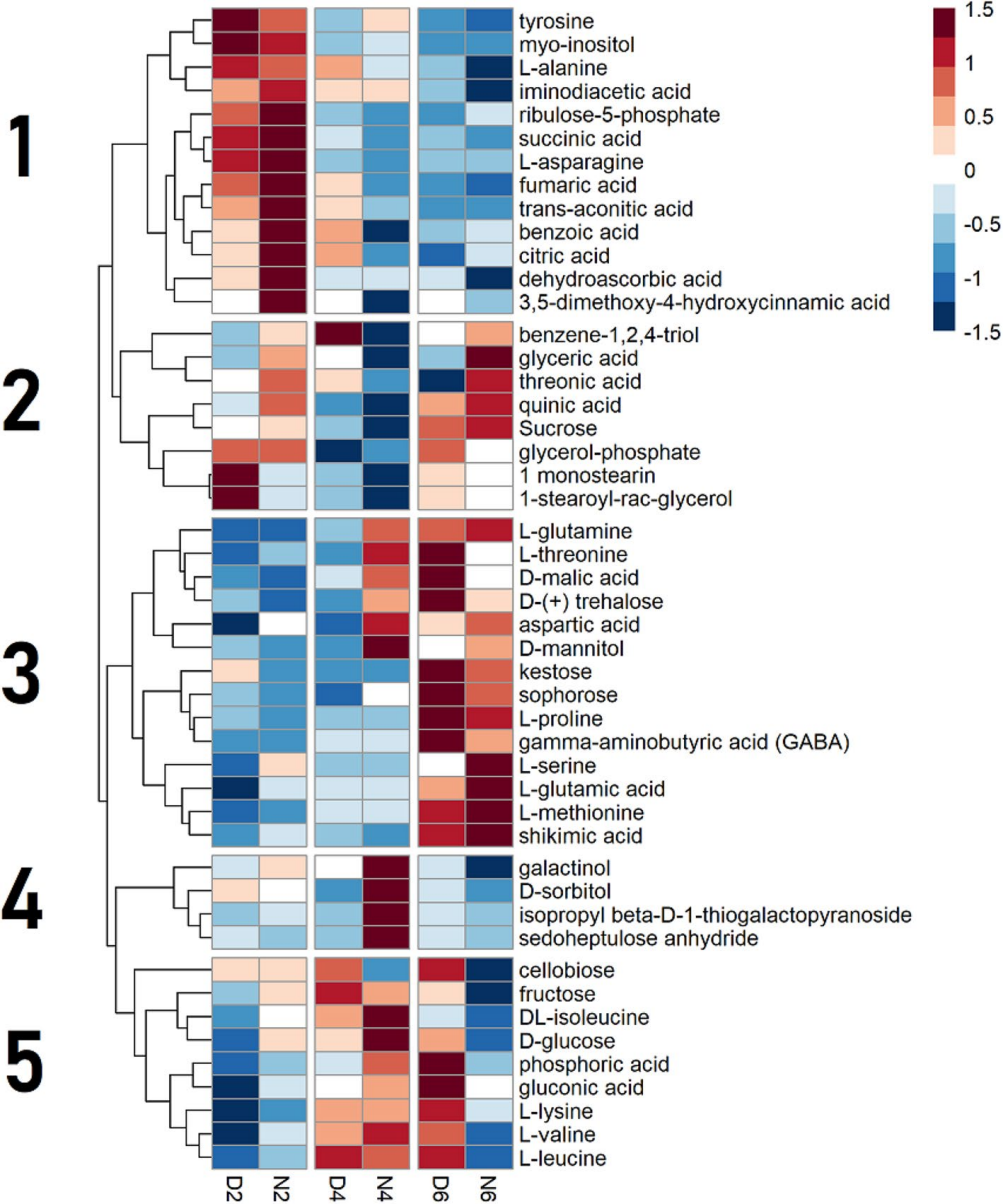
Time courses of metabolite abundance in seeds during early seed development. Each row represents a metabolite. Columns represent time points, where D or N represent day or night time sampling and 2, 4, and 6 represent the days after fertilization (DAF) of sampling. Each square is a mean of up to five biological replicates of relative metabolic abundances. Values in each row (metabolite) were log_2_ transformed, and Z transformed with a mean of zero and a standard deviation of 1. A color scale of red to blue represents metabolite abundances above and below the row mean, respectively. Numbers to the left of the heatmap identify hierarchical clusters

The metabolites determined in the seed were clustered into five groups, mainly depending on the DAF (Fig. 3). Cluster 1 includes metabolites that decreased at daytime 4 DAF and remained low accumulation. This cluster contains tyrosine, alanine, asparagine, succinic acid, fumaric acid, trans-aconitic acid. Cluster 2 described a time course where metabolites fell at 4 DAF and re-accumulated at 6 DAF. This cluster contained sucrose, threonic acid, and quinic acid. Cluster 3 metabolites accumulated at 6 DAF, including malic acid, GABA, glutamate, glutamine, threonine, serine, methionine, shikimate, and proline. Cluster 4 metabolites, galactinol, sorbitol, isopropyl beta-D-1-thiogalactopyranoside, and sedoheptulose anhydride, accumulated during the night time of 4 DAF. Cluster 5 described metabolites that accumulated at 4 DAF and decreased during the night of 6 DAF. This cluster contained glucose, fructose, cellobiose, phosphoric acid, lysine, valine, leucine, and isoleucine.

**Fig. 4:**
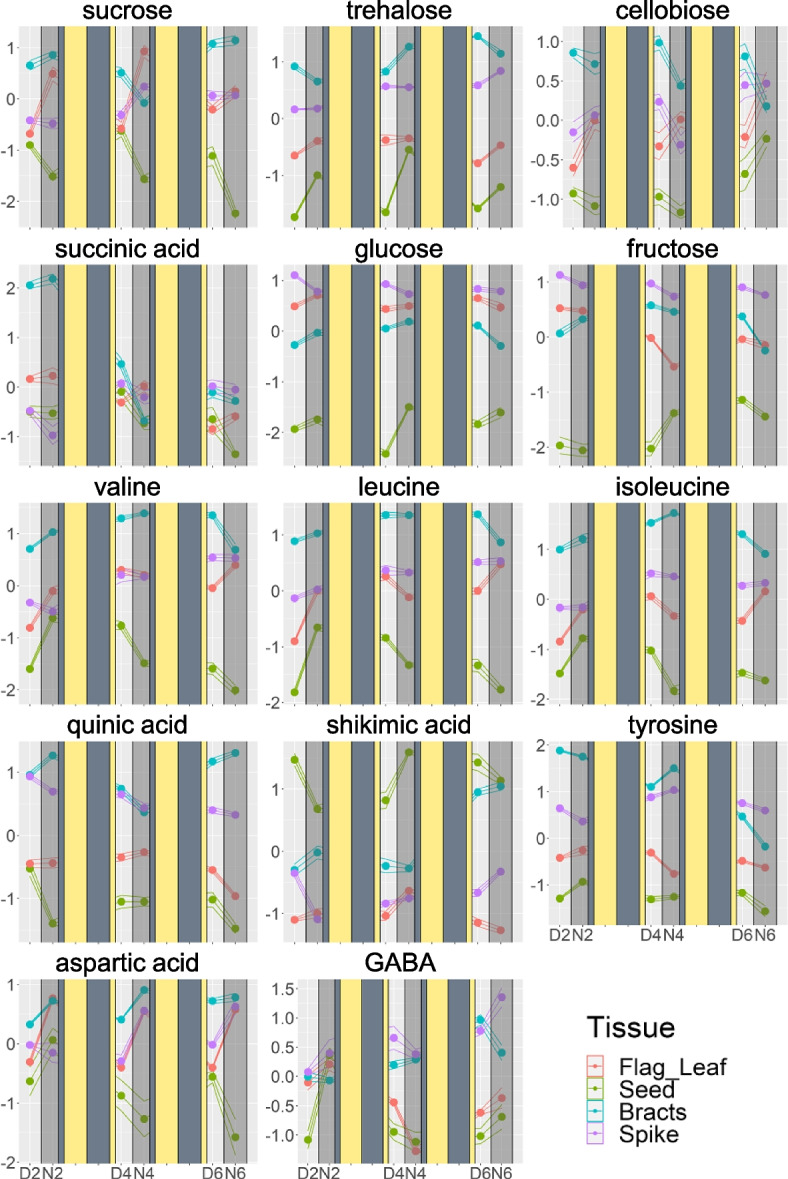
Time courses of the abundance of selected metabolites. Y axis represents relative metabolic abundance. X axis represents time point, where D or N represent day or night time sampling and 2, 4, and 6 represent the days after fertilization (DAF) of sampling. Solid rectangles were overlaid to illustrate the days between sample collection, with dull yellow representing day, and dull blue representing night. Data are presented as mean values at each time point and flanking lines representing one +- standard error, from up to five biological replicates. Color represents tissue type. All values for each metabolite, including all tissue types, were log_2_ transformed, and Z transformed with a mean of zero and a standard deviation of 1

The metabolites measured in the spike were grouped into four clusters (Supplemental Fig. S2), which also tend to be related to the accumulation at specific DAF. Malic acid, serine, 1-monosterin, and 1-stearoyl-*rac*-glycerol in Cluster 1 accumulated 2 DAF night. Phosphoric acid, myo-inositol, tyrosine, and galactinol accumulated 4 DAF night in Cluster 3. Most metabolites, including sucrose, valine, leucine, isoleucine, shikimic acid, benzoic acid, kestose, glutamic acid, GABA, methionine, glutamine, and threonine, were clustered in Cluster 2 that shows significant accumulation at 6 DAF night. Only the Cluster 4 metabolites accumulated during the day especially at 2 DAF. This cluster contained glyceric acid, sedoheptulose, ribulose-5-phosphate, glucose, fructose, citric acid, and asparagine. The line plots of the levels of individual metabolites are in Fig. 4 and Supplemental Fig. S3.

**Fig. 5:**
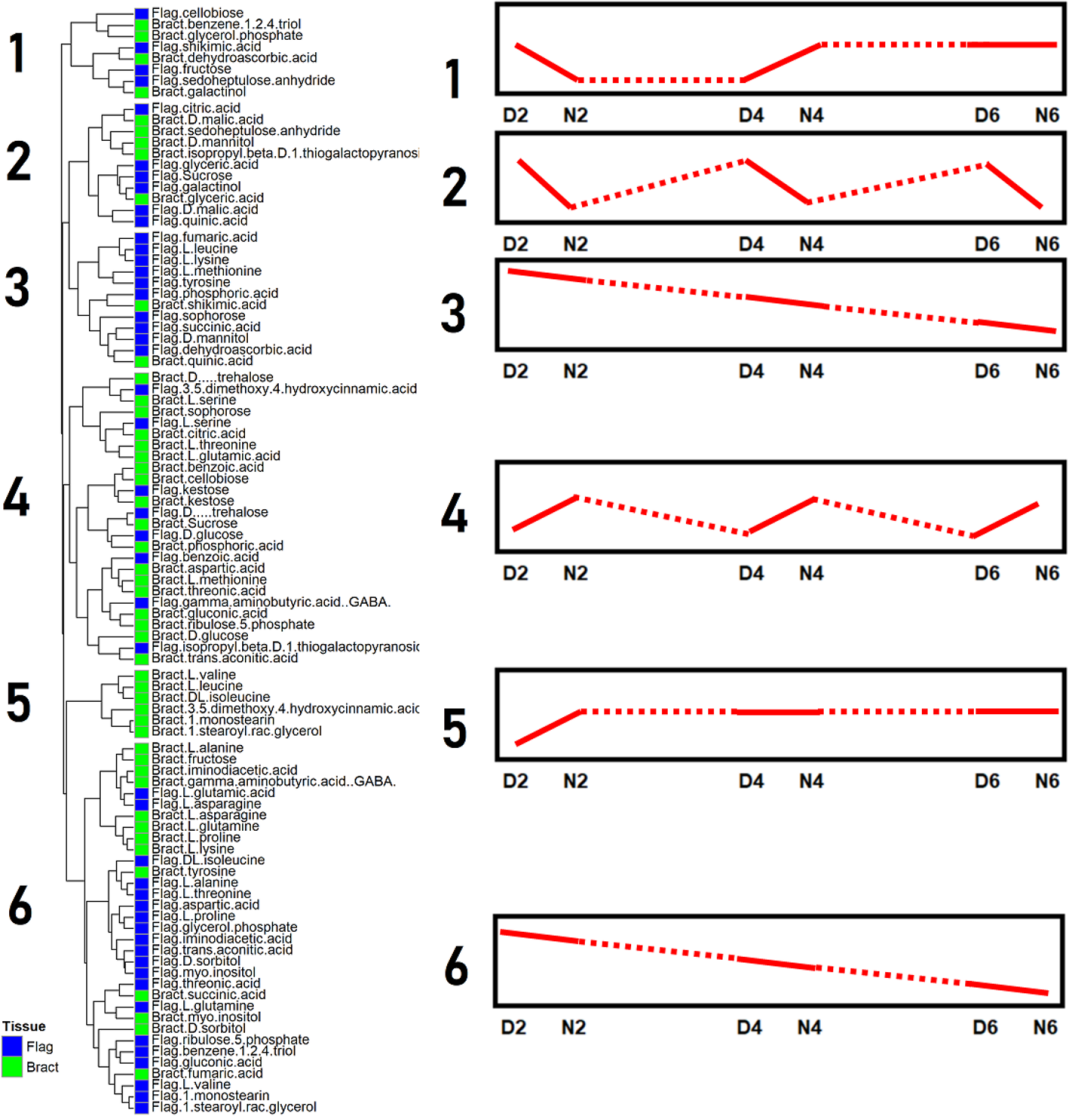
Hierarchical clustering of metabolites of flag leaves and bracts during early seed development. Each row represents a metabolite, and the first column represents the tissue from where the metabolite was measured. Numbers to the left of the heatmap identify hierarchical clusters. Sketches to the right of each cluster represent time courses in each cluster, the x axis of the sketches shows time points, D or N represent day or night time sampling, and 2, 4, and 6 represent the days after fertilization (DAF) of sampling

We compared the metabolite accumulation in the flag leaves and bracts by clustering all metabolites from these tissues together. When a metabolite in both flag leaves and bracts are in the same cluster, it shows similar accumulation time courses in both tissues. Metabolites were grouped into six clusters, each characterized by a distinct time course accumulation (Fig. 5). Almost all metabolites accumulated differently in the two tissues, with only a few metabolites showing concerted time courses (Table 1). Metabolites accumulated during the day were clustered in Cluster 2, which includes malic acid and glyceric acid common in both tissues. Cluster 4 metabolites accumulated during the night, and a majority of bract metabolites were in this cluster, such as sucrose, cellobiose, threonine, glutamic acid, aspartic acid, methionine, ribulose-5-phosphate, and gluconic acid. Trehalose, kestose, glucose, benzoic acid, and serine in both tissues were included in this cluster. Metabolites in Cluster 3 and 6 tended to decrease over the experimental period, and a majority of the metabolites measured in flag leaves, including valine, leucine, and isoleucine, tyrosine, aspartic acid, glutamic acid, ribulose-5-phosphate, and gluconic acid, were in these clusters. Fumaric acid, succinic acid, alanine, asparagine, glutamine, and proline were in these clusters in both flag leaves and bracts. Clusters 1 and 5 include no metabolites common in both flag leaves and bracts.

**Table 1:** Metabolites in Flag leaf and bract which have similar time courses

Cluster	Time course	Shared metabolites
1	Decrease until N2, increase N4 or D6	None
2	Oscillation peaking during day	Malic acid and glyceric acid
3 & 6	Increase until N2, decreasing subsequently	Fumaric acid, succinic acid, alanine, asparagine, glutamine, and proline
4	Oscillation peaking during night	Trehalose, kestose, and glucose, benzoic acid and serine
5	Increase until N2, remains high	None

The similarity of time courses was based on the hierarchical clustering depicted in Fig. 4

### HNT caused an acceleration of metabolic time courses in seed and perturbation of day/night oscillation in source tissues

**Fig. 6:**
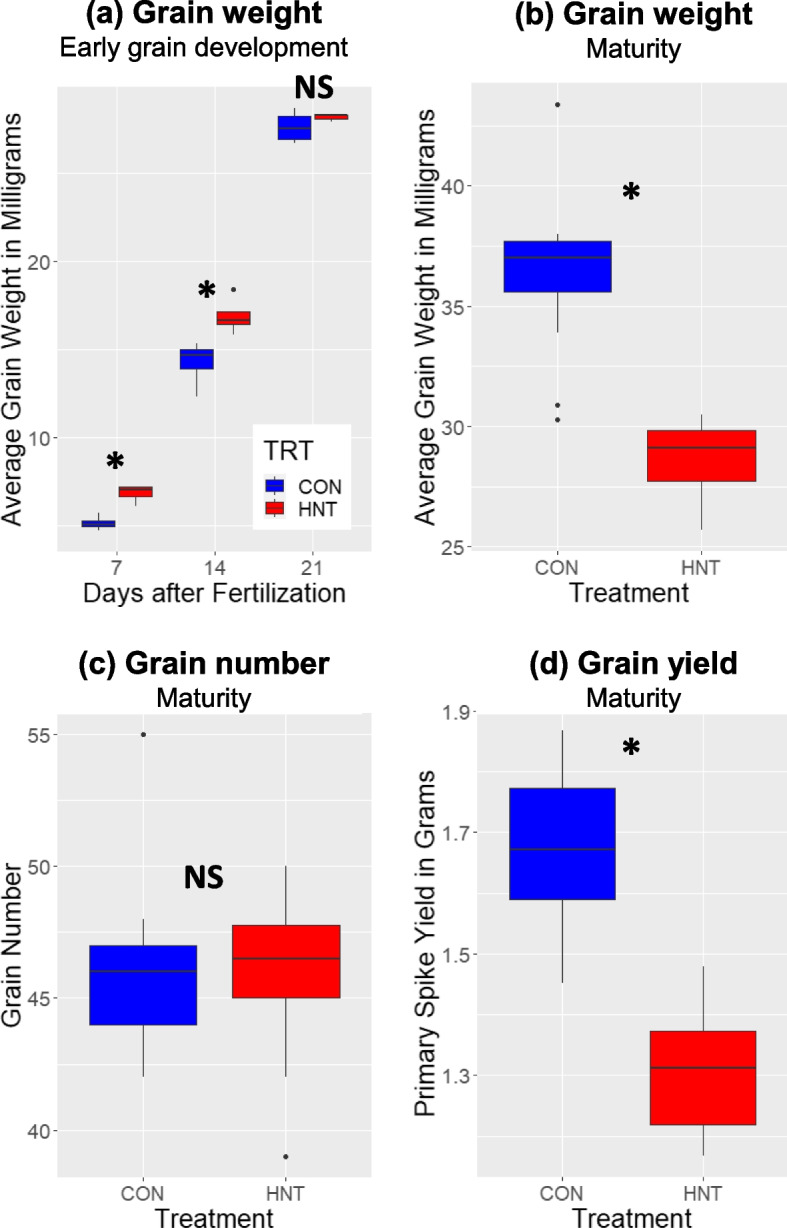
Effect of high night temperature (HNT) on wheat grain development and yield. Blue and red represent the plants grown in the control and HNT conditions, respectively. Asterisks indicate the statistically significant differences between control and HNT conditions tested by *t*-test (*p* < 0.05). NS indicates no significant difference. **a** Average grain weight (mg) during early grain development at 7, 14, and 21 days after fertilization (DAF). The values represent the average weight of five wheat grains sampled from the primary spike (*n* = 4). **b** Average grain weight at maturity. The value shows the average weight of individual grains from the primary spike in milligrams (*n* = 10). **c** Grain number on the primary spikes at maturity. The value shows the count of grains on the primary spike (*n* = 10). **d** Total grain yield on the primary spike at maturity. The value shows the total dry weight of seeds from the primary spike in grams (*n* = 10)

The grain weight was significantly higher under the HNT condition than the control condition during the early grain development until 14 DAF (Fig. 6a). However, the grain weights under these conditions are comparable at 21 DAF (Fig. 6a), and HNT plants showed lower grain weights at maturity (Fig. 6b). There were no significant differences in the number of grains under control and HNT conditions (Fig. 6c). The total grain yield of the primary spike is 22% lower in the HNT condition (Fig. 6d), which is attributed to the lower weight of individual grains (Fig. 6b). Additionally, we visually observed early leaf yellowing and leaf yellow spots under HNT condition, although no quantitative image is available. These results indicate that HNT accelerated the grain filling rate during early grain development but reduced the grain yield at maturity.

**Fig. 7:**
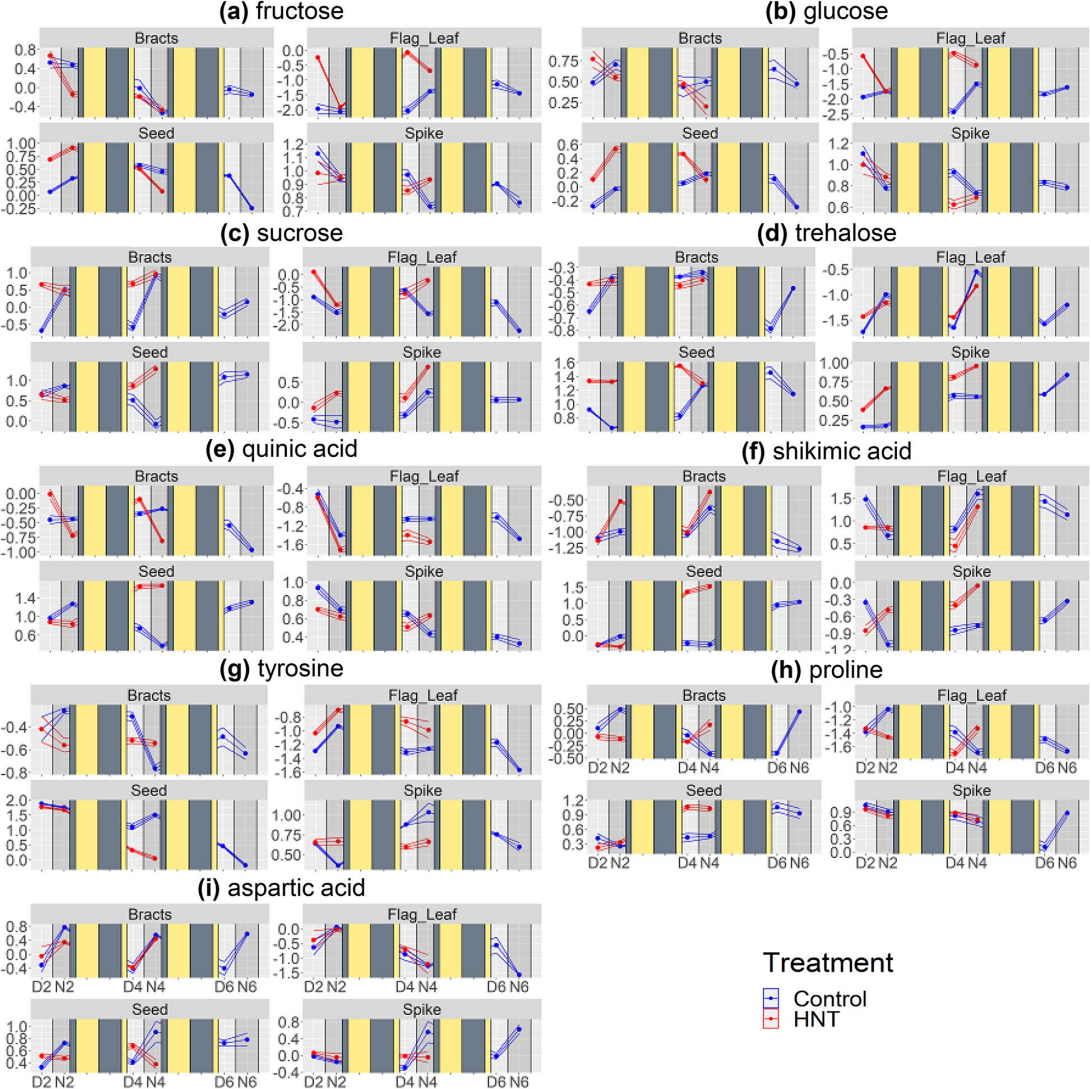
Time courses of the abundance of selected metabolites in the wheat plants treated by high night temperature (HNT). The Y axis represents relative metabolic abundance. All values for each metabolite under all conditions, including all treatments and tissue types, were log_2_ transformed, and Z transformed with a mean of zero and a standard deviation of 1. X axis represents time point, where D or N represent day or night time sampling and 2, 4, and 6 represent the days after fertilization (DAF) of sampling. Solid rectangles were overlaid over the chart to represent the days between sample collection with dull yellow representing day, and dull blue representing night. Data is represented as mean values at each time point, as well as flanking lines representing one +- standard error, from up to five biological replicates. Each facet represents a tissue. Color within the facet represents treatment. HNT, high night temperature. **a** Fructose was affected by temperature in the flag leaf and seed, and to temperature x DAF in seed. **b** Glucose was affected by temperature and temperature x day/night in the flag leaf. **c** Sucrose was affected by temperature in the bract. **d** Trehalose was affected by temperature in seed and spike, and by temperature x day/night in the flag leaf. **e** Quinic acid was affected by temperature x day/night in bract and temperature x DAF in seed. **f** Shikimic acid was affected by temperature x DAF in seed. **g** Tyrosine was affected by temperature x DAF in seed; (**h**) Proline was affected by temperature x DAF in seed. **i** Aspartic acid was affected by temperature x day/night in the seed

Metabolite profiling analysis was also carried out on flag leaves, bracts, seed, and spike tissues subjected to HNT following anthesis. ANOVA identified 13 metabolites whose abundance was significantly affected by HNT treatment (temperature) and combined effects of HNT with the day/night (temperature x day/night), and DAF (temperature x DAF) at least in one tissue. We analyzed the time course of the abundance of these 13 metabolites to elucidate HNT effects on metabolism. All 13 metabolites showed shortened time courses under HNT compared to the control condition in the seeds (Fig. 7, Supplemental Fig. S4). Major sugars, namely fructose, glucose, and sucrose (Fig. 7a-c), and minor sugars, trehalose (Fig. 7d) and kestose (Supplemental Fig. S3), decreased in the seeds on 4 DAF under HNT in contrast to 6 DAF under control condition. Similarly, the increase of shikimate pathway related compounds, quinic acid and shikimic acid (Fig. 7e, f), and organic acids, malic acid, and trans-aconitic acid (Supplemental Fig. S3) happened on 4 DAF in HNT earlier than 6 DAF in control. Changes in amino acid levels also occurred two days earlier under HNT than control (Fig. 7g-i). Interestingly, the day/night oscillation of abundance was perturbed in several metabolites in photosynthetic tissues. The fructose and glucose levels were high during the day and low during the night in HNT-treated flag leaves, while they tended to be higher during the night under the control condition (Fig. 7a, b). Sucrose and trehalose levels in bracts were higher during the night under the control condition but did not oscillate under HNT (Fig. 7c, d). Contrarily, the levels of quinic acid and shikimic acid in bracts showed day/night oscillation, specifically under HNT (Fig. 7e, f). Gluconic acid accumulated in bracts and flag leaves at 2 DAF night in the control condition, but their levels did not increase at that time under HNT (Supplementary Fig. S4).

## Discussion

The current results highlighted the different metabolic roles of flag leaves and bracts in early grain development. The day/night oscillations of many metabolites showed contrasts between the two source tissues (Fig. 5). We found that most day/night oscillation was seen in the flag leaves and the bracts, spanning several metabolic classes such as sugars, amino acids, and organic acids. Unlike in source tissues, very few metabolites in the seed have clear day/night oscillation. However, the seed metabolites that showed weak day and night oscillations were mainly translocated amino acids, such as aspartic acid and methionine, which accumulated to greater amounts throughout the time course (Fig. 3). Metabolites in the bracts tissue showed the tendency of day/night oscillation where metabolic abundances peak during the night and deplete during the day (Fig. 2). The metabolite accumulation during the night suggests that metabolites are being translocated to the bracts from other source tissues during the night to act as a reserve for the developing seed, although the night time accumulation may be due to the metabolic activities in the bract. The depletion of these metabolites during the day may be due to the consumption by the developing seed. Besides translocated metabolites, the citric acid cycle intermediates, succinic acid and citric acid, and the pentose phosphate pathway intermediates, gluconic acid and ribulose-5-phosphate, also peaked during the night in the bracts (Fig. 2), suggesting greater respiration and the pentose phosphate pathway fluxes during the night. Surprisingly, only three metabolites (benzoic acid, glyceric acid, threonic acid, kestose) oscillated in the same direction in both flag leaves and bracts. Even more interesting, sucrose and succinic acid exhibited inverse oscillations in flag leaves and bracts, with levels peaking during the night in bracts and during the day in flag leaves (Figs. 4 and 5). This contrast also highlights major day/night differences of the metabolism in the two source tissues. However, it should be noted that bract also plays a source role by photosynthetically assimilating CO_2_ from the atmosphere and endosperm respiration [27, 28]. Bract photosynthesis is estimated to contribute 70% of sucrose accumulation in bracts [29] and plays a significant role as source tissue, especially under suboptimal conditions [30, 31]. Therefore, the day/night oscillations of metabolite accumulation in bract probably result from the balance between its sink and source roles, which might be perturbed under unfavorable conditions such as HNT (Fig. 7).

The results in this study suggested that key developmental events are associated with metabolite levels in the analyzed tissues. The timing of the events of wheat grain development has been shown to occur at timepoints that were consistent between studies [2, 3]. It has also been shown that these developmental events are associated with metabolic alterations [32, 33]. Time courses of metabolite levels seen in all four tissues appeared to reflect two events in early grain development: cellularization and the beginning of grain filling. The hierarchical clustering analysis results of the seed clearly showed the alterations in metabolite accumulation at these developmental stages (Fig. 3), with clusters characterized by significant metabolic changes at cellularization (4 DAF) or the beginning of grain filling (6 DAF). Previous studies indicated that the green seed pericarp photosynthetically fixes CO_2_ evolved from seed respiration but fixes a limited amount of atmospheric CO_2_ due to the little gas exchange on the seed outer surface [6, 30]. Therefore, pericarp photosynthesis is likely affected by respiratory metabolism rather than light availability, leading to little day/night difference in seed metabolite accumulation. The metabolic changes in spikes were also related to the developmental stages (Fig. 4). Although the clusters seen in the spike appeared similar to those seen in the seed, the individual metabolites within those clusters were different in the seed and spike. For example, sucrose peaks at 4 DAF in spike and dips in seed (Fig. 4). One developmental stage which influenced metabolite levels was cellularization, especially in the seed. Many seed metabolites either accumulated or depleted during cellularization at 4 DAF and returned to their previous level once the grain filling started at 6 DAF (Fig. 4). Hexoses, glucose, and fructose accumulated in the night of the cellularization phase, which may indicate a mobilization of carbon for cell wall formation or energy production [34]. BCAAs, valine, leucine, and isoleucine, also accumulated in the seed on 4 DAF. BCAAs and threonic acid are the metabolites whose abundance were remarkably affected in the seed during cellularization. They may act as an alternative energy source through the electron flavoprotein complex [35–37], when there is a strong demand for non-structural carbons for cell wall synthesis. Contrarily, some metabolites, including sucrose and quinic acid, decreased in the seed during cellularization. Depletions of sucrose and quinic acid may be explained by catabolism of these substrates to meet the increased demands for cell wall components, such as hexoses and ferulic acid [38] (Fig. 4). Metabolites related to phenylpropanoid biosynthesis (quinic acid, shikimic acid, tyrosine) accumulated or depleted in the seed at 4 DAF, which may suggest the coordination of phenylpropanoid metabolism to increase the production of phenolic cell wall components, including ferulic acid (Fig. 4).

Cellularization is also associated with the metabolite levels in the flag leaves and bracts, where many metabolites accumulated during night 2 DAF, before being depleted at 4 and 6 DAF (Figs. 1 and 2). As 4 DAF coincides with the peak of cellularization activity in the endosperm, this depletion, seen in many metabolites, may represent increased translocation of metabolic substrates responding to an increase in sink pressure from the seed for cell division. Metabolomes responded differently in the flag leaves and bracts following cellularization. In the flag leaf, metabolites remained depleted after cellularization, while bracts metabolites were re-accumulated at 6 DAF. This difference might exist because the flag leaf begins to senesce during early grain development. In contrast, the bracts tissue does not senesce until later [39], probably enabling bract metabolism to recover from metabolite consumption. For both source tissues, amino acids were accumulated at 4 and 6 DAF and may represent nitrogen mobilization for the developing seed. After cellularization is complete and grain filling begins around 6 DAF, most of the amino acids measured in the seed accumulated, which may show the production of storage proteins in the developing endosperm. Many of these amino acids are known to be translocated, as well as storage protein components, such as glutamic acid, glutamine, aspartic acid, and methionine [40, 41]. GABA also accumulated at 6 DAF. GABA is a non-proteinogenic amino acid implicated in nitrogen sensing, possibly reflecting the general accumulation of amino acids during storage protein formation [42].

The effects of HNT on the seed metabolite levels can be attributed to accelerated seed development (Fig. 6). It has been reported that HNT shortens days to maturity in rice, negatively impacting rice endosperm quality [25]. We observed an accelerated grain weight increase until 14 DAF, but the control plants showed similar and higher grain weight later on at 21 DAF and maturity (Fig. 6a, b). This is likely due to a faster rate of grain development with a shorter duration of grain filling, ultimately resulting in lighter grains at maturity, as reported in the previous study [24]. The reduced grain weight was probably the major reason for the yield loss under HNT (Fig. 6d) since the grain number was not significantly affected by HNT (Fig. 6c). This result suggests that HNT affected grain filling rather than seed number. Additionally, grain development impacts metabolite accumulations [32, 33]. Prominent increases and decreases in metabolite abundance were observed two days earlier under HNT than in the control condition (Fig. 7). Sugars were one group of metabolites whose time courses were accelerated under HNT in the seed, and it was previously shown that HNT disrupts sugar metabolism [24, 25, 43]. Sugars are metabolic substrates for cellularization, providing the carbon for the majority of cell wall components. Under HNT, hexoses peaked at night-time 2 DAF in the seed. Under control conditions, this occurred two days later at 4 DAF, presumably related to high carbon demand from cell wall formation. Besides accelerated time courses, the amount of sugars in seed also increased under HNT, which may represent the enhanced assimilate translocation under HNT as reported earlier [43], substrate buildup due to impaired cell wall construction, or to protect the cell from damage [44]. The time course of trehalose accumulation was also accelerated in seeds. Trehalose levels can indicate the abundance of its metabolic precursor, trehalose-6-phosphate. Trehalose-6-phosphate has been shown to be involved in signaling sugar availability and carbon partitioning [45, 46]. Also, in the seed, trehalose’s time course is accelerated similarly to the other sugars. These observations indicate coordination of carbon metabolism two days ahead of the control condition. A phenylpropanoid pathway product, ferulic acid, is also a significant component of endosperm cell walls [38]. Under HNT, the timing of the accumulation of several phenolic metabolites in the same pathway as ferulic acid (quinic acid and shikimic acid) shifted ahead of two days (Figs. 3 and 7). Several amino acids share the tendency as well, suggesting that the timeline of nitrogen demand in the seed is also accelerated under HNT. This perturbation of nitrogen metabolism may be related to the increased protein and lipid but decreased starch accumulation in mature wheat grains under HNT [24]. However, the accelerated wheat grain metabolic time courses under HNT need to be validated by further experiments to assess the developmental progression of the seed in parallel with metabolic profiling in higher time resolution.

We discovered that the most prominent effect of HNT on flag leaves and bracts was the creation, exaggeration, attenuation, or reversal of day and night oscillations. Particularly interestingly, sucrose under HNT had its day/night oscillation abolished in both flag leaves and bracts (Fig. 7c). Changes in the day/night oscillation of these metabolites may underlie the daytime metabolic changes induced by HNTs. There are several possible explanations for these effects of HNT on day/night oscillations. HNT may affect the plant’s circadian rhythm [47], leading to changes in day/night metabolic programs. Another explanation is that the altered sink strength of the seed during the night may affect metabolic abundances in source tissues during the day, leading to the activation and suppression of metabolic pathways to compensate for altered metabolic demands. For example, reversed day/night accumulation of glucose in flag leaves by HNT on 2 and 4 DAF (Fig. 7b) may be related to the enhanced glucose accumulation in the seeds partly due to the earlier glucose accumulation event that occurred on 4 and 6 DAF in the control condition. Glucose in flag leaves may be consumed or transported to support sugar accumulation in seeds during the night, and its daytime production may be enhanced to compensate for the higher glucose demand. Supporting this connection between source and sink metabolism, nearly all metabolites whose day/night oscillations were affected by HNT in the source tissues are related to cell wall formation in the seed, specifically sugars and phenylpropanoid substrates. However, these possible mechanisms must be experimentally tested by multiple experiments, such as metabolite profiling under various light regimes, including continuous light and dark, to test the involvement of circadian control and carbon tracer experiments to analyze the metabolite exchange between tissues.

Previous studies, such as [24] have used the spike as a proxy for the seed for HNT studies. The assumption was reasonable since we found the metabolic time courses of the spike were similar to those in the seed. We also found that HNT affects sugar metabolism, especially trehalose, both the seed and spike, similar to the spike results from [24].

## Conclusion

Our study provided a multi-tissue atlas for the metabolite profile of wheat during early grain development and how that profile changes when subjected to HNT stress. The day/night metabolite profiles at the dates representing early grain development revealed: (1) the seed metabolite profile is developmental stage dependent rather than the time in the day, (2) two photosynthetic tissues, bracts and flag leaves, have different metabolic roles in early seed development with bract may serve as metabolite reserve during the night, (3) HNT hastens metabolic changes in seeds, likely associated with the previously described acceleration of seed development [24], and (4) HNT perturbs day/night metabolite accumulation in photosynthetic tissues. (1) and (2) provided basic insights to understand the complex metabolic interactions between tissues during early grain development, and (3) and (4) suggest possible mechanisms of grain yield loss under HNT. These discoveries were only possible by day/night sampling and highlighted the importance of analyzing metabolite profiles in a day/night context, especially to understand HNT responses.

Future studies can use metabolic flux analysis and metabolic profiling of phloem exudate to confirm which source tissues are supplying which metabolic substrates translocated to the seed, and how those relationships change under stress. It may also be interesting to move plants from the HNT to the control condition to see if and how the metabolite profiles alter. Finally, although we observed that the levels of some metabolites appear to differ between day and night, these differences should be characterized more rigorously. These studies will enhance understanding of the source/sink relationships in wheat grain development, potentially leading to the generation of cereal crops with higher yields and more tolerance to stresses, including HNT. Elucidating the mechanisms underlying the perturbation of day/night oscillation of sugar metabolism in source tissues under HNT may lead to improving wheat HNT tolerance.

## Methods

### Plant material and growth conditions

The wheat (Triticum aestivum) cultivar Everest seeds were provided by Dr. Krishna Jagadish at Kansas State University. Everest is a commercially successful variety widely grown in the regions with expected HNT events that has been shown to be sensitive to HNT stress [48]. The experiment was conducted at the University of Nebraska-Lincoln Agronomy and Horticulture greenhouse facility. Vernalized seedlings were acclimated and transplanted to 4-inch square pots filled with a 4:1:1:1 mixture of soil, sand, peat, and perlite. Established seedlings were regularly provided non-growth limiting doses of liquid fertilizer (20-10-20 NPK with micronutrients), and pots were bottom irrigated using a shallow tub. During late October / early November 2019, natural sunup to sundown was 8:15 to 18:45. Artificial lighting from 400 W metal halide fixtures was added to extend the day length to 12 h, 8:00 to 20:00, and all plants were grown to the reproductive stage under the average control day temperature of 23 °C (ranging between 25 and 20 °C), and average night temperatures of 13 °C (ranging between 15 and 12 °C). Flowering plants assigned to the HNT stress treatment were transferred at anthesis to an adjacent room with an average day temperature of 23 °C (ranging between 25 and 20 °C) and an average night temperature of 23 °C (ranging between 25 and 22 °C) and kept under the HNT condition until physiological maturity. For grain filling data, dry weights of five seeds from primary spikes were determined for four replicates (pots) at 7, 14, and 21 days after anthesis under control and HNT conditions. Grain yield (dry weight in grams) and grain number of primary spikes were also measured on ten replicates under both environments at physiological maturity. Sampling for metabolite profiling was carried out between October and November 2019. Records of greenhouse temperatures can be seen in Supplemental Fig. S5.

### Tissue sampling and metabolic profiling

On the first day of anthesis, some wheat spikelets with a visible anther on the central portion of the primary spike were marked with a marker pen. After the first day of anthesis, no additional spikelet was marked. Only the marked spikelets were sampled to ensure the DAF. Seeds (embryo and endosperm) and bracts (lemma, palea, and awn) were pooled for each biological replicate. After marked spikelets were removed, the rest of the primary spike (rachis, bracts, and fertilized and empty spikelets) was sampled. One flag leaf was collected from the same primary tiller as all other samples. Sampling was done at 2, 4, and 6 DAF at 12:00 (4 h after light) and 23:00 (3 h after dark). We chose these sampling times because several hours have passed after the transition to day or night, which should be long enough to analyze the effects of day and night metabolism. Night-time sampling was conducted under green light. Five biological replicates were collected for each treatment and time point, with each biological replicate coming from the primary tiller of a single plant. After collection, the tissue was immediately frozen in liquid nitrogen and transferred to -80 °C. Frozen samples were ground to a fine powder with a TissueLyserII (Qiagen, Hilden, Germany). Up to 50 mg of each tissue was aliquoted for extraction by the methanol/water/chloroform method described by [49]. Fifty µl of the polar upper phase were dried by vacuum concentrator before derivatization by methoxyamine hydrochloride (Sigma-Aldrich, Milwaukee, WI) and N-Methyl-N-(trimethylsilyl) trifluoroacetamide (CovaChem, Loves Park, IL) as in [49]. A reference sample was produced for peak annotation purposes by mixing 5 µl of each derivatized sample. Gas chromatography was done with a 7200 GCQTOF System (Agilent Technologies, Santa Clara, CA, United States) using an HP-5MS UI GC Column (30 m × 0.25 mm, 0.25 μm; Agilent), helium carrier gas (Ultra High Purity 99.999%, Matheson, Lincoln, NE) at a flow rate of 1 ml min^−1^, an injection volume of 1 µl by splitless or split 50 settings. We used a GC oven temperature program consisting of initial temperature 80 °C with a hold time of 2 min, a ramp of 15 °C min^−1^, and a final temperature of 330 °C with a hold time of 6 min. We used electron ionization with an EI energy of 70 eV, an ion source temperature of 280 °C, an acquired mass range of 70 to 600 amu, and a 50 Hz scan rate. Chromatogram evaluation was conducted according to [50]. Peaks were annotated using Agilent Masshunter Unknown Analysis software with the Fiehn GC/MS Metabolomics RTL Library (G1676AA; Agilent) as a reference library to produce the batch-specific metabolic library files for use in peak identification and quantification. We quantified the peak height using Masshunter QTOF Quantitative Analysis software (Agilent). See Supplemental Data 1 for ion m/z and retention indexes used for metabolite annotation and quantification. Raw peak height measurements were then normalized by an internal ribitol standard and then by the fresh weight of each sample to represent the relative levels of metabolites. Relative metabolite levels were log2 and Z transformed for further analyses.

### Statistical analysis

All statistical analyses were conducted with the R platform (R Foundation for Statistical Computing, Vienna, Austria). Principal component analysis was conducted to analyze the similarity of global metabolite profiles among samples with prcomp function. PCA score plots were drawn by autoplot function in ggfortify package. Hierarchical clustering analysis was performed to group the metabolites showing similar time courses in abundance in individual tissues using the R package *pheatmap* using Euclidean distance. Line graphs were drawn by the R package *ggplot2*. To examine the effects of HNT, day/night, and DAF, we used the R function *lm* to produce linear models. Those linear models were then analyzed by the *Anova* function from the package *Cars* using HNT, day/night, and DAF as categorical variables. *P*-values were adjusted using the Benjamini-Hochberg method with a cutoff value of 0.05 to determine significance. The effects of HNT treatment on the developing grain weights, and the average grain weights, grain number, and grain yield at physiological maturity were evaluated by *t*-test using ttest R function (*p* < 0.05).

### Supplementary Information

Supplementary Material 1.

Supplementary Material 2.

Supplementary Material 3.

Supplementary Material 4.

Supplementary Material 5.

Supplementary Material 6.

## Data Availability

All data generated or analysed during this study are included in this published article and its supplementary information files. The raw chromatography data are available from the corresponding author on reasonable request.

## Abbreviations

HNT: High night temperature
DAF: Days after fertilization
BCAA: Branched chain amino acid
GABA: γ-aminobutyric acid
