# Supplementary material for: Tissue- and time-dependent metabolite profiles during early grain development under normal and high night-time temperature conditions
Source: BMC Plant Biol. 2024 Jun 18;24:568. doi: 10.1186/s12870-024-05190-6 (PMC11184705; doi:10.1186/s12870-024-05190-6)
Supplement: Supplementary file 1 — Supplementary Material 1. [file 12870_2024_5190_MOESM1_ESM.pdf]

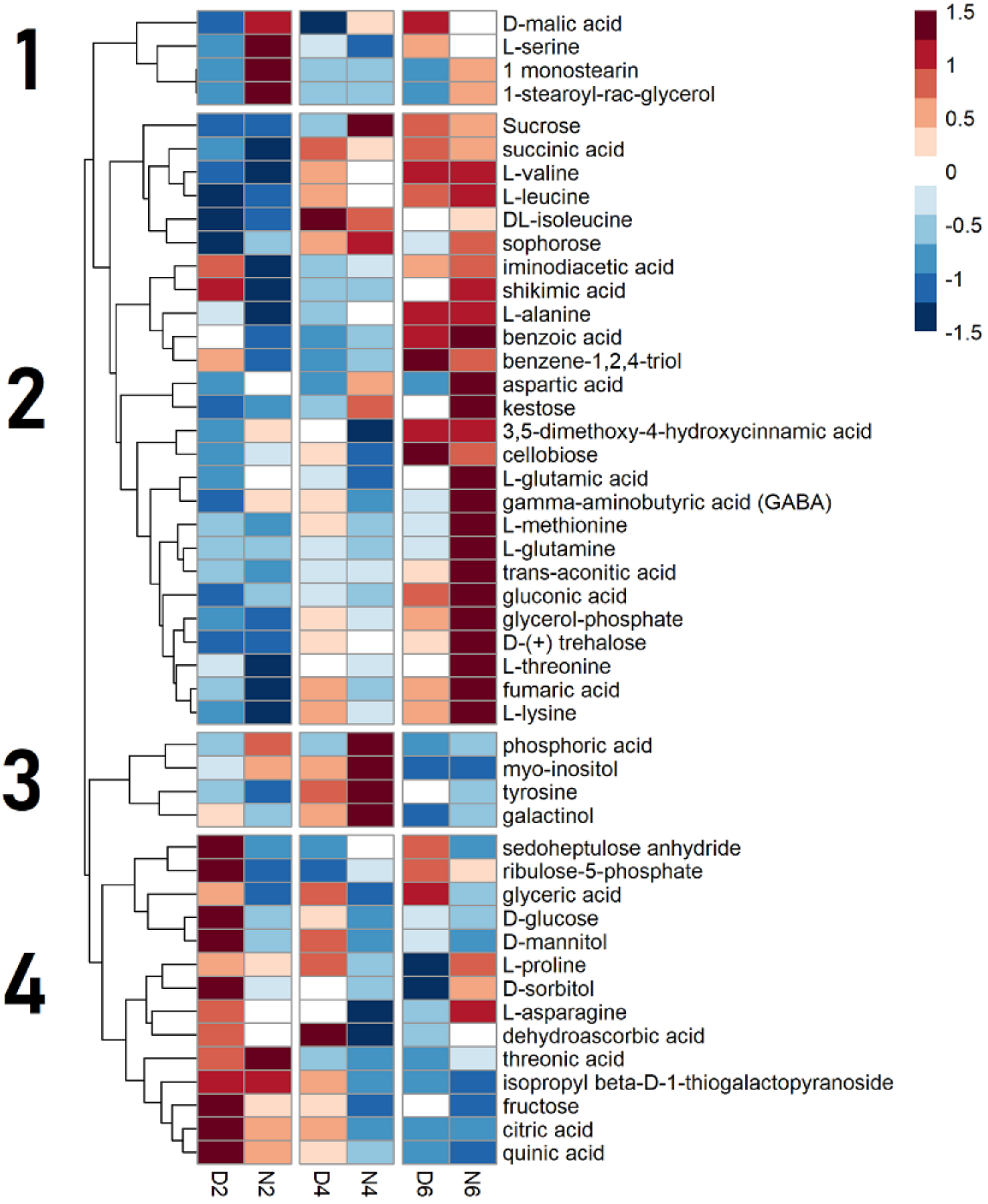

Supplemental Fig. S2. Time courses of metabolite abundance in spikes during early seed development. Each row represents a metabolite. Columns represent time points, where D or N represent day or night time sampling and 2, 4, and 6 represent the days after fertilization (DAF) of sampling. Each square, except the first column, is a mean of up to five biological replicates of relative metabolic abundances. Values in each row (metabolite) were $\log_2$ transformed, and Z transformed with a mean of zero and a standard deviation of 1. A color scale of red to blue represents metabolic abundances above and below the row mean respectively. Numbers to the left of the heatmap identify hierarchical clusters.
