# Supplementary material for: Tissue- and time-dependent metabolite profiles during early grain development under normal and high night-time temperature conditions
Source: BMC Plant Biol. 2024 Jun 18;24:568. doi: 10.1186/s12870-024-05190-6 (PMC11184705; doi:10.1186/s12870-024-05190-6)
Supplement: Supplementary file 2 — Supplementary Material 2. [file 12870_2024_5190_MOESM2_ESM.pdf]

Supplemental Fig. S3. Time courses of the abundance of all metabolites in the control growth condition. Y axis represents relative metabolic abundance. X axis represents time point, where D or N represent day or night time sampling and 2, 4, and 6 represent the days after fertilization (DAF) of sampling. To represent the days between data collection, solid rectangles were overlaid over the chart, with dull yellow representing day, and dull blue representing night. Data is represented as mean values at each time point, as well as flanking lines representing one +- standard error, from up to five biological replicates. Colors represent tissues. Values in each metabolite, including all tissue types, were log2 transformed, and Z transformed with a mean of zero and a standard deviation of 1.

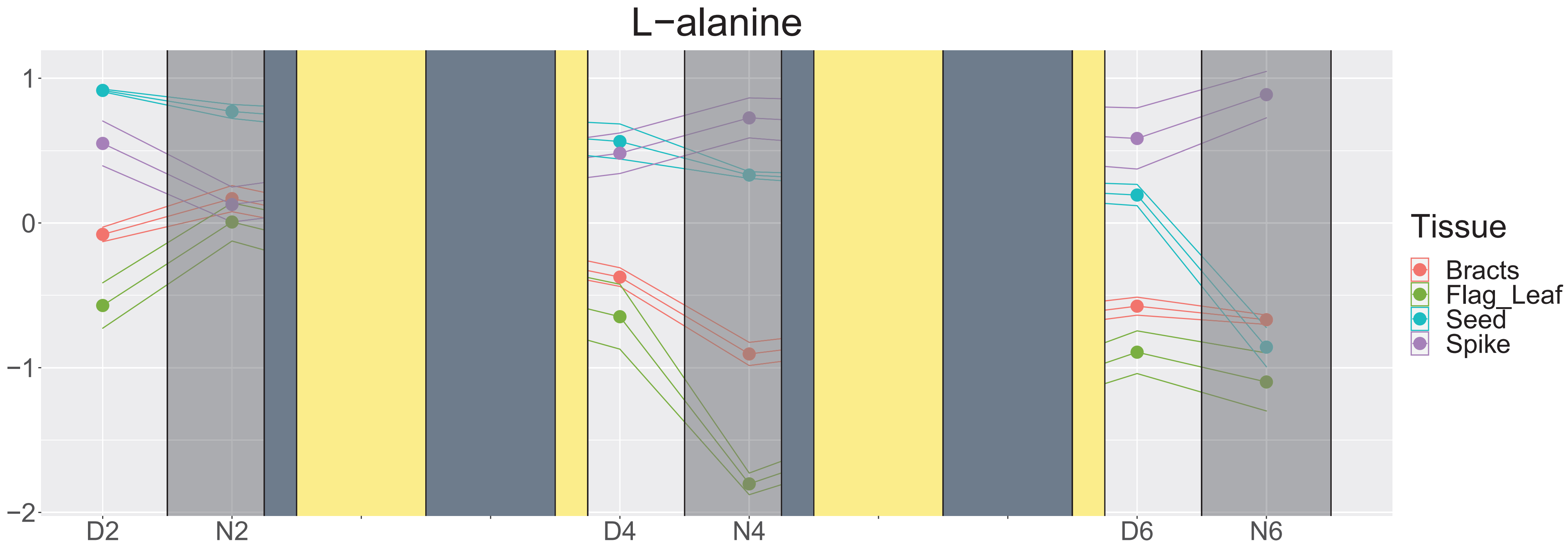

L−alanine
1
0
−1
−2
D2
N2
D4
N4
D6
N6
Tissue
Bracts
Flag_Leaf
Seed
Spike

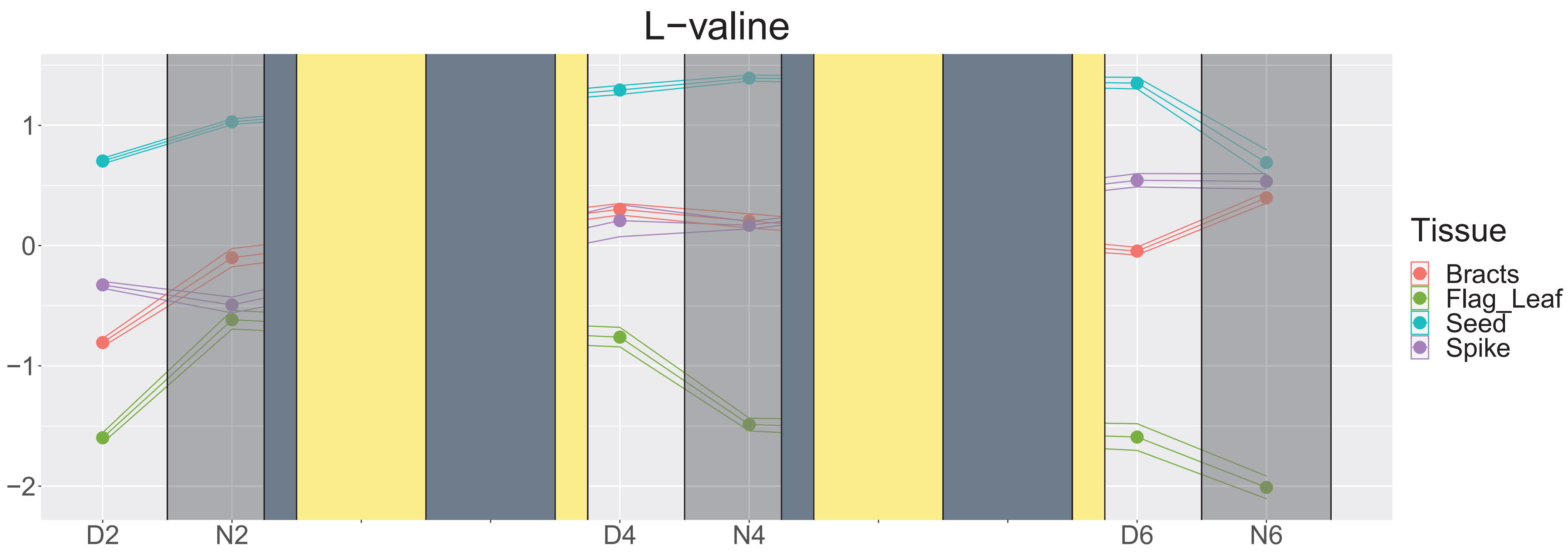

L−valine
1
0
−1
−2
D2
N2
D4
N4
D6
N6
Tissue
Bracts
Flag_Leaf
Seed
Spike

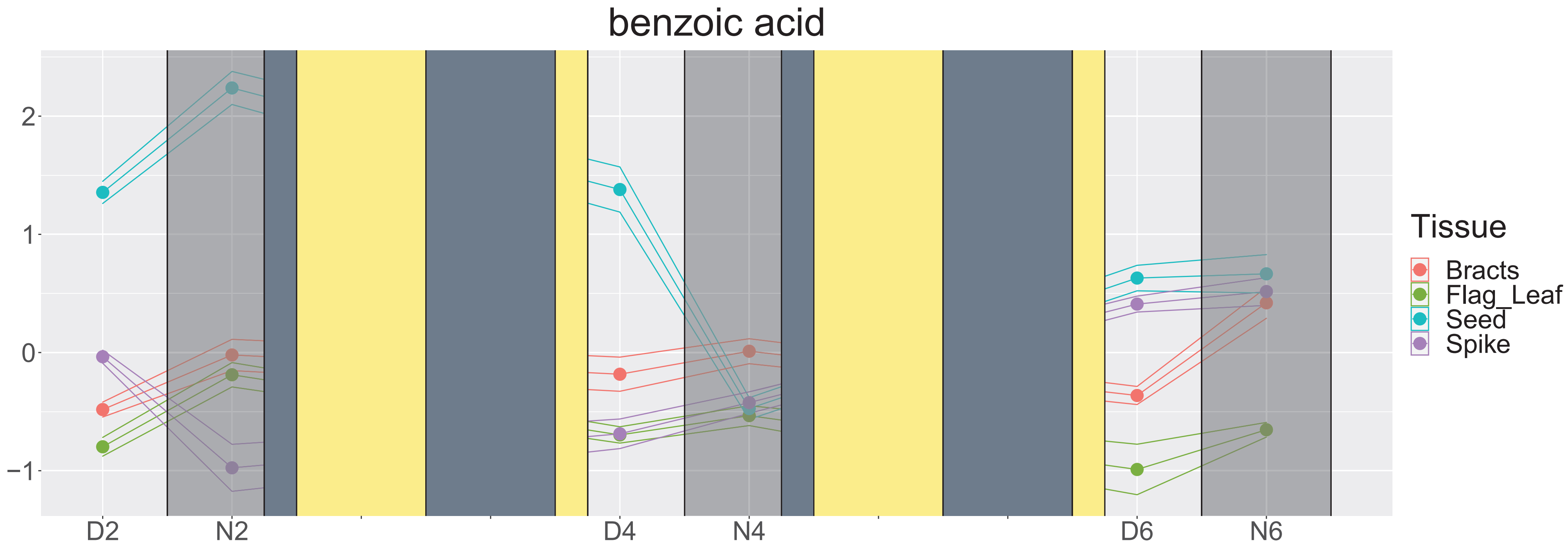

benzoic acid
2
1
0
−1
D2
N2
D4
N4
D6
N6
Tissue
Bracts
Flag_Leaf
Seed
Spike

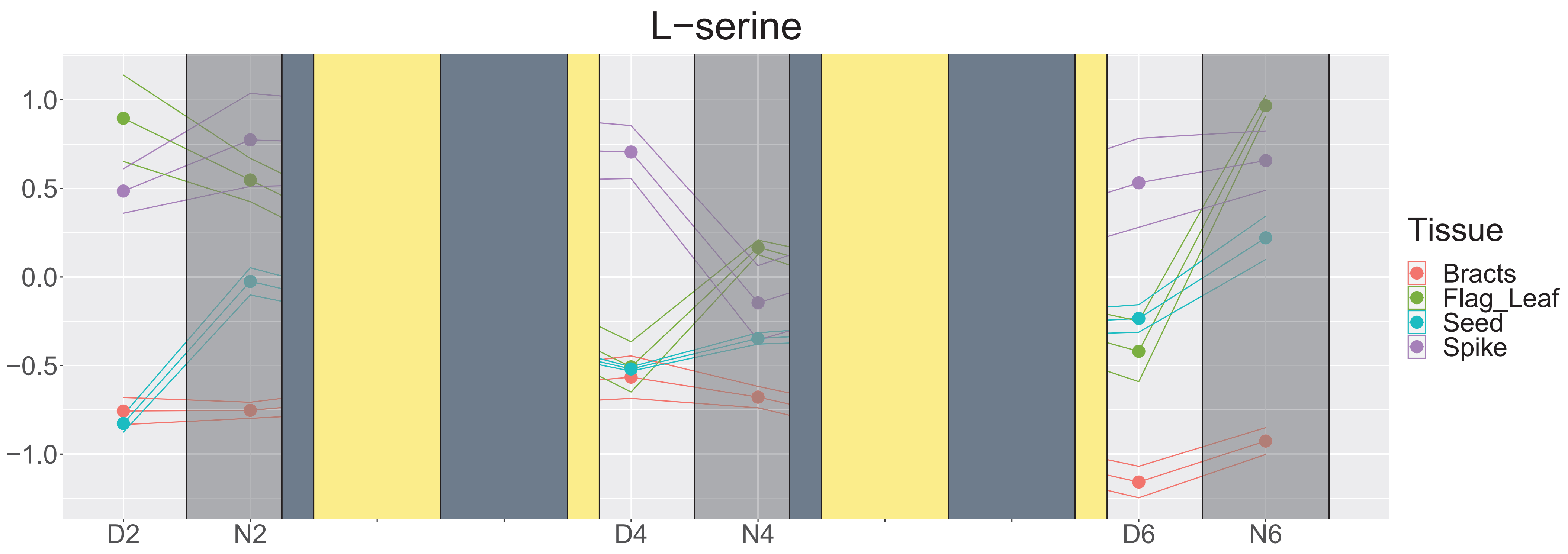

L−serine
1.0
0.5
0.0
−0.5
−1.0
D2
N2
D4
N4
D6
N6
Tissue
Bracts
Flag_Leaf
Seed
Spike

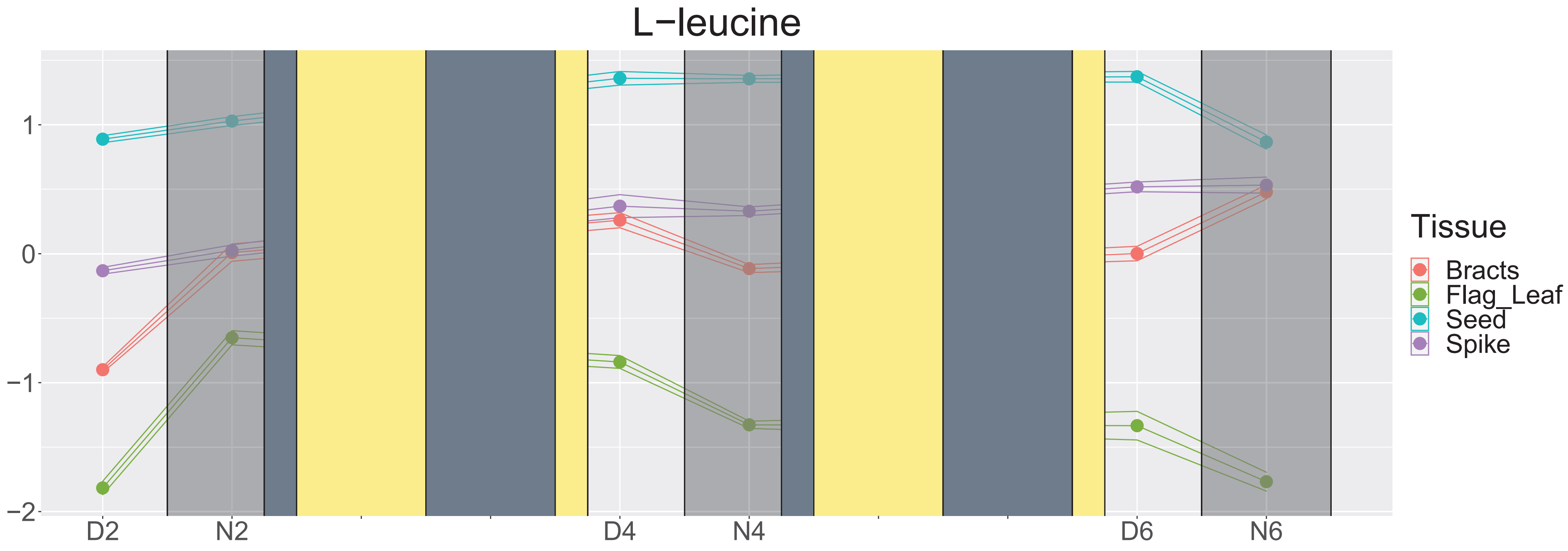
L−leucine
1
0
−1
−2
D2
N2
D4
N4
D6
N6
Tissue
Bracts
Flag_Leaf
Seed
Spike

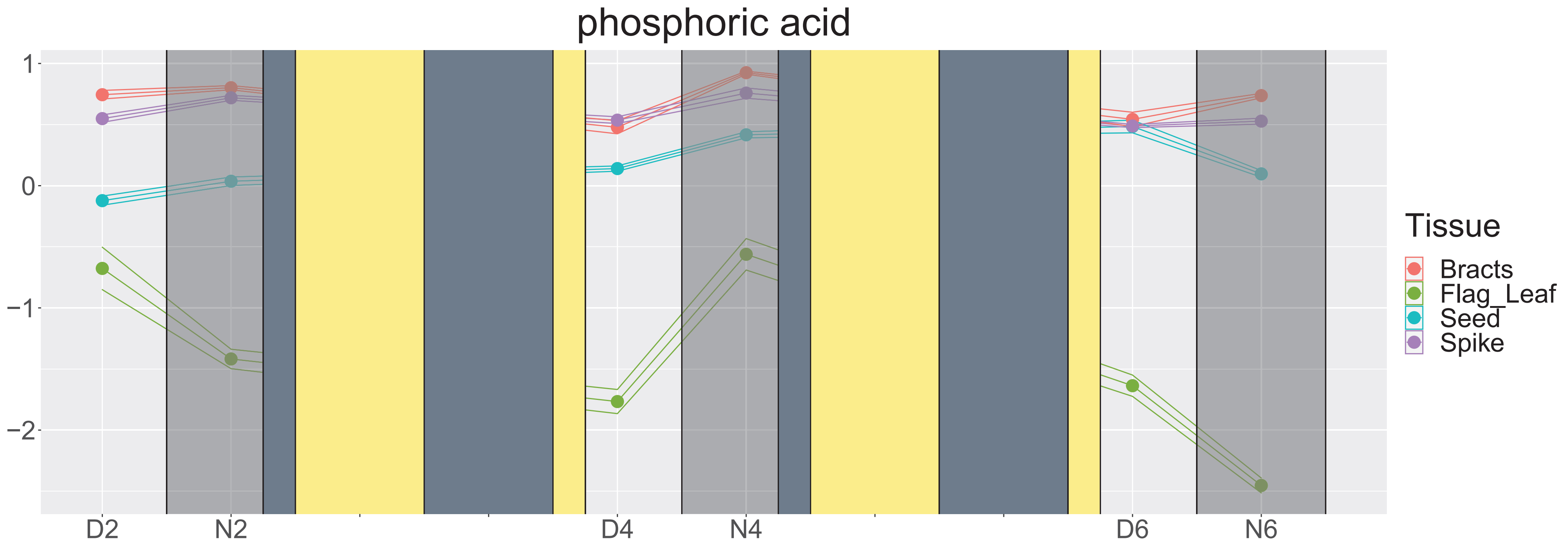
phosphoric acid
1
0
−1
−2
D2
N2
D4
N4
D6
N6
Tissue
Bracts
Flag_Leaf
Seed
Spike

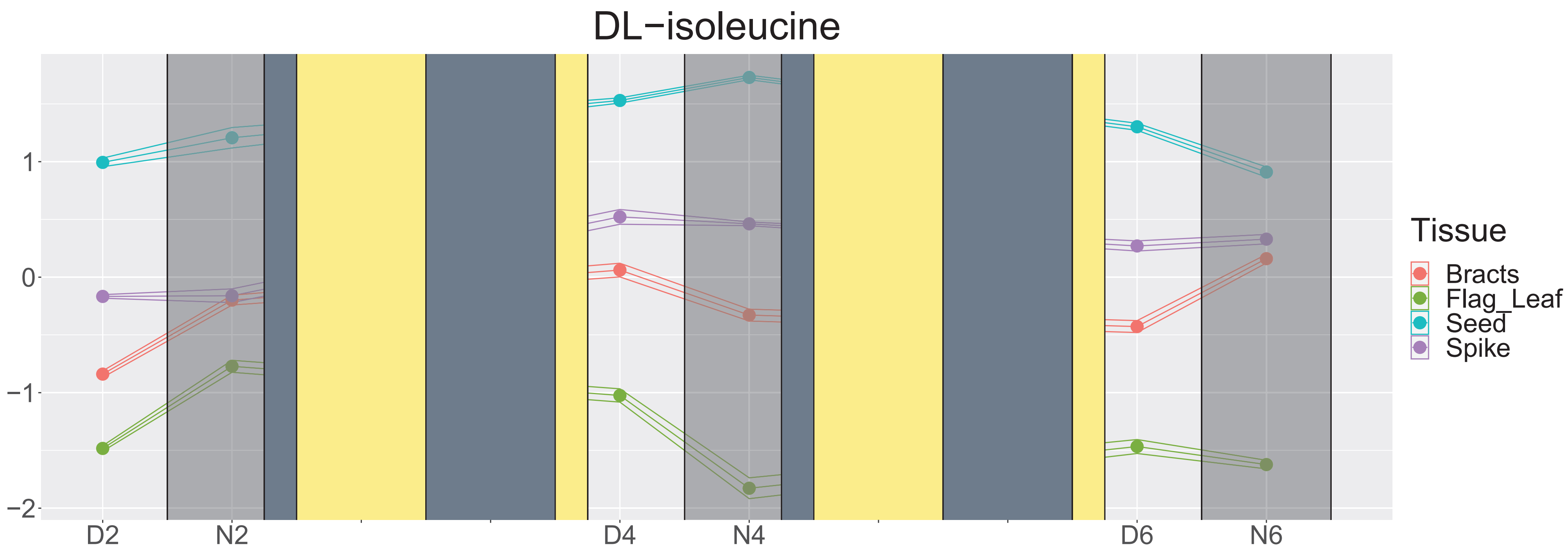
DL−isoleucine
1
0
−1
−2
D2
N2
D4
N4
D6
N6
Tissue
Bracts
Flag_Leaf
Seed
Spike

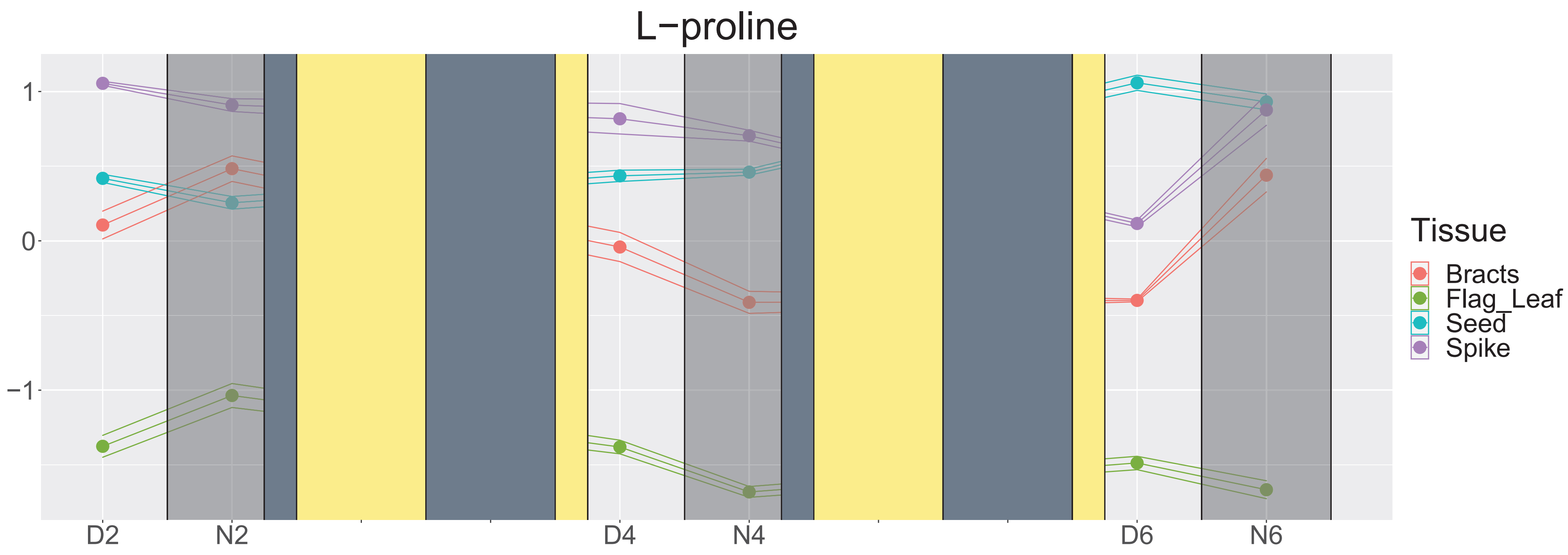

L−proline
1
0
−1
D2
N2
D4
N4
D6
N6
Tissue
Bracts
Flag_Leaf
Seed
Spike

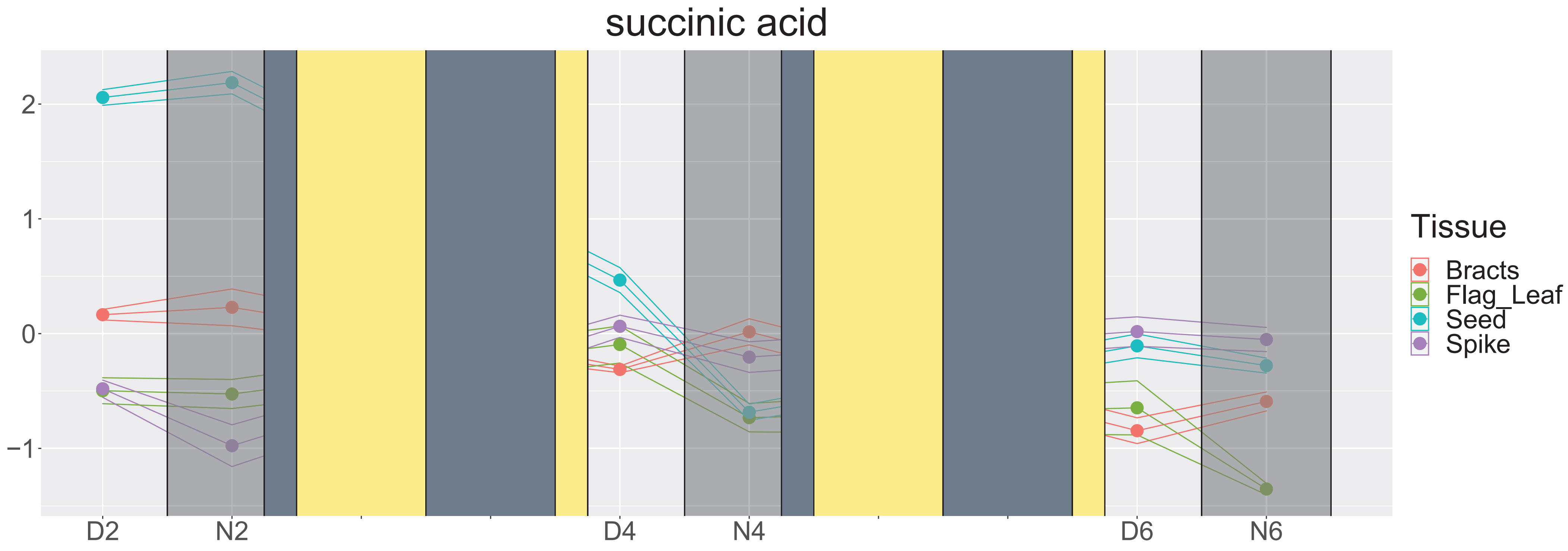

succinic acid
2
1
0
−1
D2
N2
D4
N4
D6
N6
Tissue
Bracts
Flag_Leaf
Seed
Spike

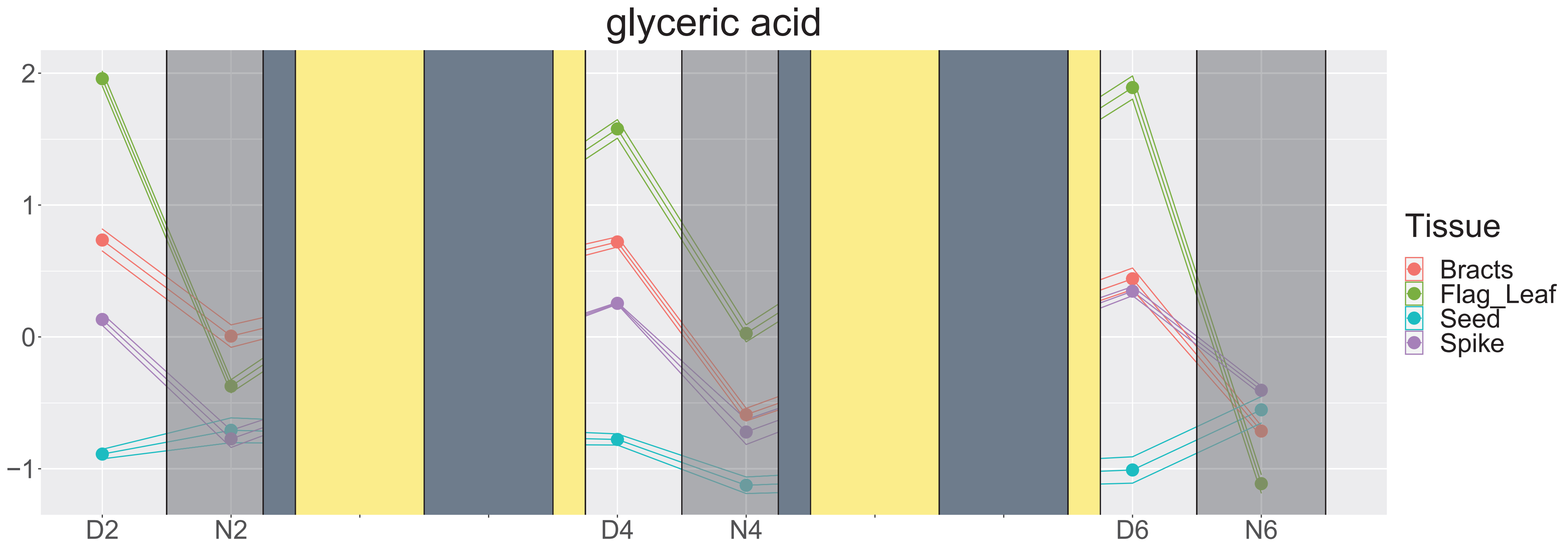

glyceric acid
2
1
0
−1
D2
N2
D4
N4
D6
N6
Tissue
Bracts
Flag_Leaf
Seed
Spike

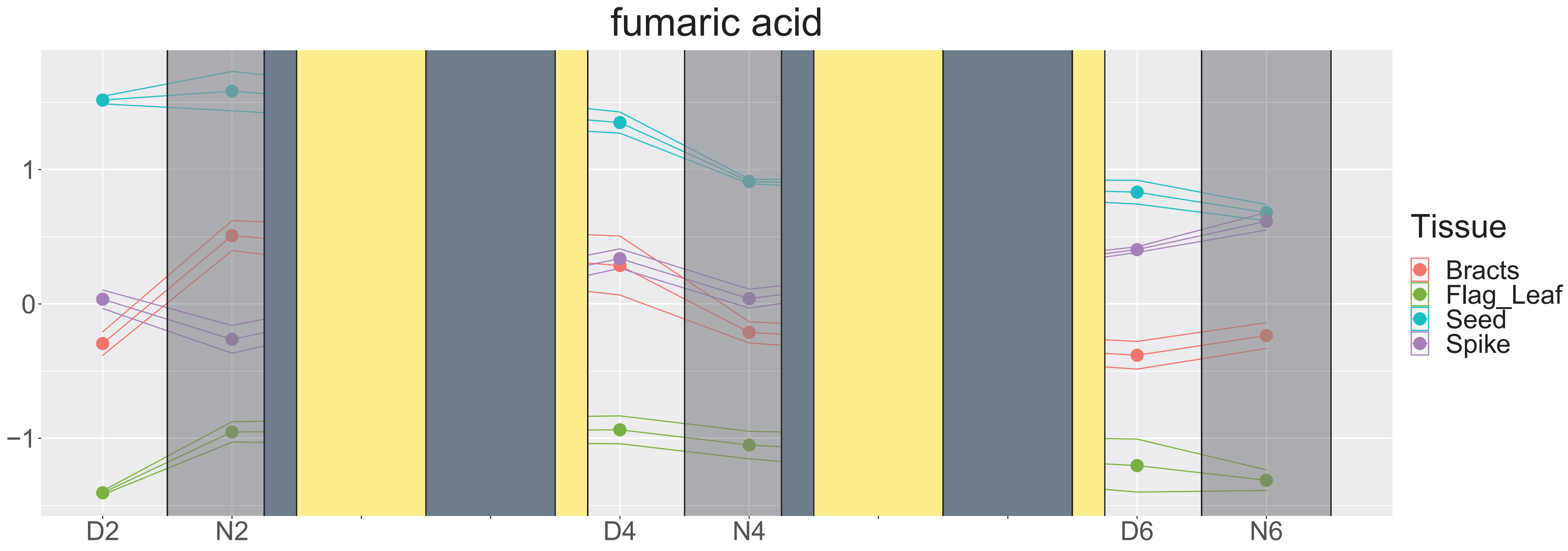

fumaric acid
1
0
−1
D2
N2
D4
N4
D6
N6
Tissue
Bracts
Flag_Leaf
Seed
Spike

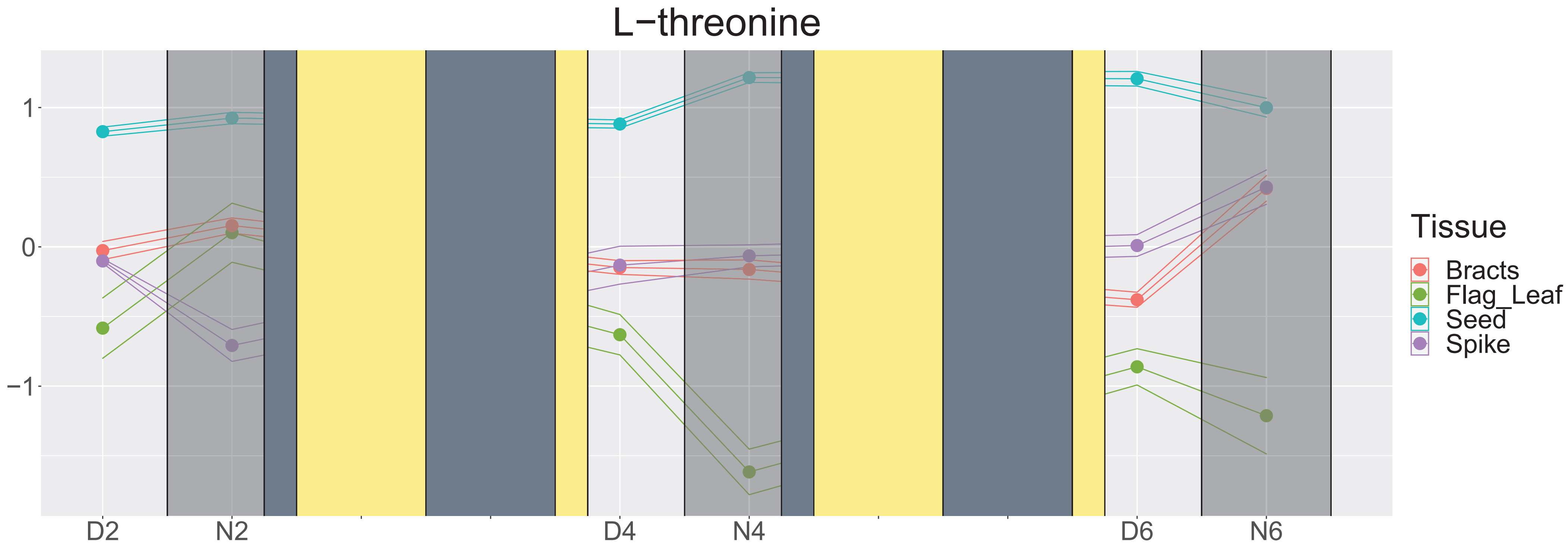
L−threonine
1
0
−1
D2
N2
D4
N4
D6
N6
Tissue
Bracts
Flag_Leaf
Seed
Spike

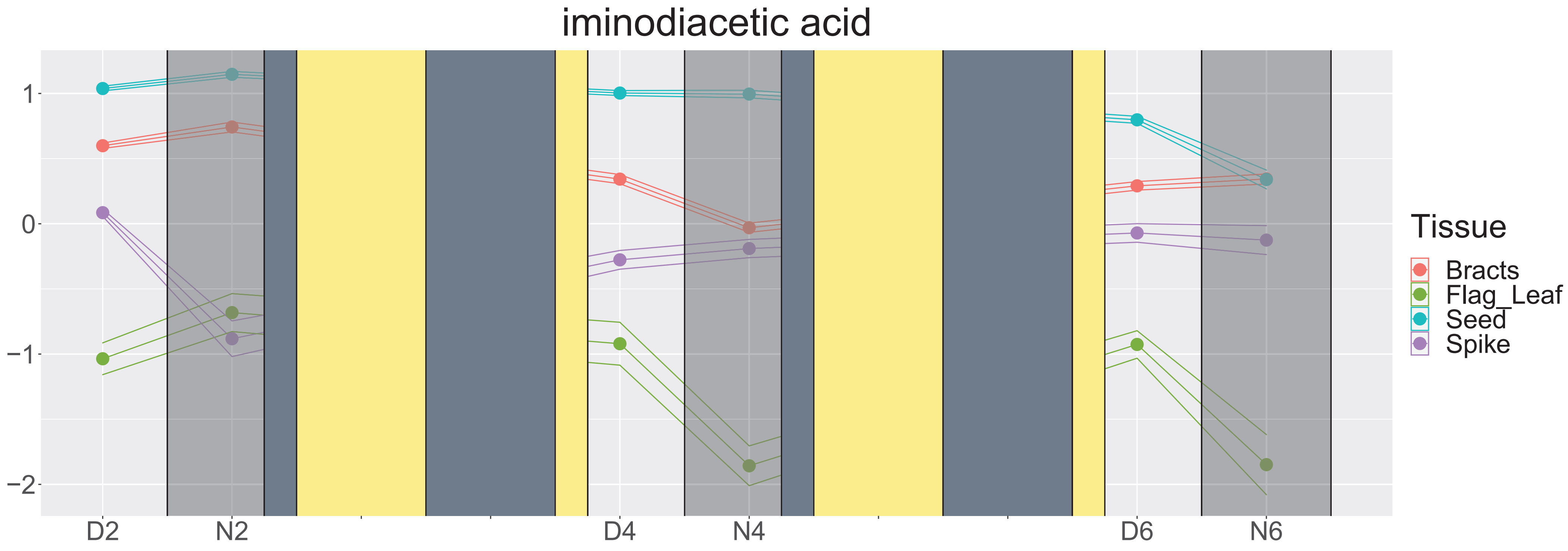
iminodiacetic acid
1
0
−1
−2
D2
N2
D4
N4
D6
N6
Tissue
Bracts
Flag_Leaf
Seed
Spike

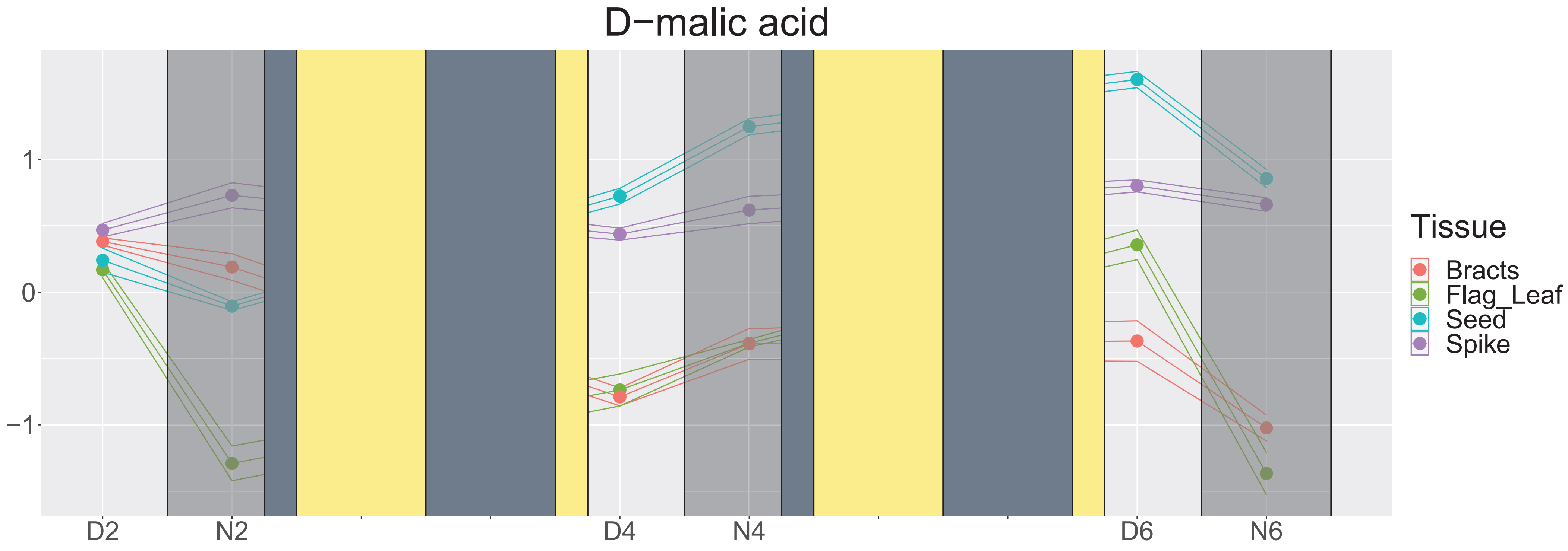

D−malic acid
1
0
−1
D2
N2
D4
N4
D6
N6
Tissue
Bracts
Flag_Leaf
Seed
Spike

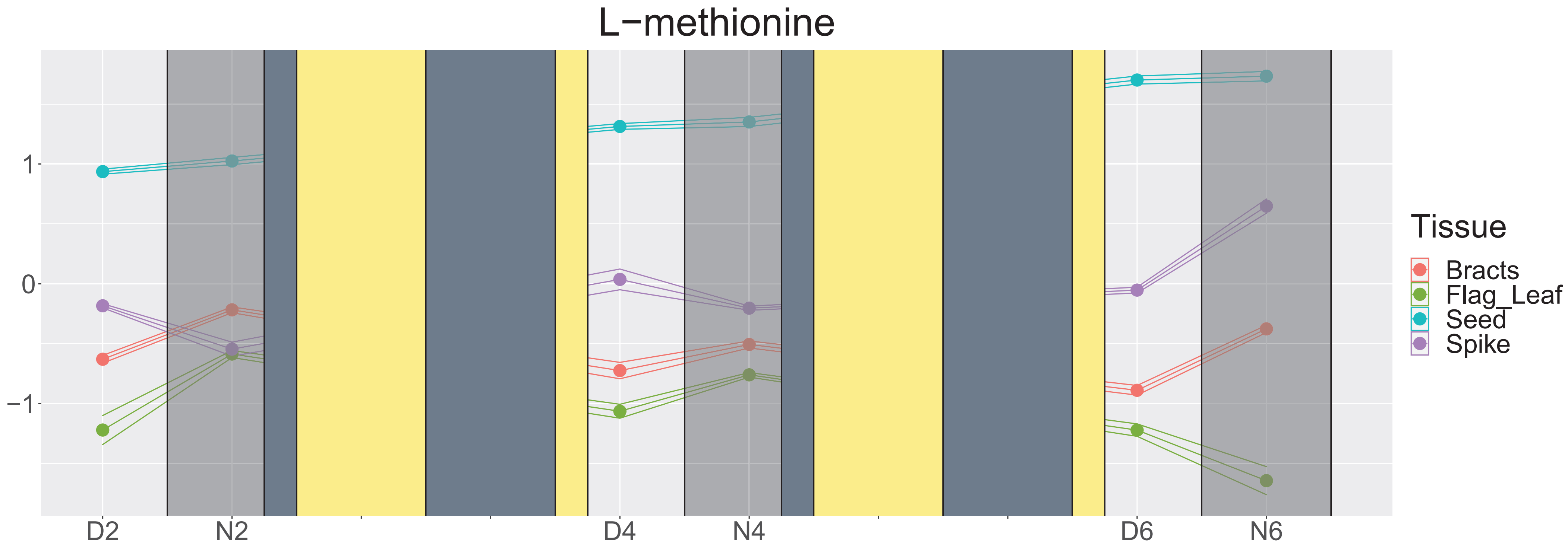

L−methionine
1
0
−1
D2
N2
D4
N4
D6
N6
Tissue
Bracts
Flag_Leaf
Seed
Spike

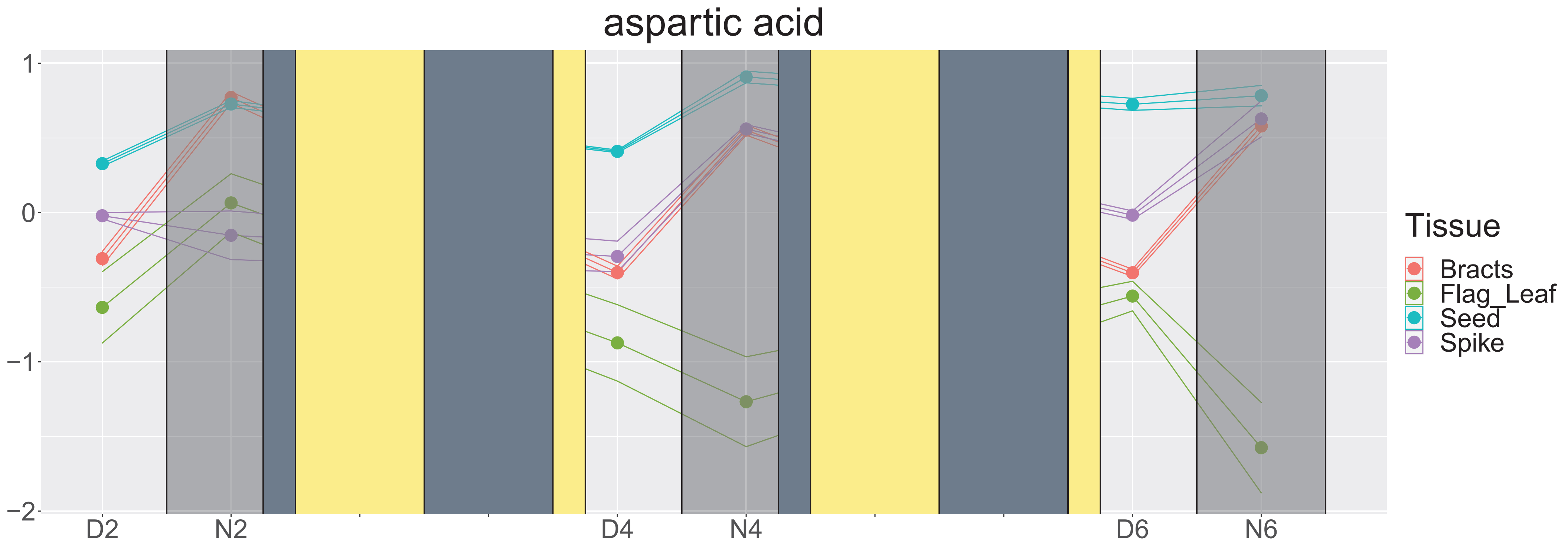

aspartic acid
1
0
−1
−2
D2
N2
D4
N4
D6
N6
Tissue
Bracts
Flag_Leaf
Seed
Spike

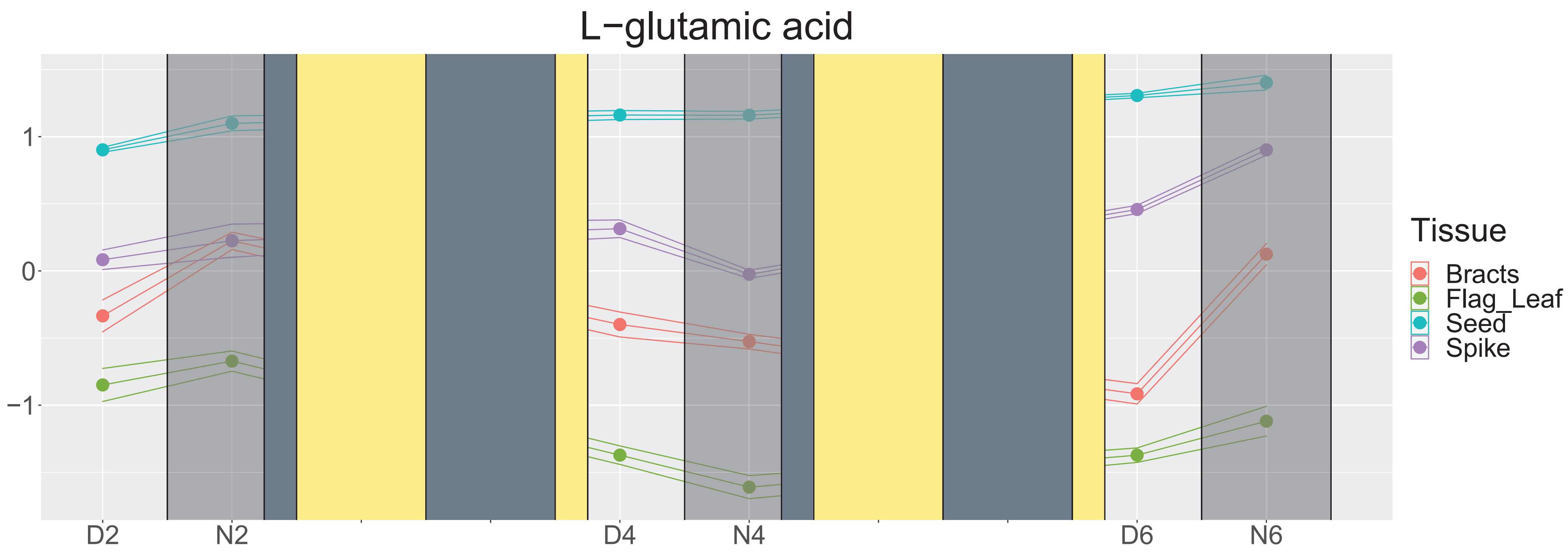

L−glutamic acid
1
0
−1
D2
N2
D4
N4
D6
N6
Tissue
Bracts
Flag_Leaf
Seed
Spike

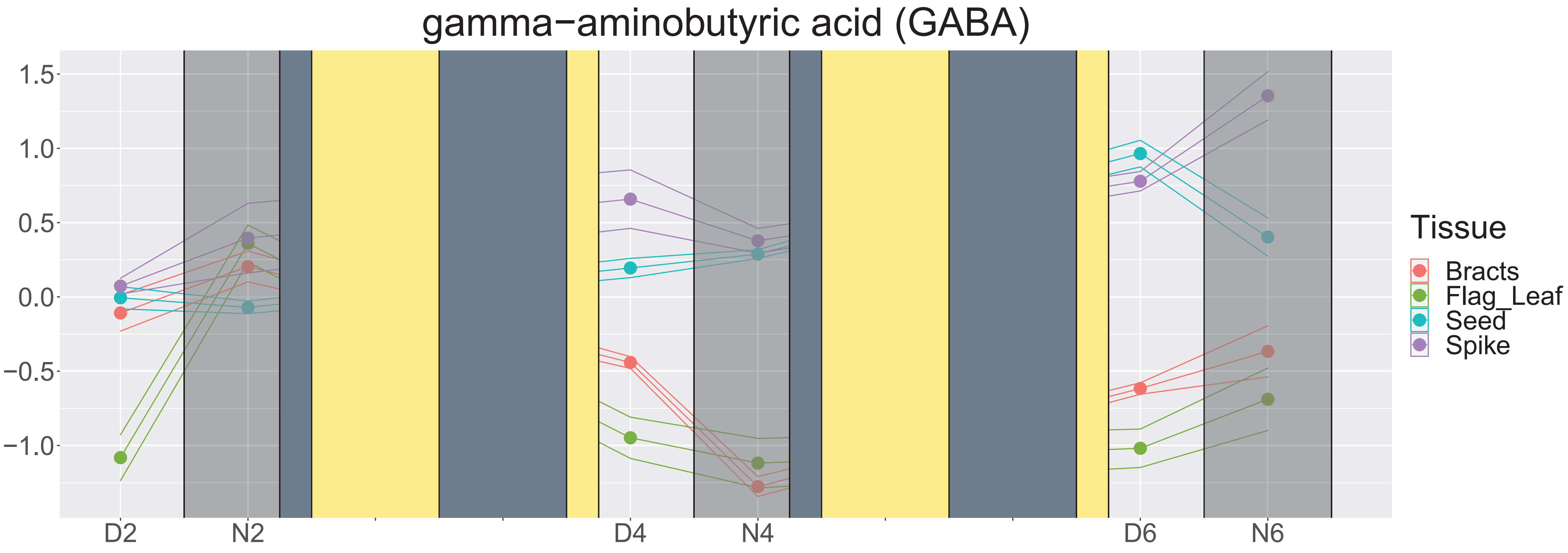
gamma−aminobutyric acid (GABA)
1.5
1.0
0.5
0.0
−0.5
−1.0
D2
N2
D4
N4
D6
N6
Tissue
Bracts
Flag_Leaf
Seed
Spike

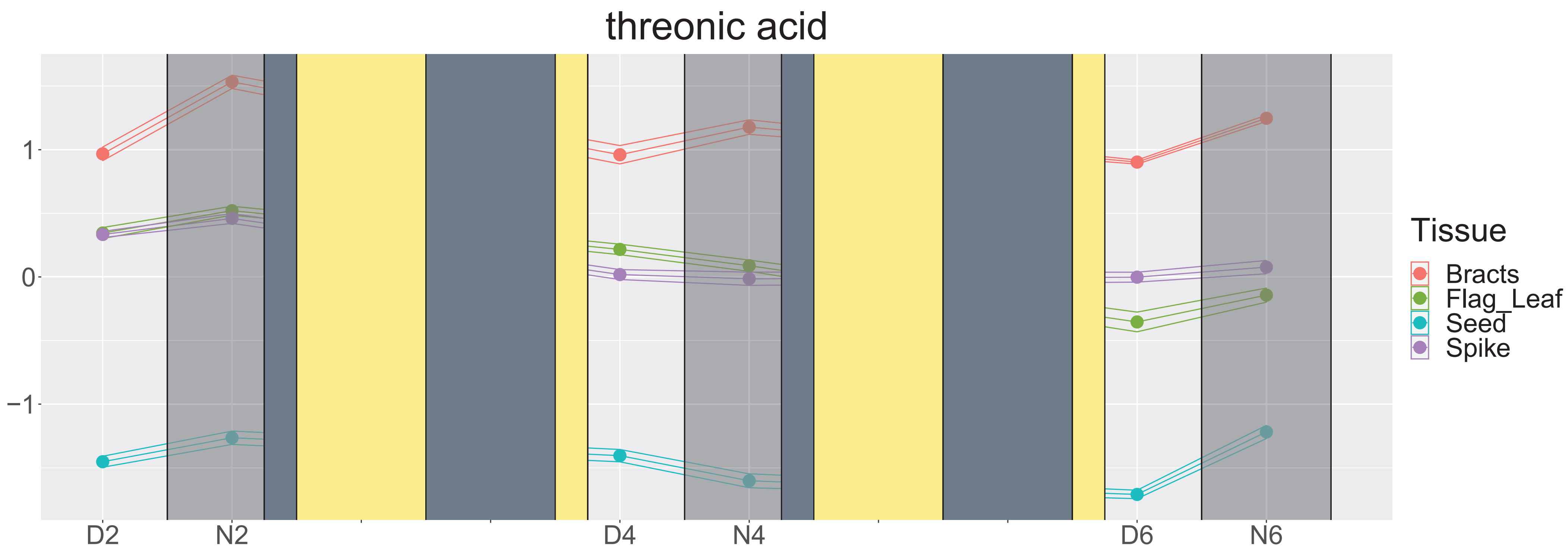

threonic acid
1
0
−1
D2
N2
D4
N4
D6
N6
Tissue
Bracts
Flag_Leaf
Seed
Spike

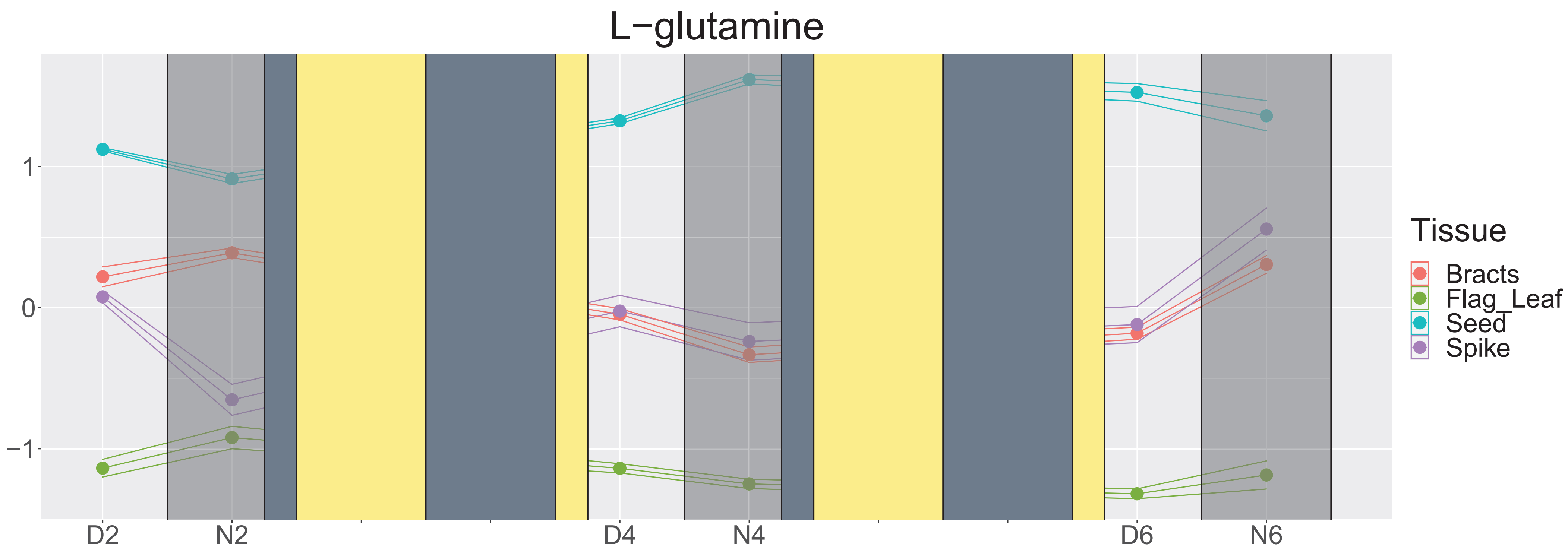

L−glutamine
1
0
−1
D2
N2
D4
N4
D6
N6
Tissue
Bracts
Flag_Leaf
Seed
Spike

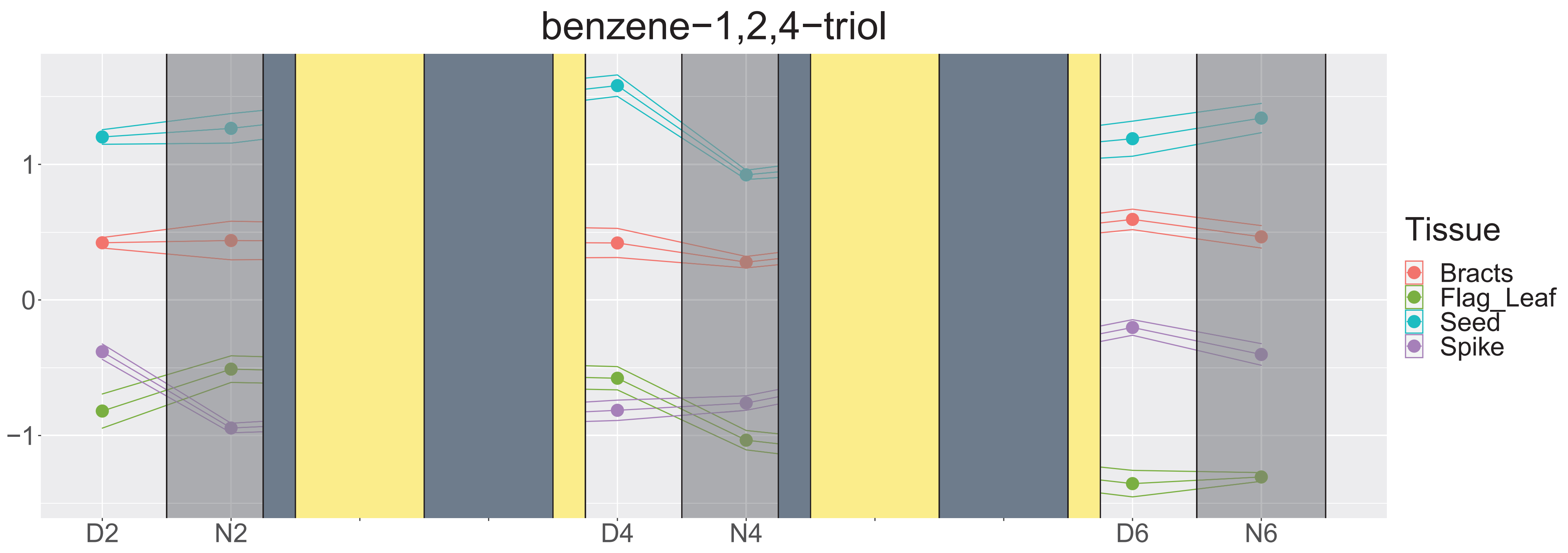

benzene−1,2,4−triol
1
0
−1
D2
N2
D4
N4
D6
N6
Tissue
Bracts
Flag_Leaf
Seed
Spike

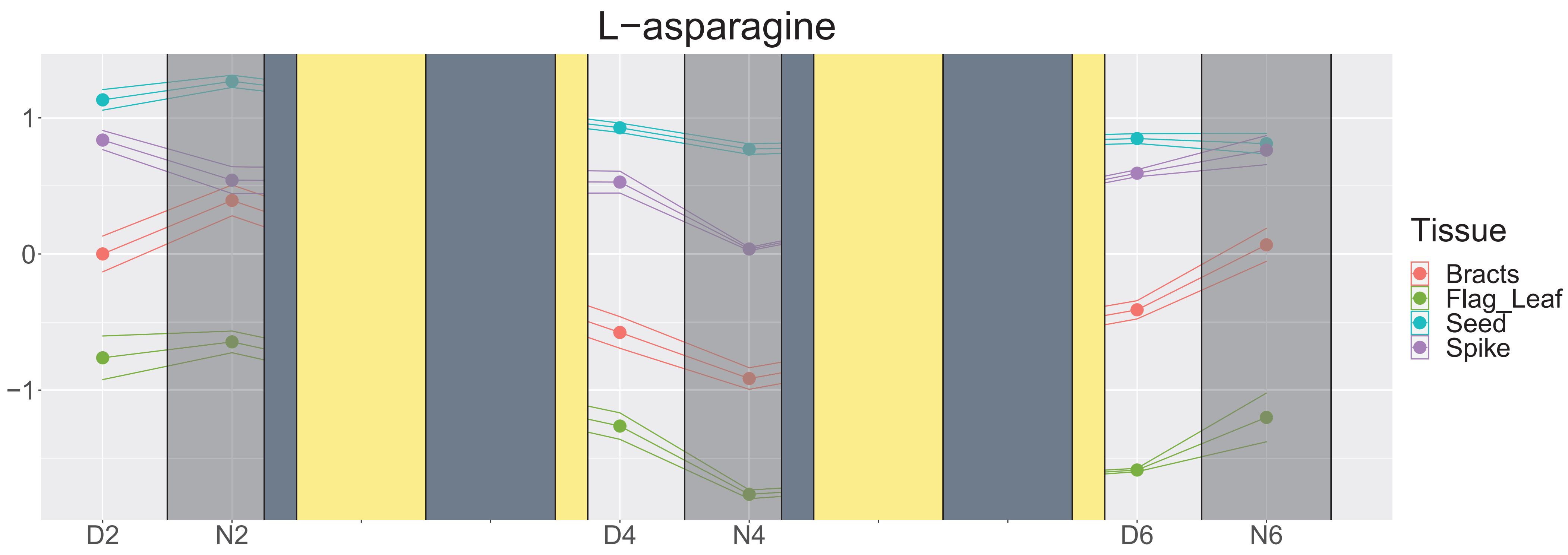
L−asparagine
1
0
−1
D2
N2
D4
N4
D6
N6
Tissue
Bracts
Flag_Leaf
Seed
Spike

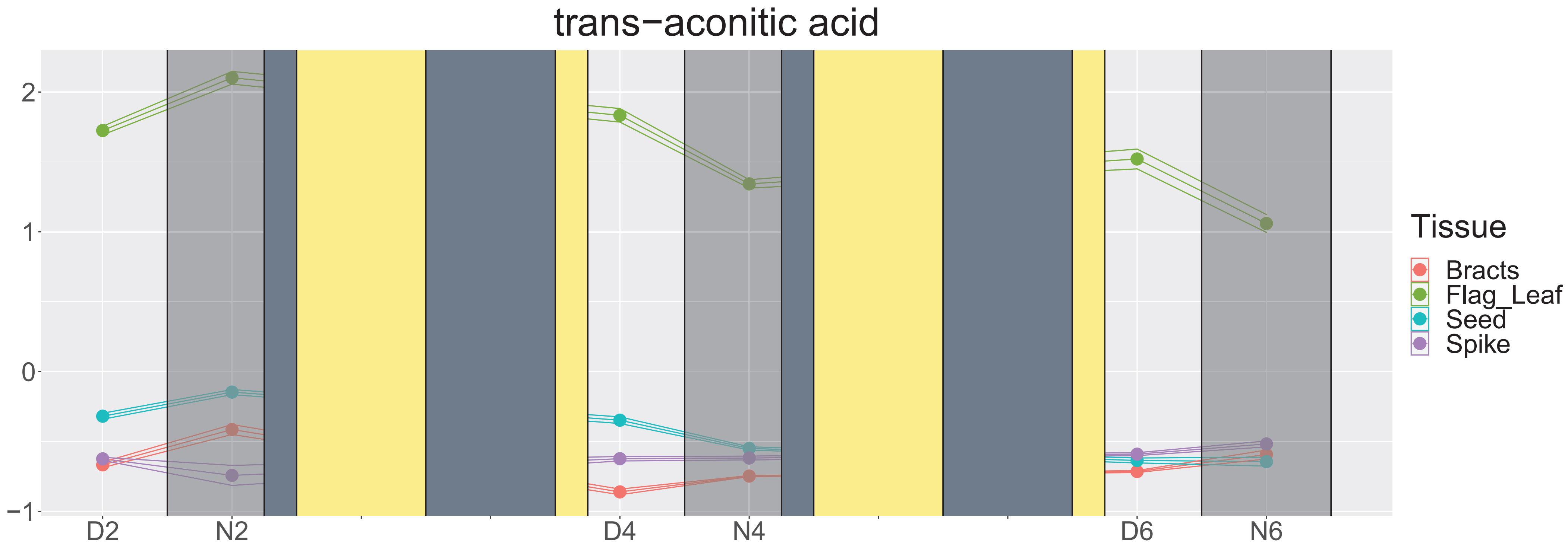

trans−aconitic acid
2
1
0
−1
D2
N2
D4
N4
D6
N6
Tissue
Bracts
Flag_Leaf
Seed
Spike

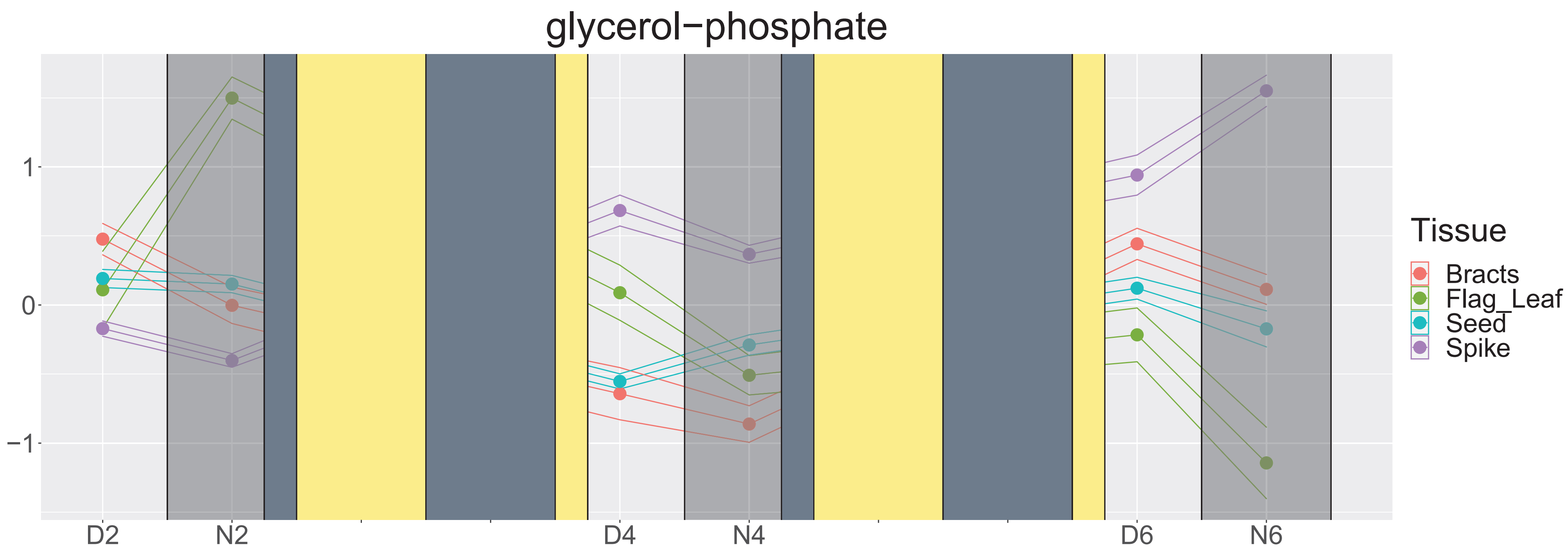

glycerol−phosphate
1
0
−1
D2
N2
D4
N4
D6
N6
Tissue
Bracts
Flag_Leaf
Seed
Spike

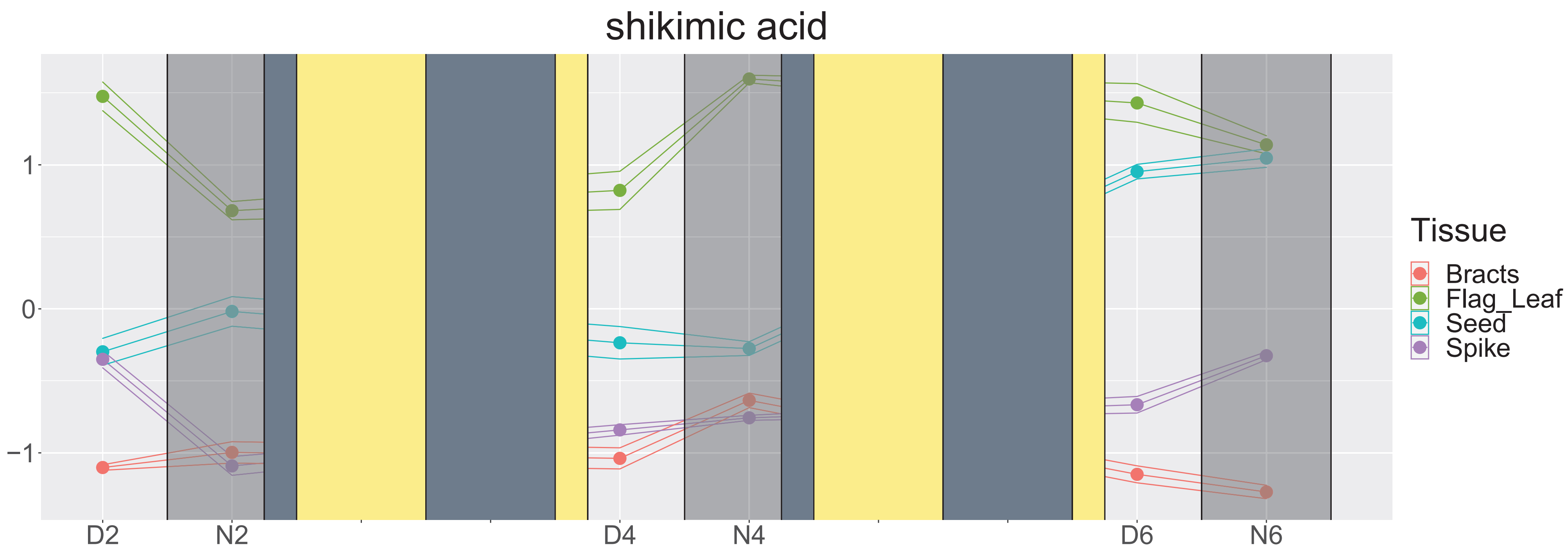

shikimic acid
1
0
−1
D2
N2
D4
N4
D6
N6
Tissue
Bracts
Flag_Leaf
Seed
Spike

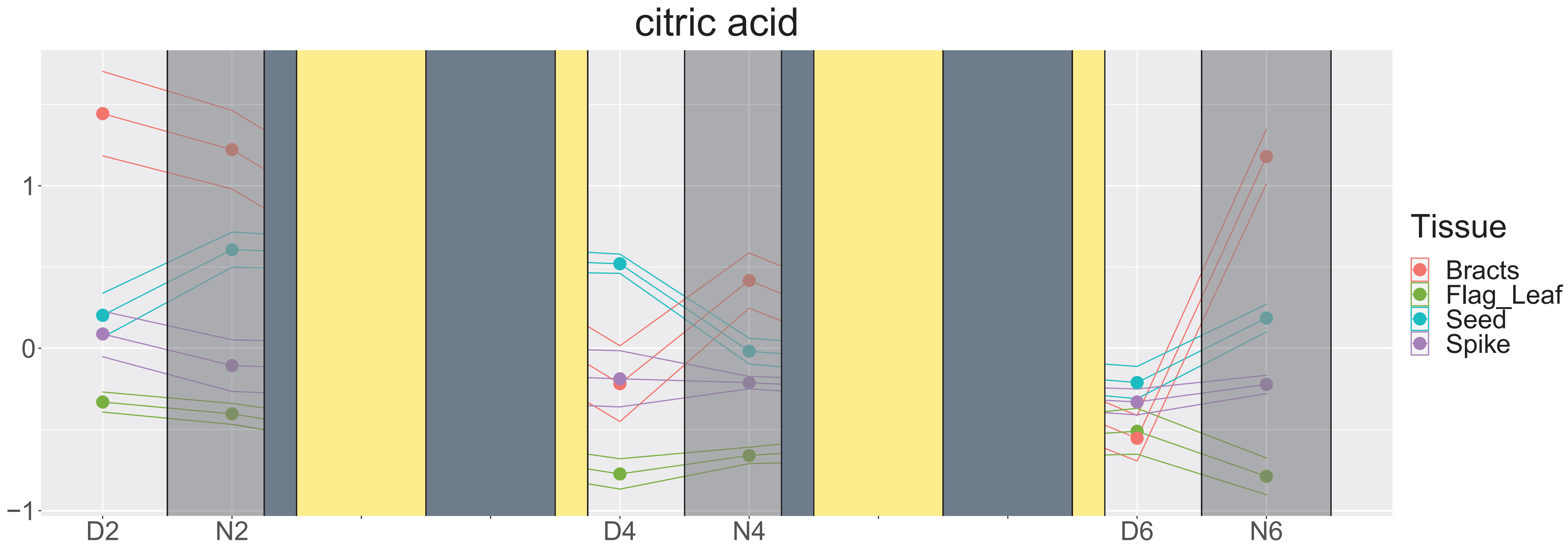

citric acid
1
0
−1
D2
N2
D4
N4
D6
N6
Tissue
Bracts
Flag_Leaf
Seed
Spike

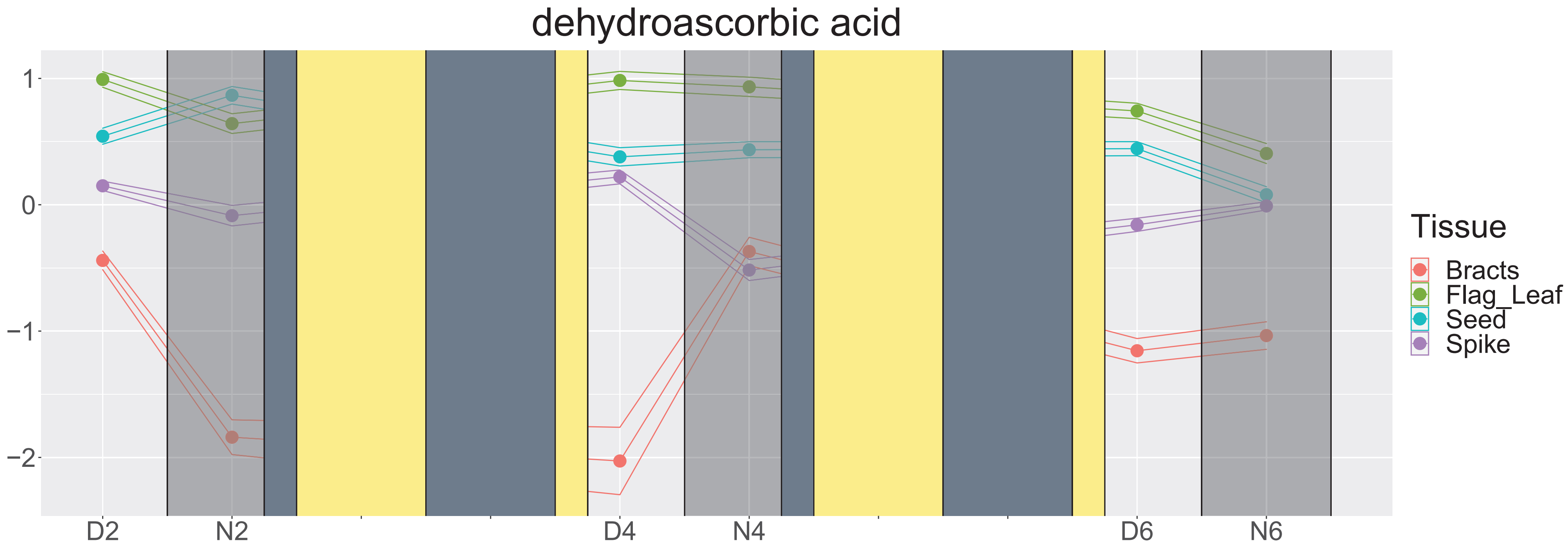

dehydroascorbic acid
1
0
−1
−2
D2
N2
D4
N4
D6
N6
Tissue
Bracts
Flag_Leaf
Seed
Spike

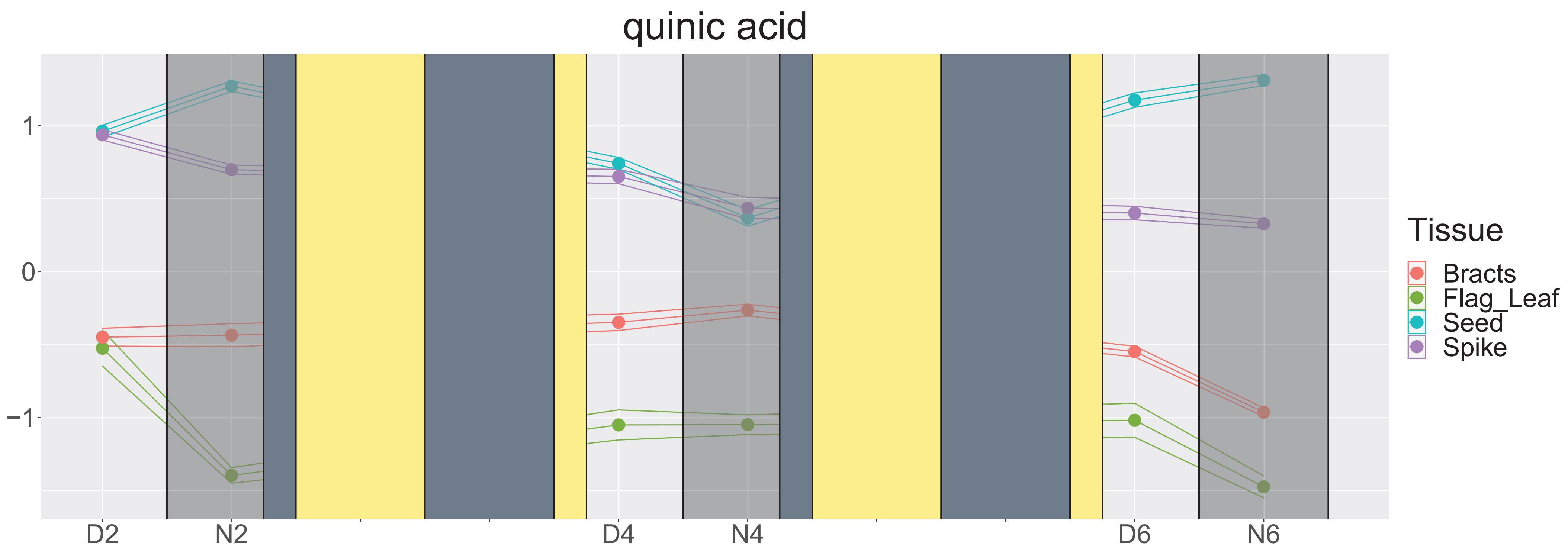

quinic acid
1
0
−1
D2
N2
D4
N4
D6
N6
Tissue
Bracts
Flag_Leaf
Seed
Spike

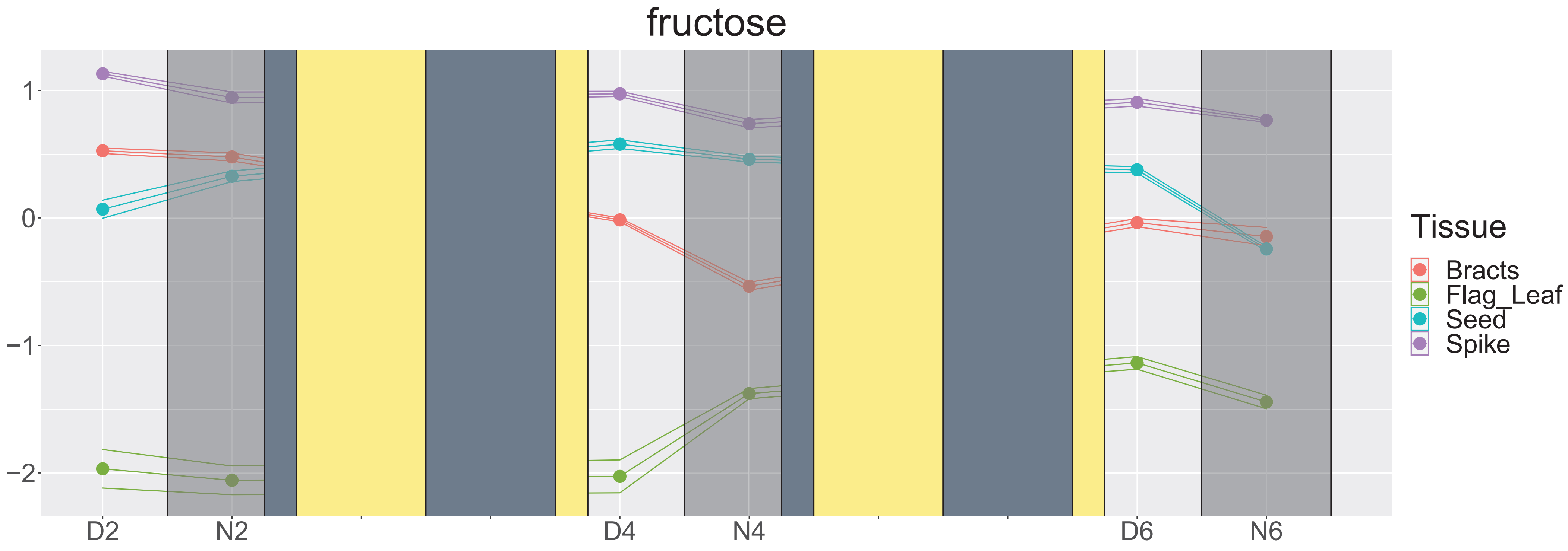

fructose
1
0
−1
−2
D2
N2
D4
N4
D6
N6
Tissue
Bracts
Flag_Leaf
Seed
Spike

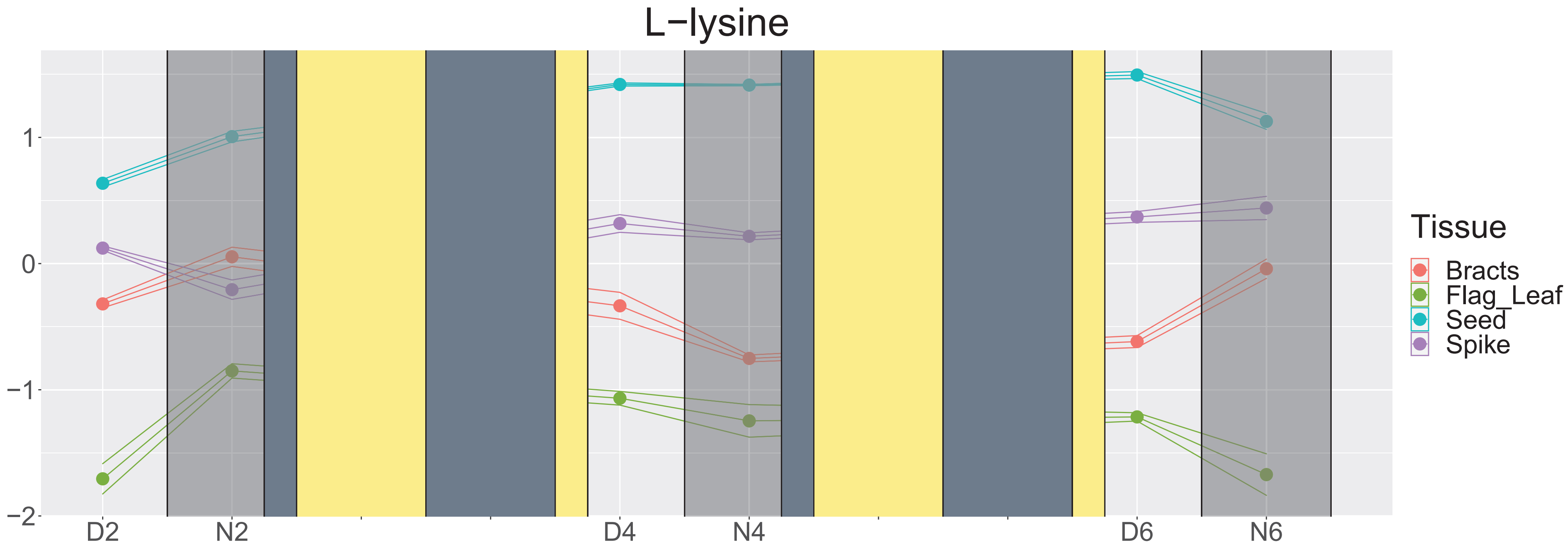

L−lysine
1
0
−1
−2
D2
N2
D4
N4
D6
N6
Tissue
Bracts
Flag_Leaf
Seed
Spike

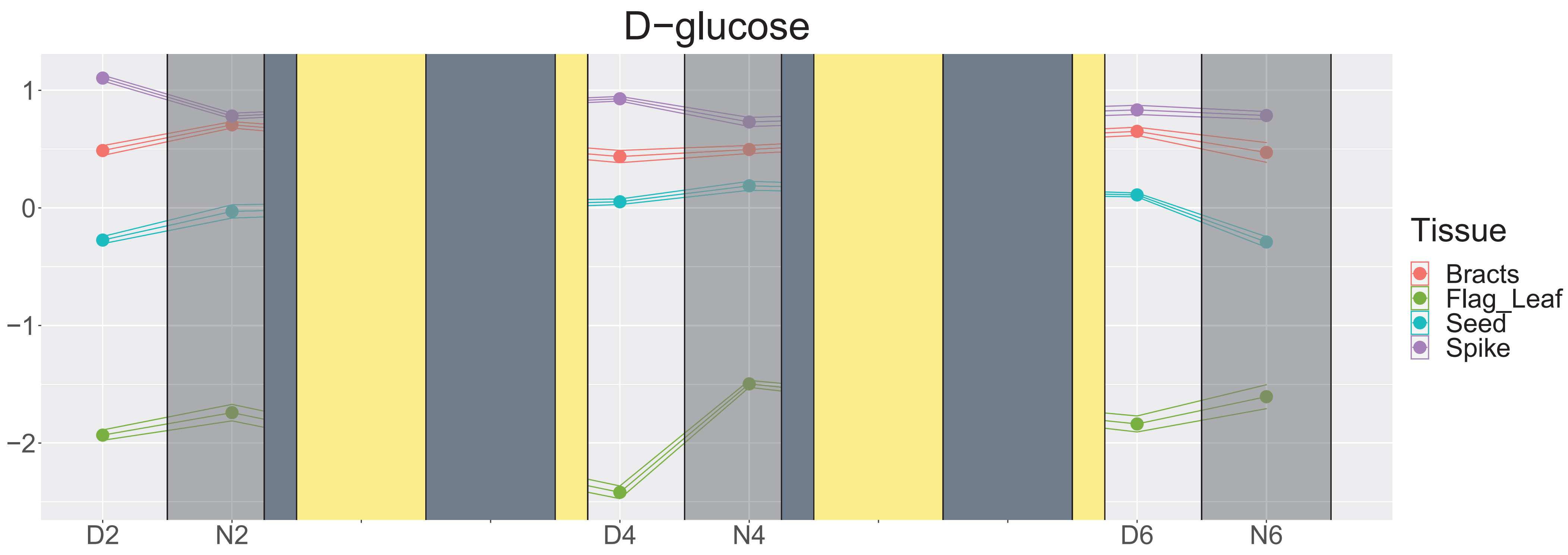

D−glucose
1
0
−1
−2
D2
N2
D4
N4
D6
N6
Tissue
Bracts
Flag_Leaf
Seed
Spike

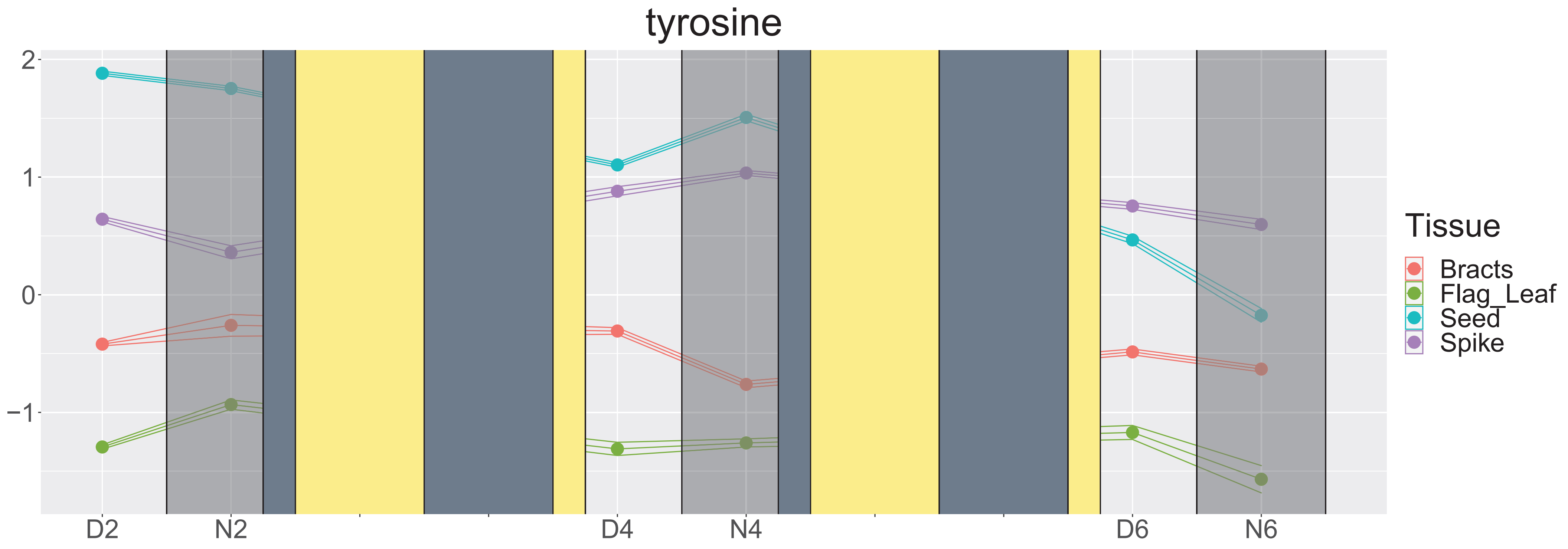

tyrosine
2
1
0
−1
D2
N2
D4
N4
D6
N6
Tissue
Bracts
Flag_Leaf
Seed
Spike

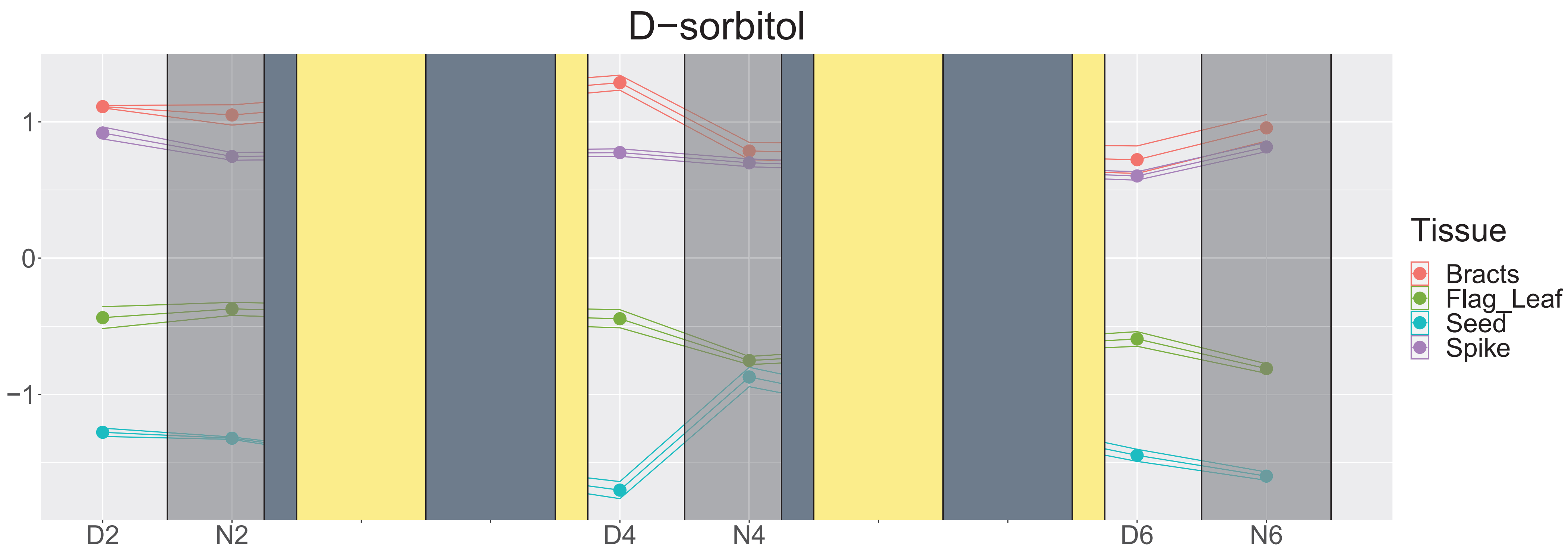

D−sorbitol
1
0
−1
D2
N2
D4
N4
D6
N6
Tissue
Bracts
Flag_Leaf
Seed
Spike

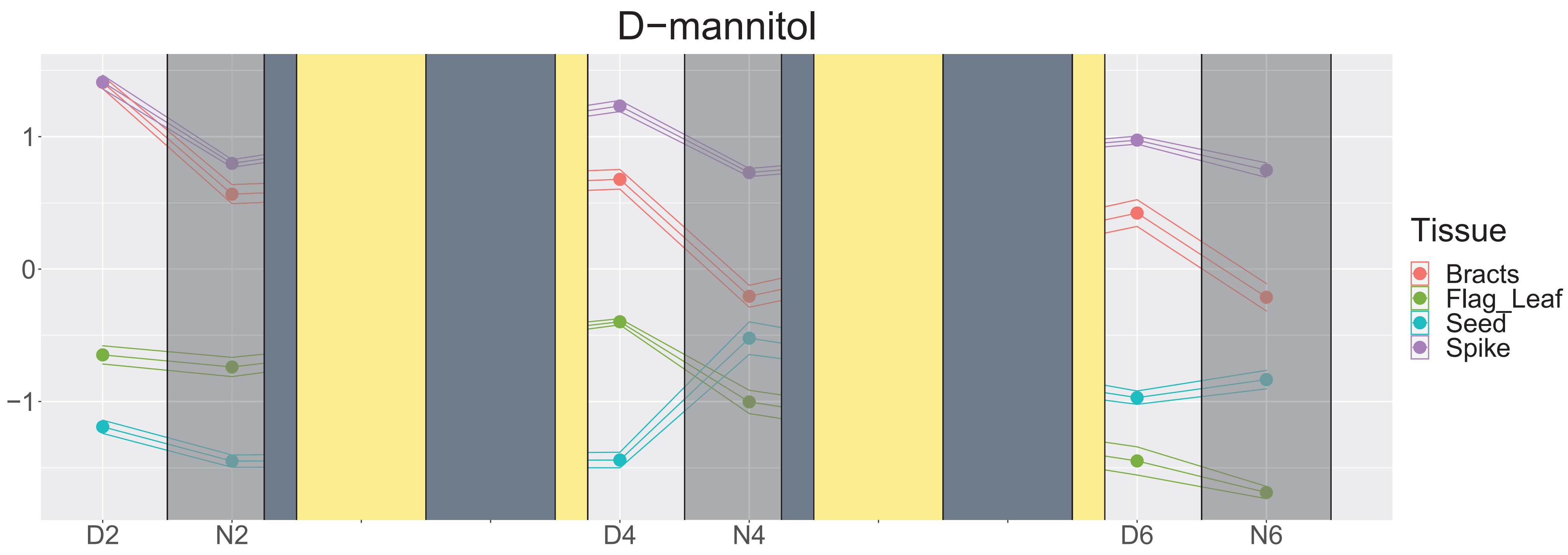

D−mannitol
1
0
−1
D2
N2
D4
N4
D6
N6
Tissue
Bracts
Flag_Leaf
Seed
Spike

isopropyl beta−D−1−thiogalactopyranoside

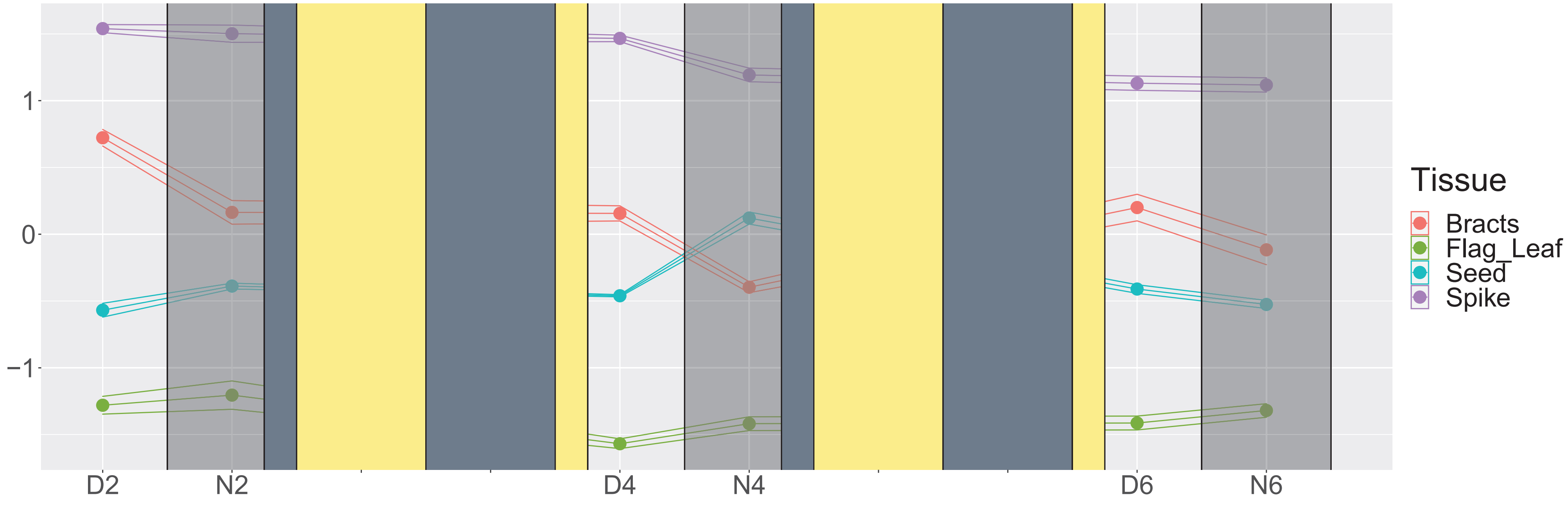

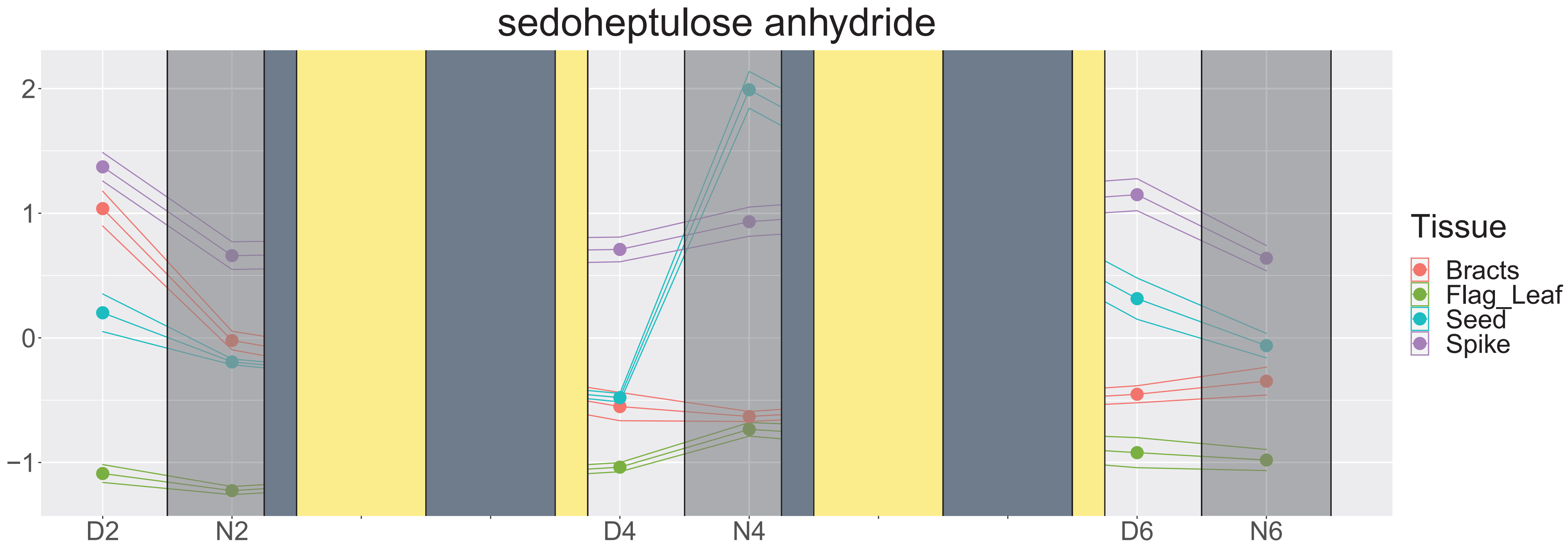

sedoheptulose anhydride
2
1
0
−1
D2
N2
D4
N4
D6
N6
Tissue
Bracts
Flag_Leaf
Seed
Spike

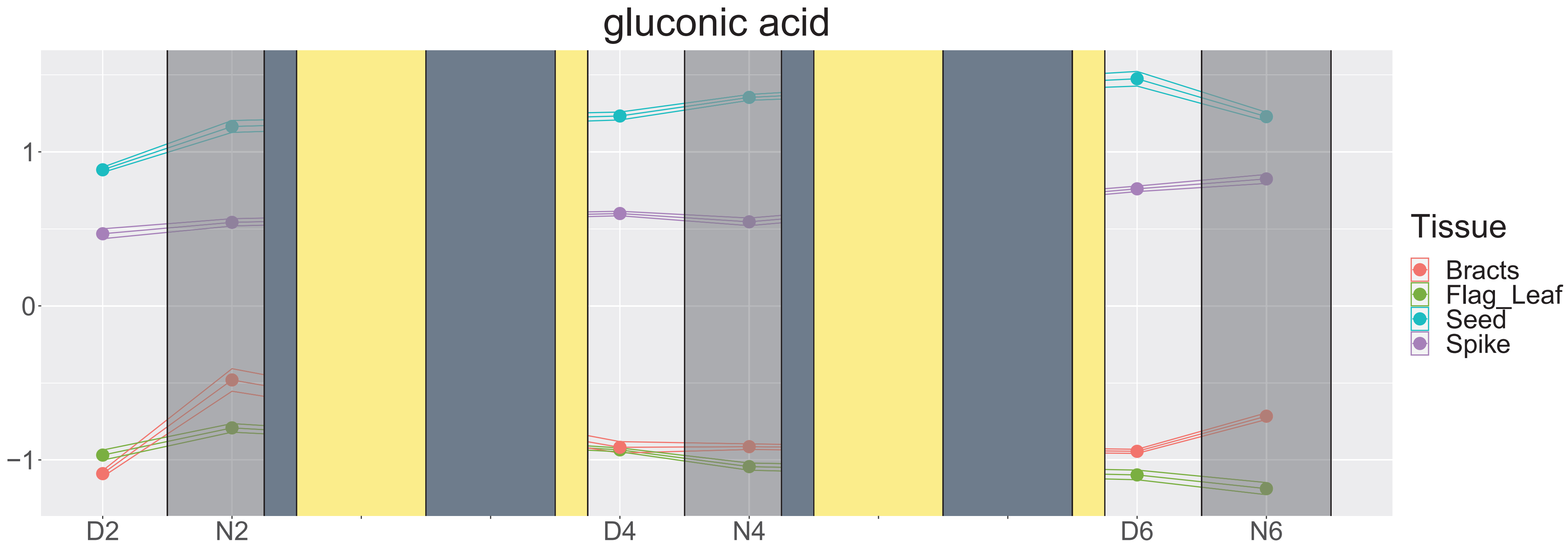
gluconic acid
1
0
−1
D2
N2
D4
N4
D6
N6
Tissue
Bracts
Flag_Leaf
Seed
Spike

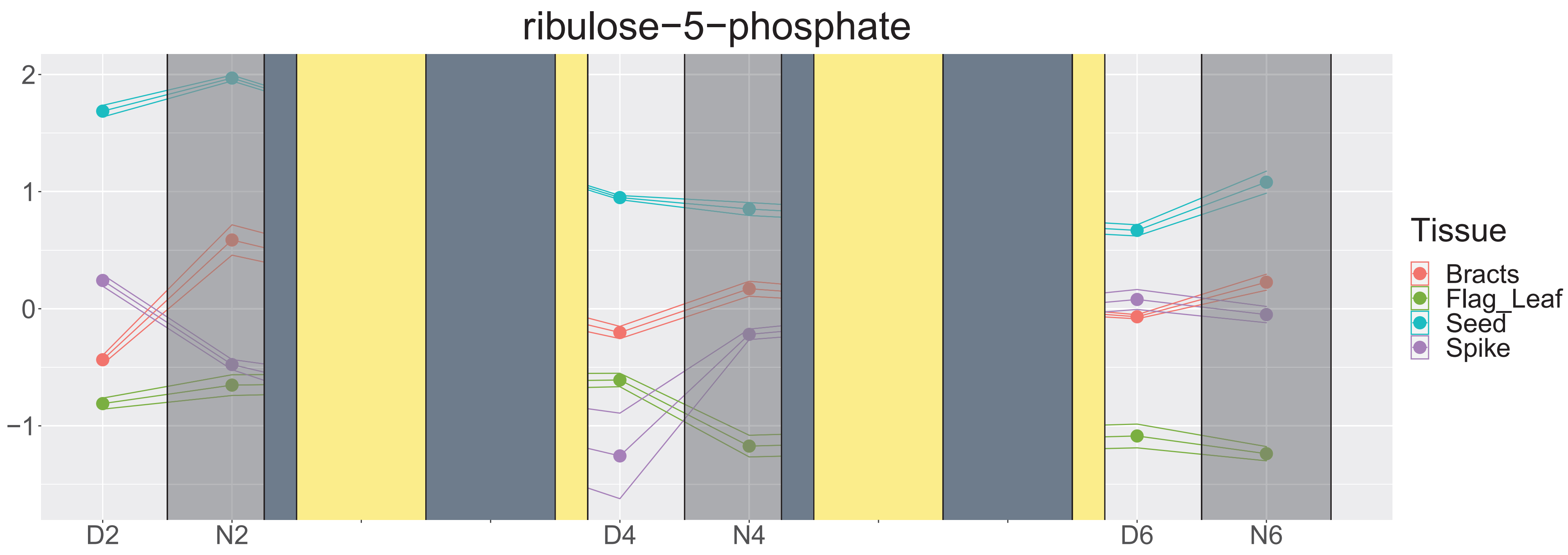
ribulose−5−phosphate
2
1
0
−1
D2
N2
D4
N4
D6
N6
Tissue
Bracts
Flag_Leaf
Seed
Spike

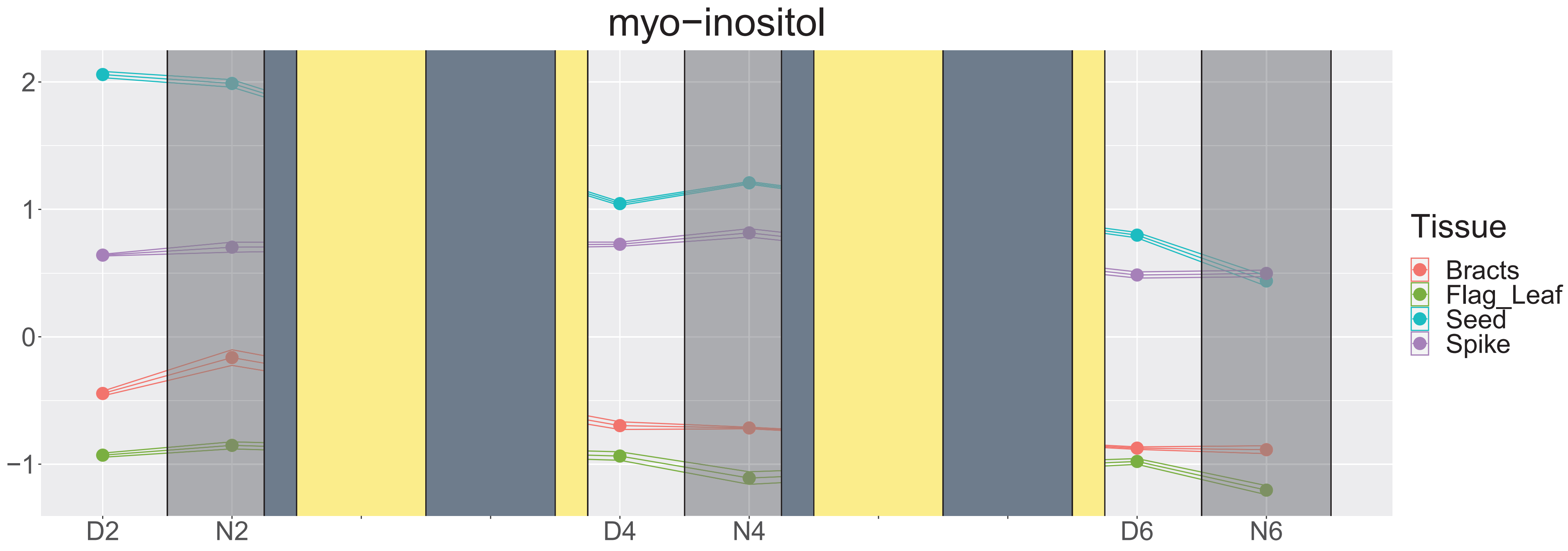
myo−inositol
2
1
0
−1
D2
N2
D4
N4
D6
N6
Tissue
Bracts
Flag_Leaf
Seed
Spike

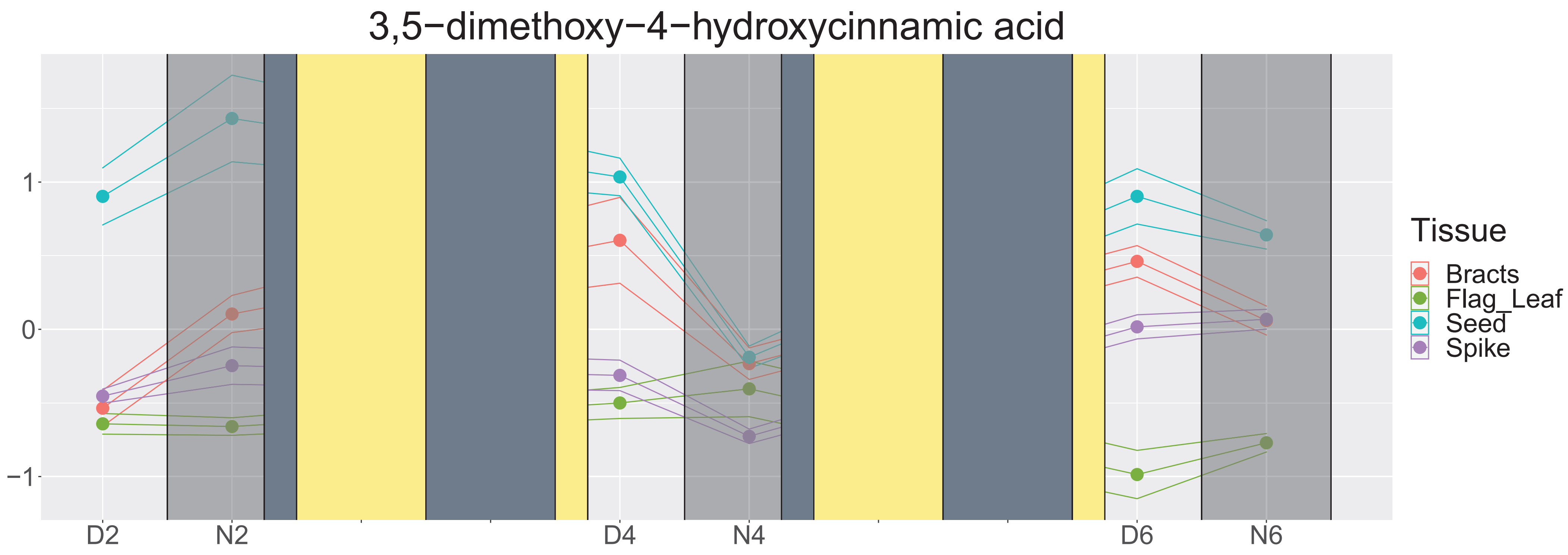

3,5−dimethoxy−4−hydroxycinnamic acid
1
0
−1
D2
N2
D4
N4
D6
N6
Tissue
Bracts
Flag_Leaf
Seed
Spike

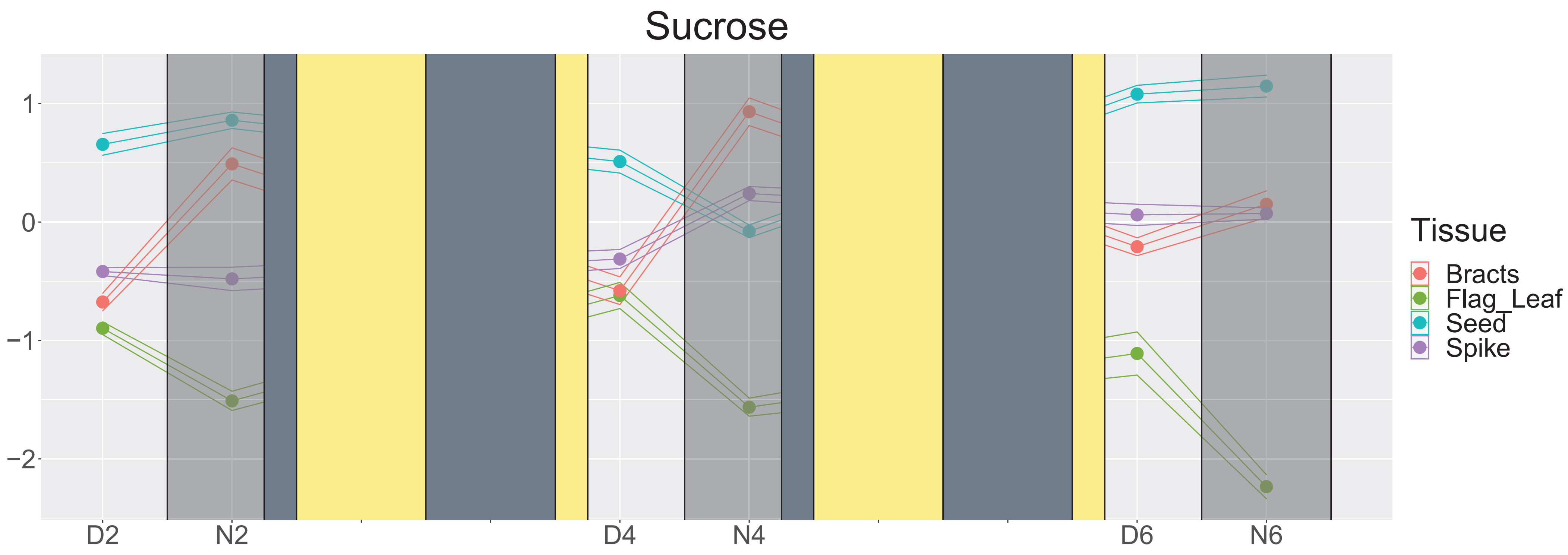

Sucrose
1
0
−1
−2
D2
N2
D4
N4
D6
N6
Tissue
Bracts
Flag_Leaf
Seed
Spike

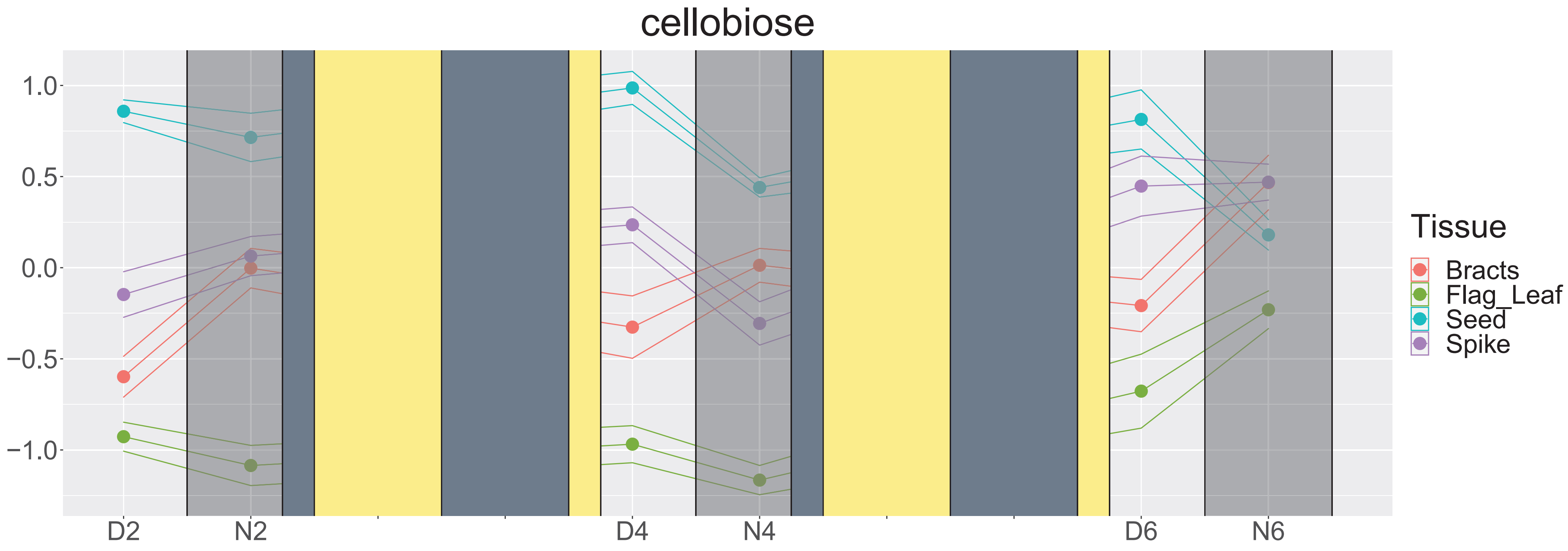

cellobiose
1.0
0.5
0.0
−0.5
−1.0
D2
N2
D4
N4
D6
N6
Tissue
Bracts
Flag_Leaf
Seed
Spike

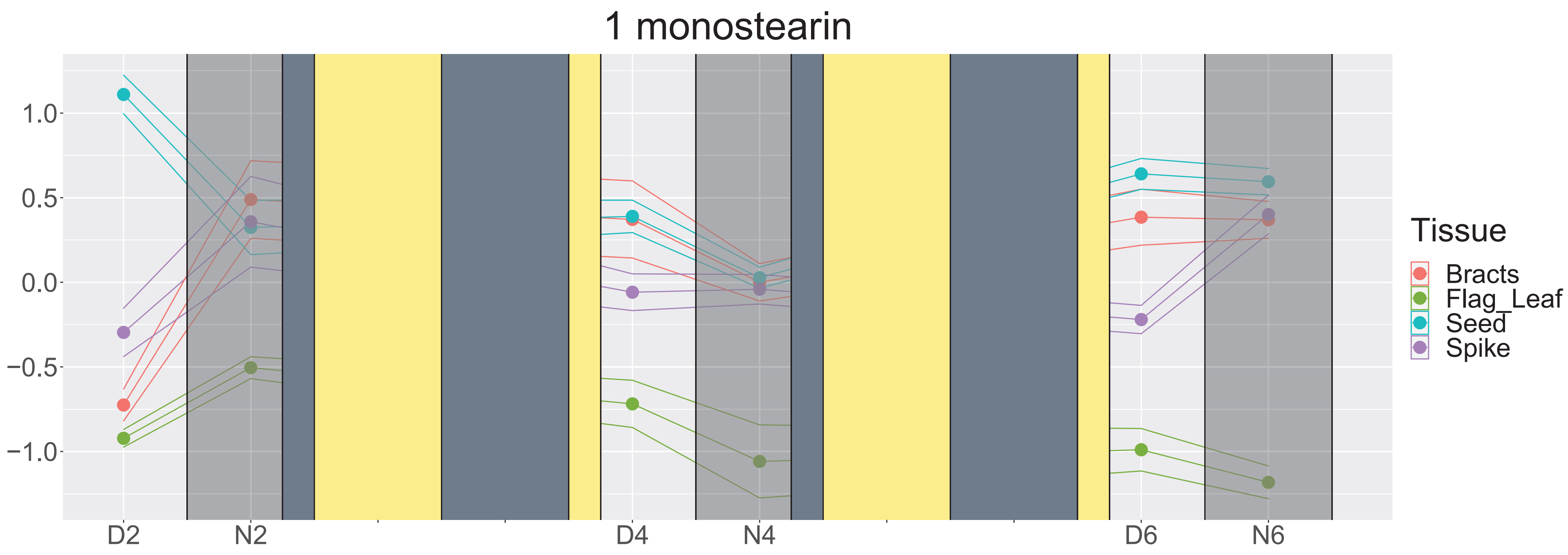
1 monostearin
1.0
0.5
0.0
−0.5
−1.0
D2
N2
D4
N4
D6
N6
Tissue
Bracts
Flag_Leaf
Seed
Spike

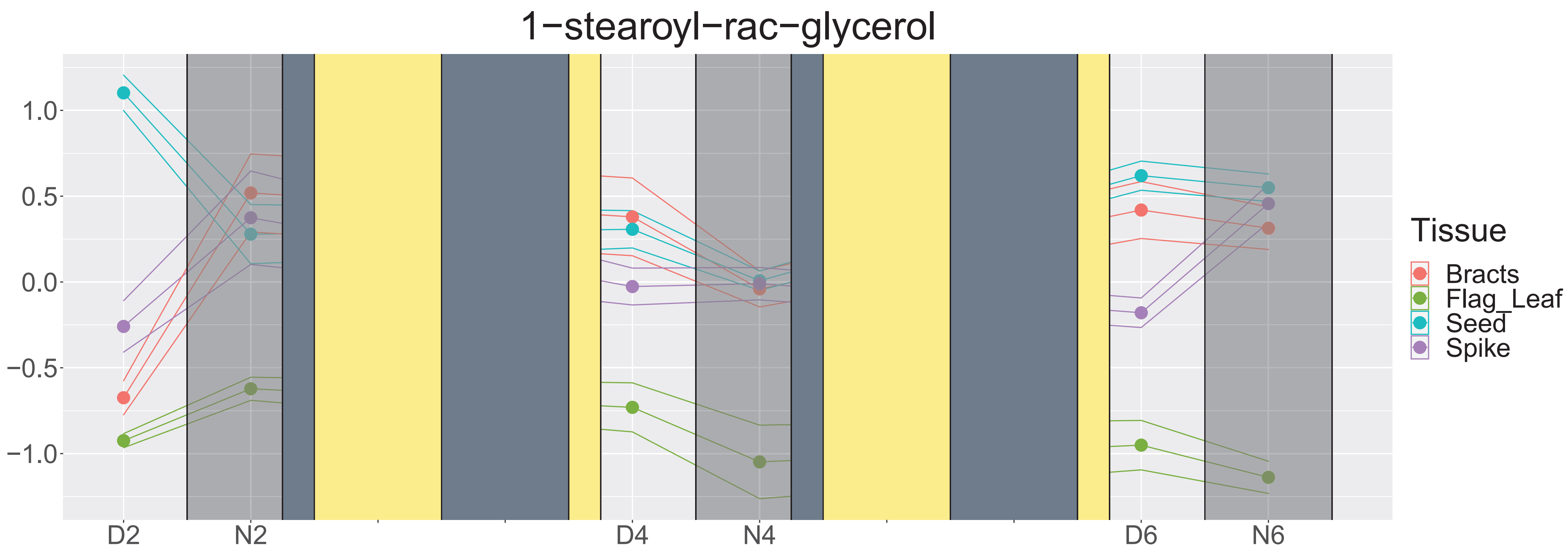
1−stearoyl−rac−glycerol
1.0
0.5
0.0
−0.5
−1.0
D2
N2
D4
N4
D6
N6
Tissue
Bracts
Flag_Leaf
Seed
Spike

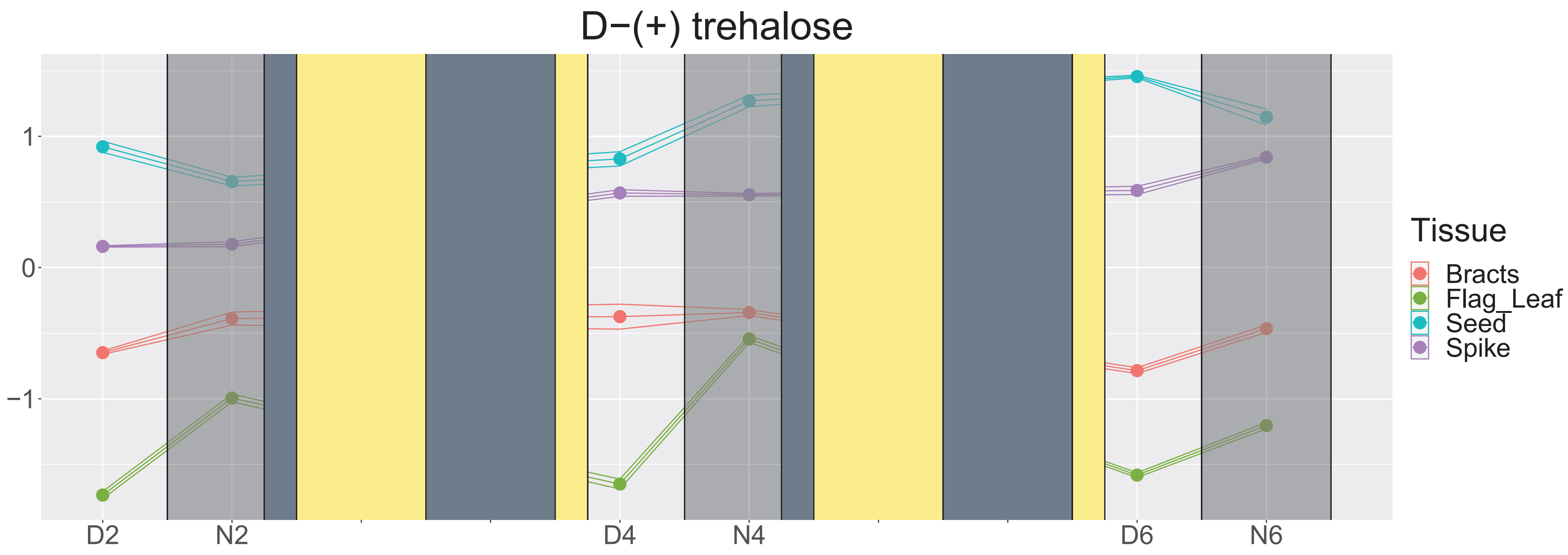

D−(+) trehalose
1
0
−1
D2
N2
D4
N4
D6
N6
Tissue
Bracts
Flag_Leaf
Seed
Spike

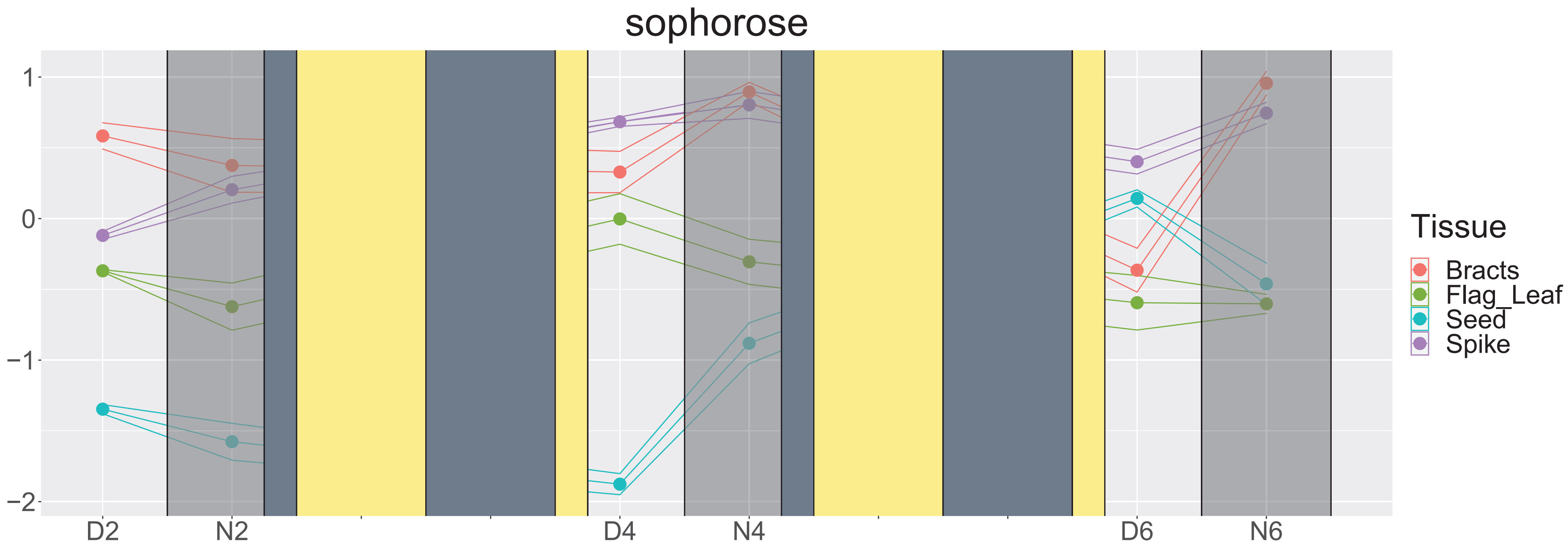

sophorose
1
0
−1
−2
D2
N2
D4
N4
D6
N6
Tissue
Bracts
Flag_Leaf
Seed
Spike

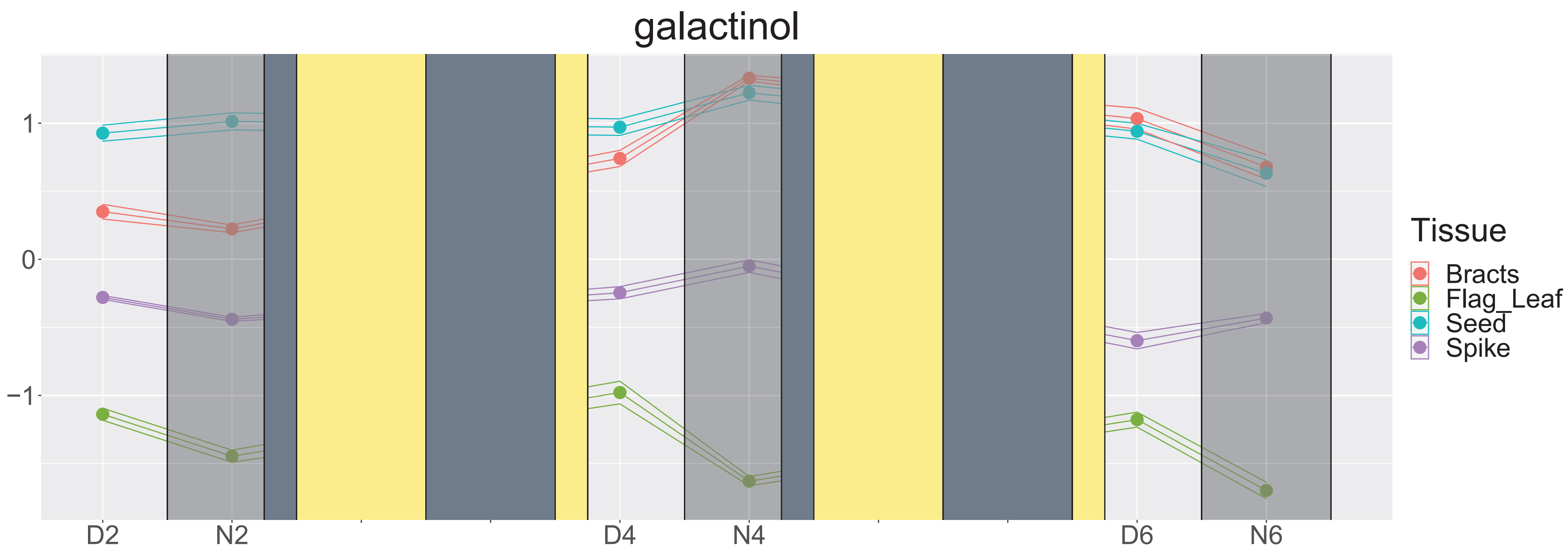
galactinol
1
0
−1
D2
N2
D4
N4
D6
N6
Tissue
Bracts
Flag_Leaf
Seed
Spike

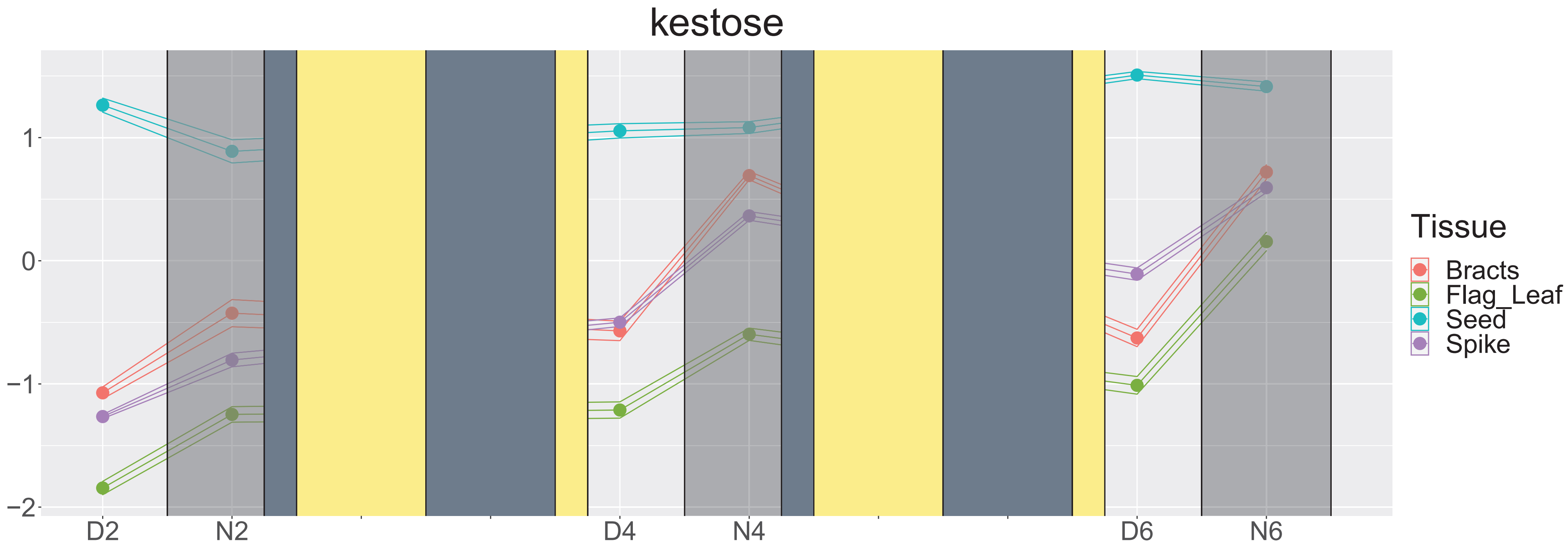
kestose
1
0
−1
−2
D2
N2
D4
N4
D6
N6
Tissue
Bracts
Flag_Leaf
Seed
Spike
