# Supplementary material for: Tissue- and time-dependent metabolite profiles during early grain development under normal and high night-time temperature conditions
Source: BMC Plant Biol. 2024 Jun 18;24:568. doi: 10.1186/s12870-024-05190-6 (PMC11184705; doi:10.1186/s12870-024-05190-6)
Supplement: Supplementary file 3 — Supplementary Material 3. [file 12870_2024_5190_MOESM3_ESM.pdf]

Supplemental Fig. S4. Time courses of the abundance of all metabolites under control and high night temperature (HNT) conditions. The metabolite abundances in the control condition are identical to Supplemental Fig. S2. The Y axis represents relative metabolic abundance. X axis represents time point, where D or N represent day or night time sampling and 2, 4, and 6 represent the days after fertilization (DAF) of sampling. Solid rectangles were overlaid over the chart to represent the days between sample collection, with dull yellow representing day, and dull blue representing night. Data is represented as mean values at each time point, as well as flanking lines representing one +- standard error, from up to five biological replicates. Each facet represents a tissue. Color within the facet represents treatment. HNT, high night temperature. Values in each metabolite under all conditions, including all treatments and tissue types, were log2 transformed, and Z transformed with a mean of zero and a standard deviation of 1.

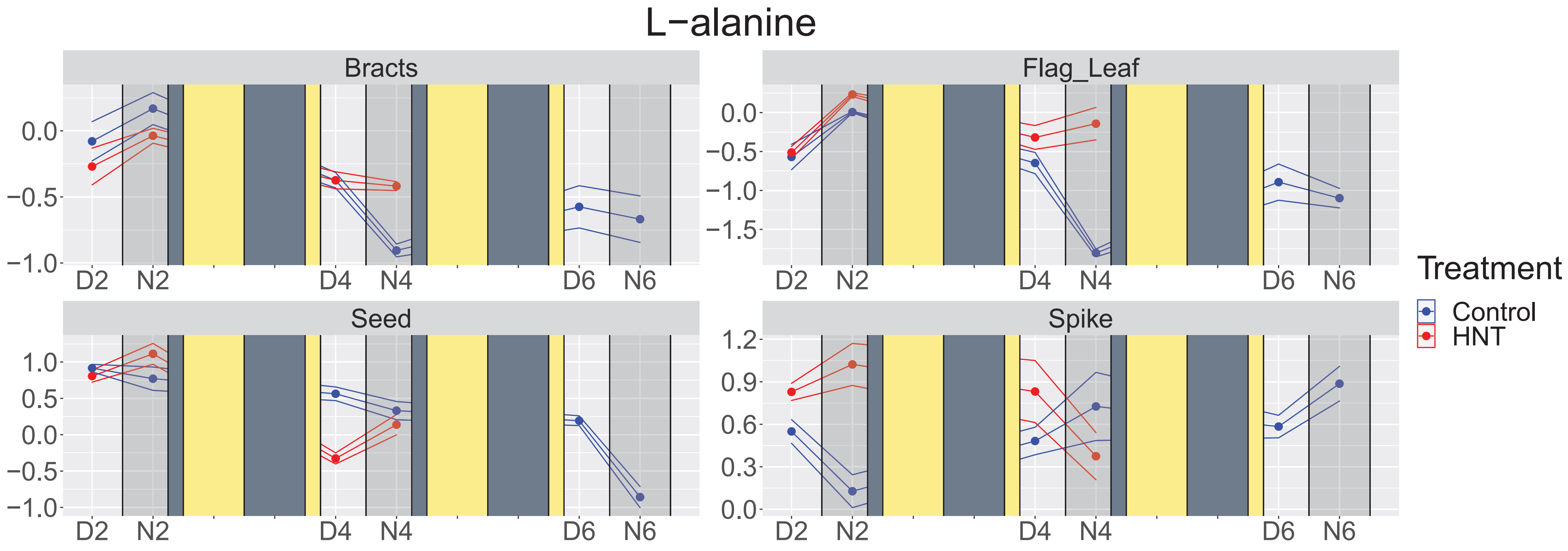
L−alanine
Bracts
Flag_Leaf
Seed
Spike
D2
N2
D4
N4
D6
N6
Treatment
Control
HNT

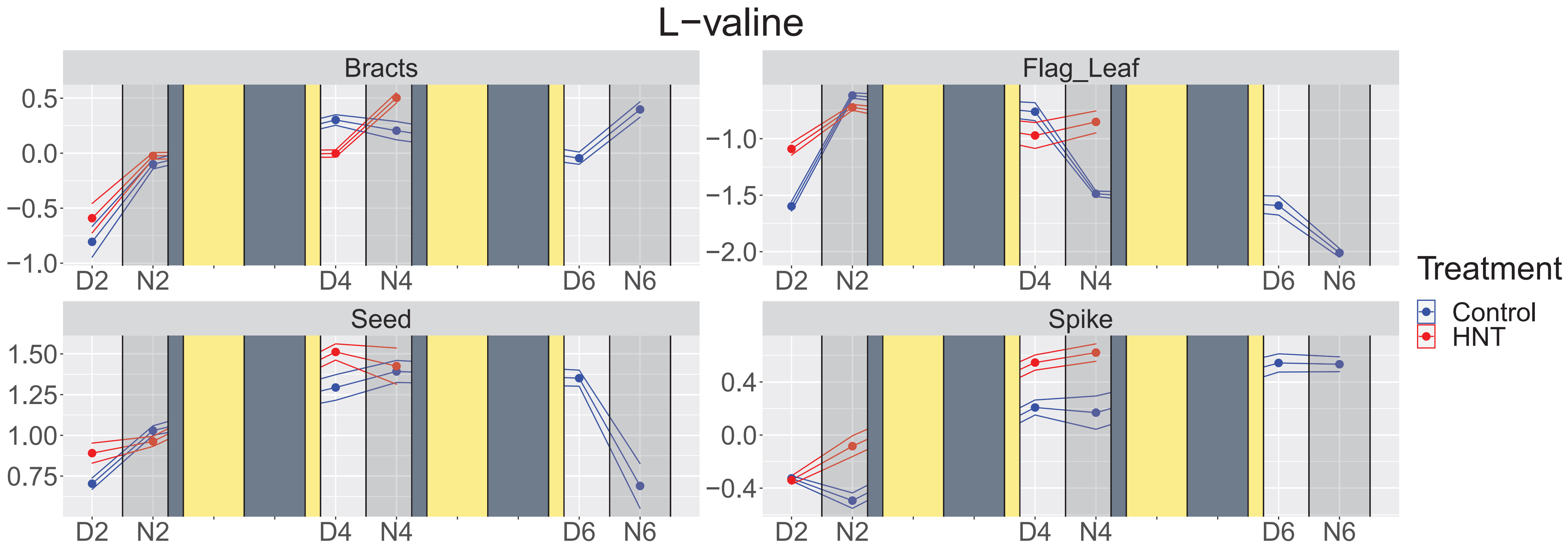

L−valine
Bracts
Flag_Leaf
Seed
Spike
0.5
0.0
−0.5
−1.0
−1.5
−2.0
1.50
1.25
1.00
0.75
0.4
−0.4
D2
N2
D4
N4
D6
N6
Treatment
Control
HNT

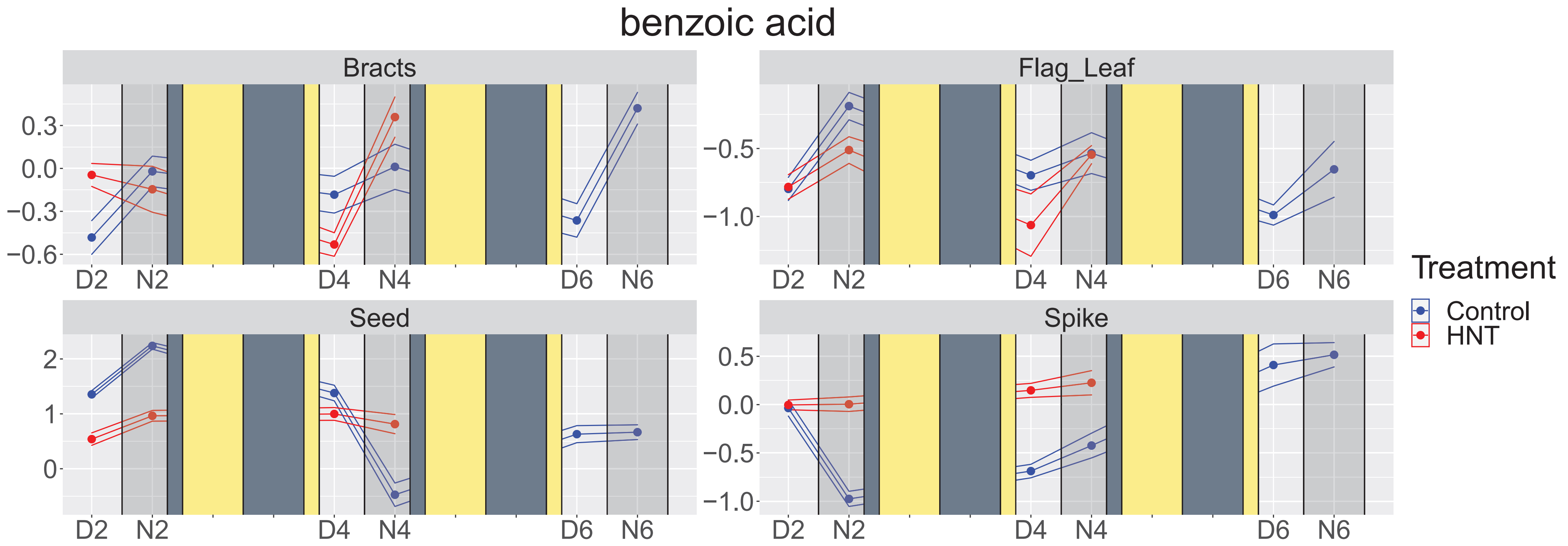
benzoic acid
Bracts
Flag_Leaf
Seed
Spike
0.3
0.0
−0.3
−0.6
−0.5
−1.0
2
1
0
0.5
0.0
−0.5
−1.0
D2
N2
D4
N4
D6
N6
Treatment
Control
HNT

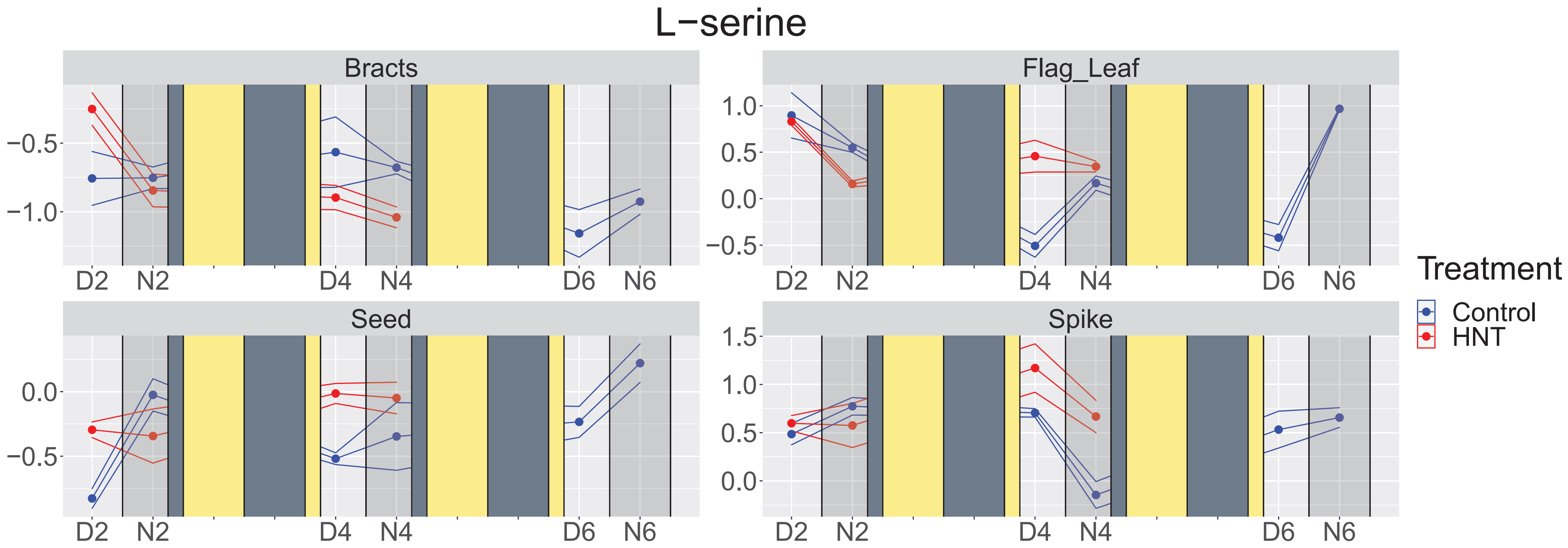
L−serine
Bracts
Flag_Leaf
Seed
Spike
−0.5
−1.0
1.0
0.5
0.0
−0.5
0.0
−0.5
1.5
1.0
0.5
0.0
D2
N2
D4
N4
D6
N6
Treatment
Control
HNT

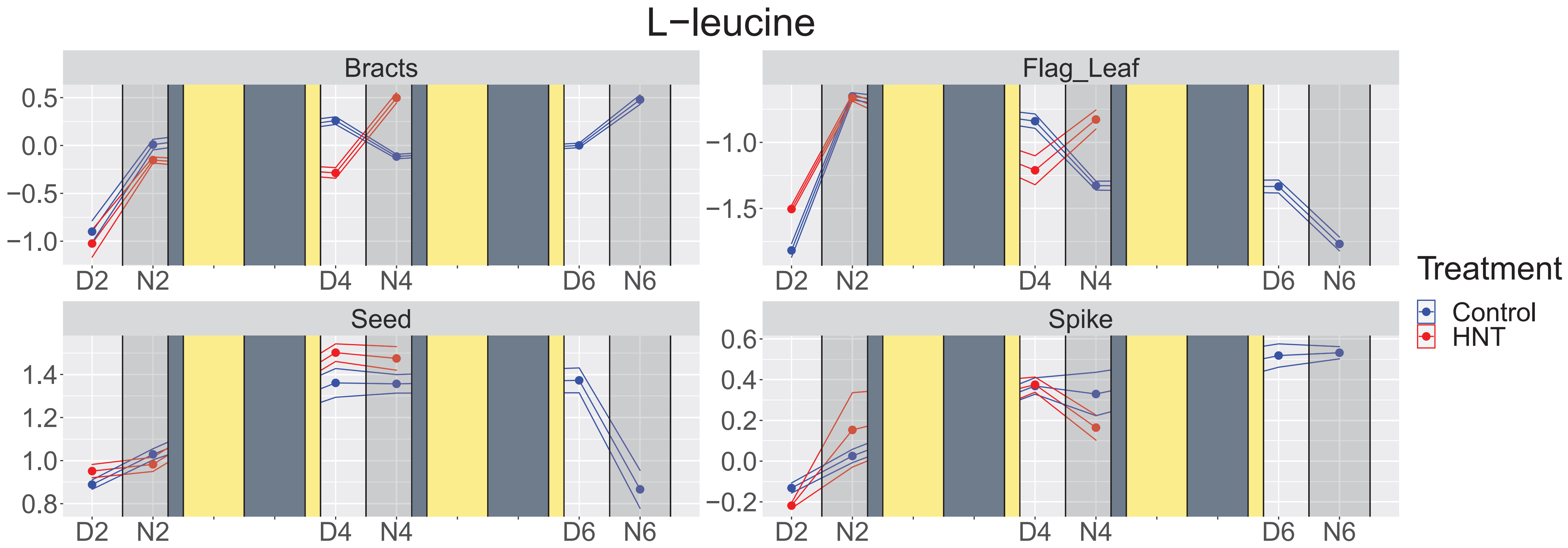

L−leucine
Bracts
Flag_Leaf
Seed
Spike
0.5
0.0
−0.5
−1.0
−1.5
1.4
1.2
1.0
0.8
0.6
0.4
0.2
−0.2
D2
N2
D4
N4
D6
N6
Treatment
Control
HNT

phosphoric acid

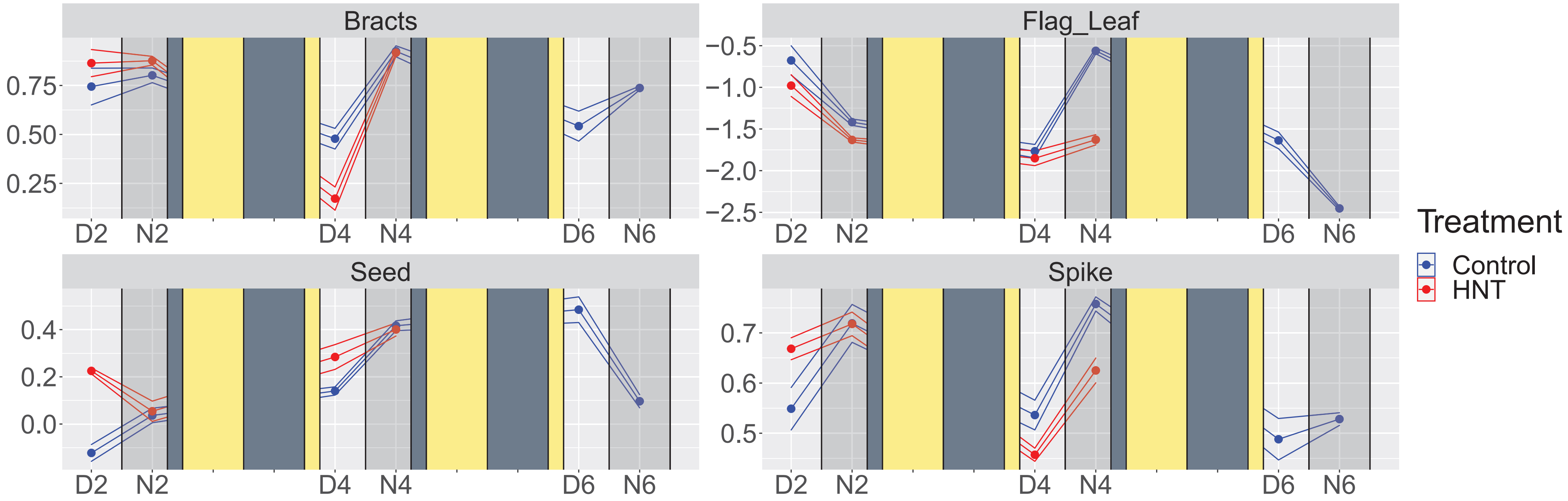

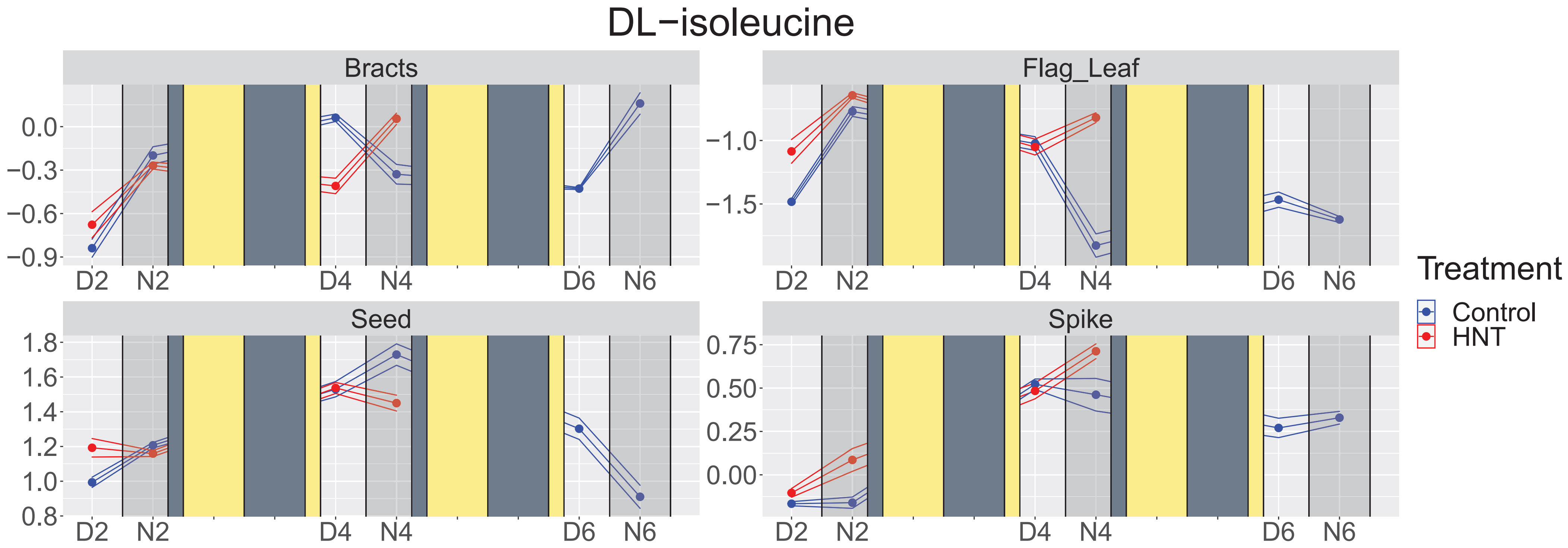

DL−isoleucine
Bracts
Flag_Leaf
Seed
Spike
0.0
−0.3
−0.6
−0.9
−1.0
−1.5
1.8
1.6
1.4
1.2
1.0
0.8
0.75
0.50
0.25
0.00
D2
N2
D4
N4
D6
N6
Treatment
Control
HNT

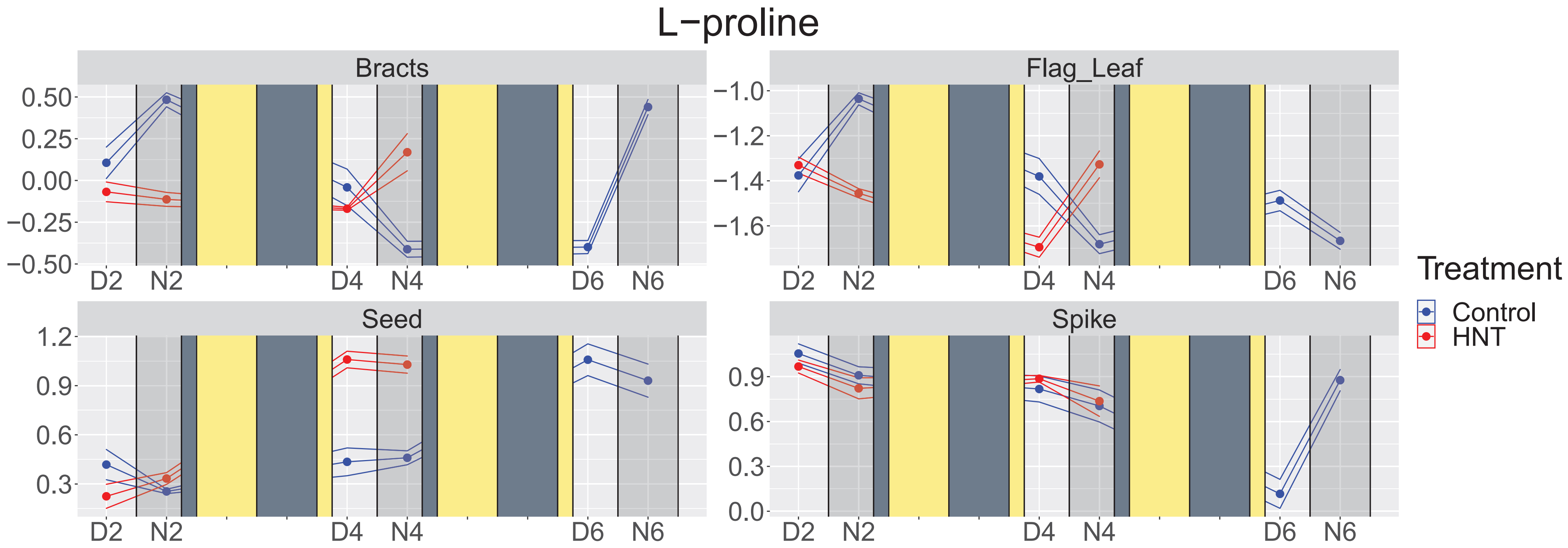

L−proline
Bracts
Flag_Leaf
Seed
Spike
0.50
0.25
0.00
−0.25
−0.50
−1.0
−1.2
−1.4
−1.6
1.2
0.9
0.6
0.3
0.0
D2
N2
D4
N4
D6
N6
Treatment
Control
HNT

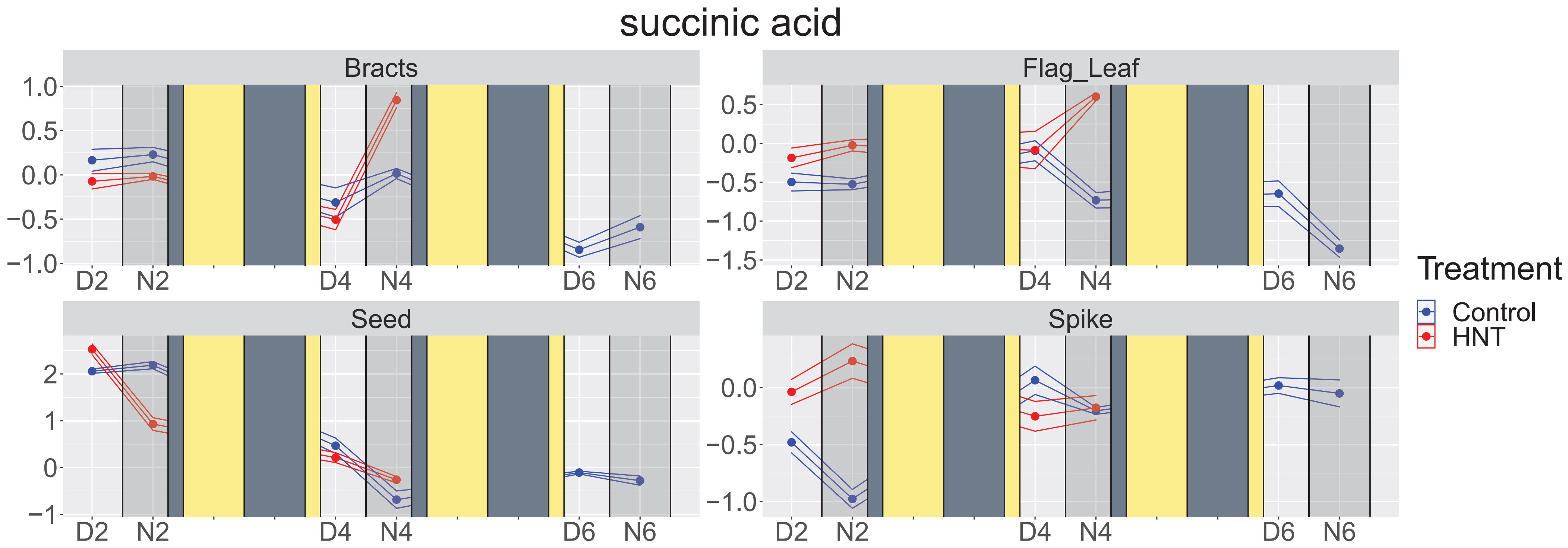

succinic acid
Bracts
Flag_Leaf
Seed
Spike
1.0
0.5
0.0
−0.5
−1.0
−1.5
2
1
0
−1
D2
N2
D4
N4
D6
N6
Treatment
Control
HNT

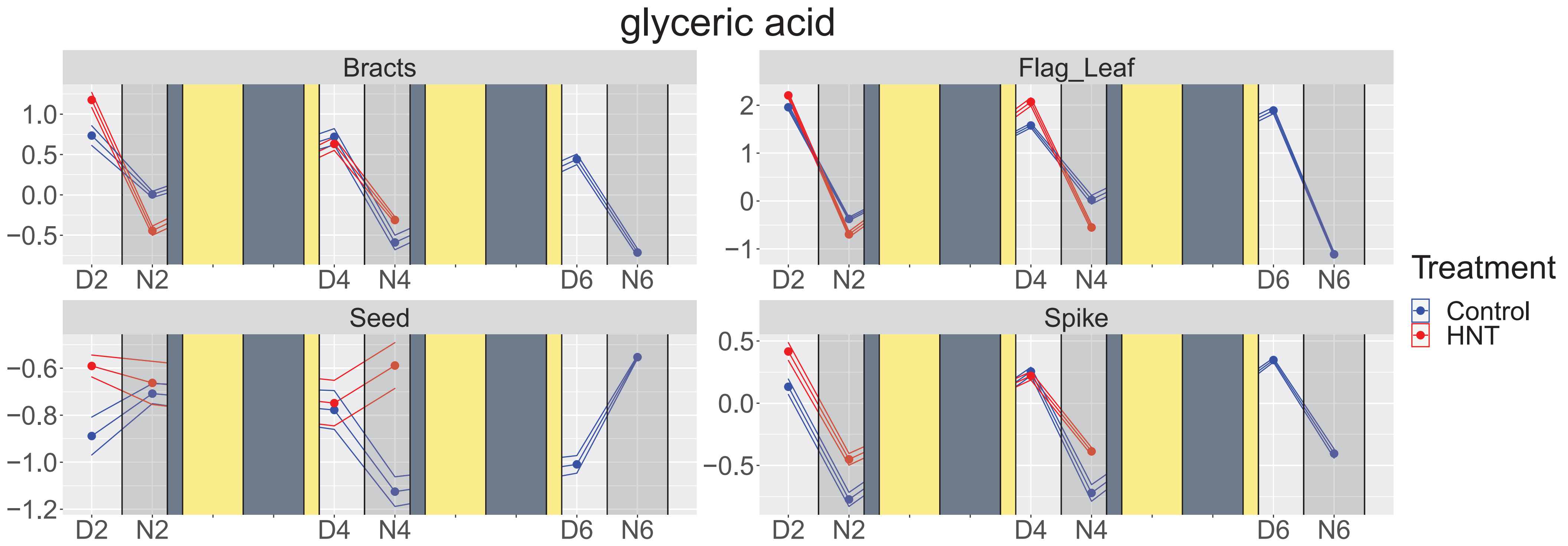
glyceric acid
Bracts
Flag_Leaf
Seed
Spike
1.0
0.5
0.0
−0.5
2
1
0
−1
−0.6
−0.8
−1.0
−1.2
0.5
0.0
−0.5
D2
N2
D4
N4
D6
N6
Treatment
Control
HNT

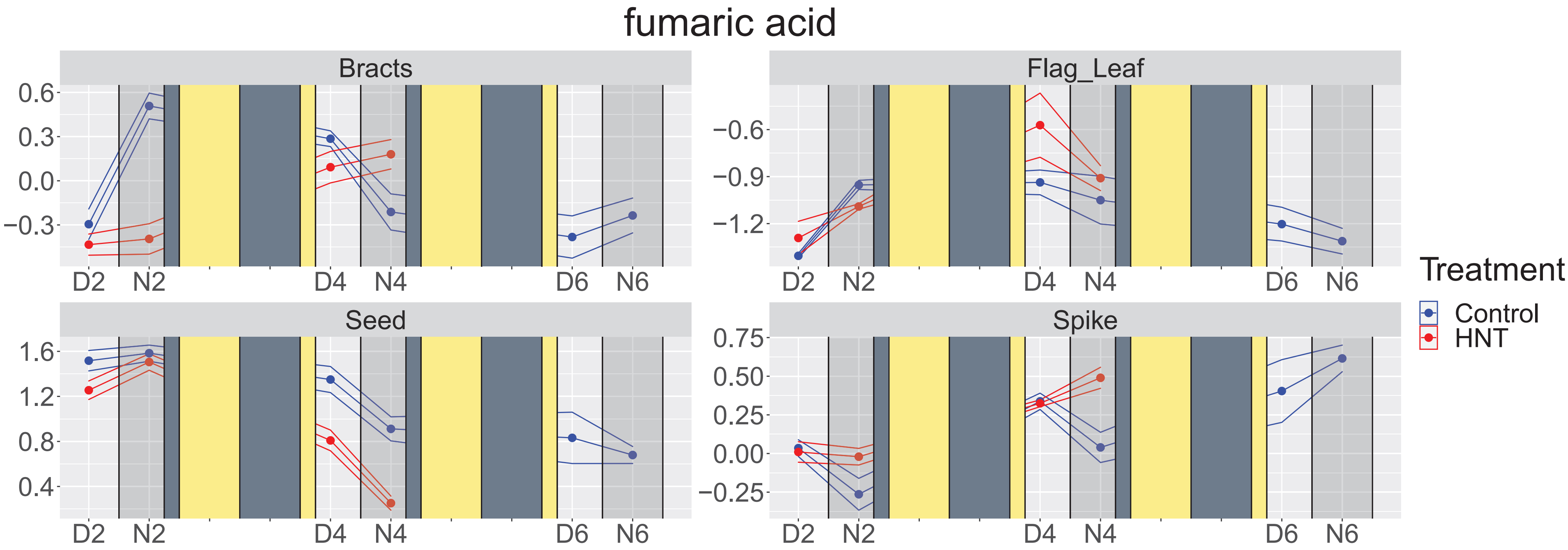

fumaric acid
Bracts
Flag_Leaf
Seed
Spike
D2
N2
D4
N4
D6
N6
Treatment
Control
HNT

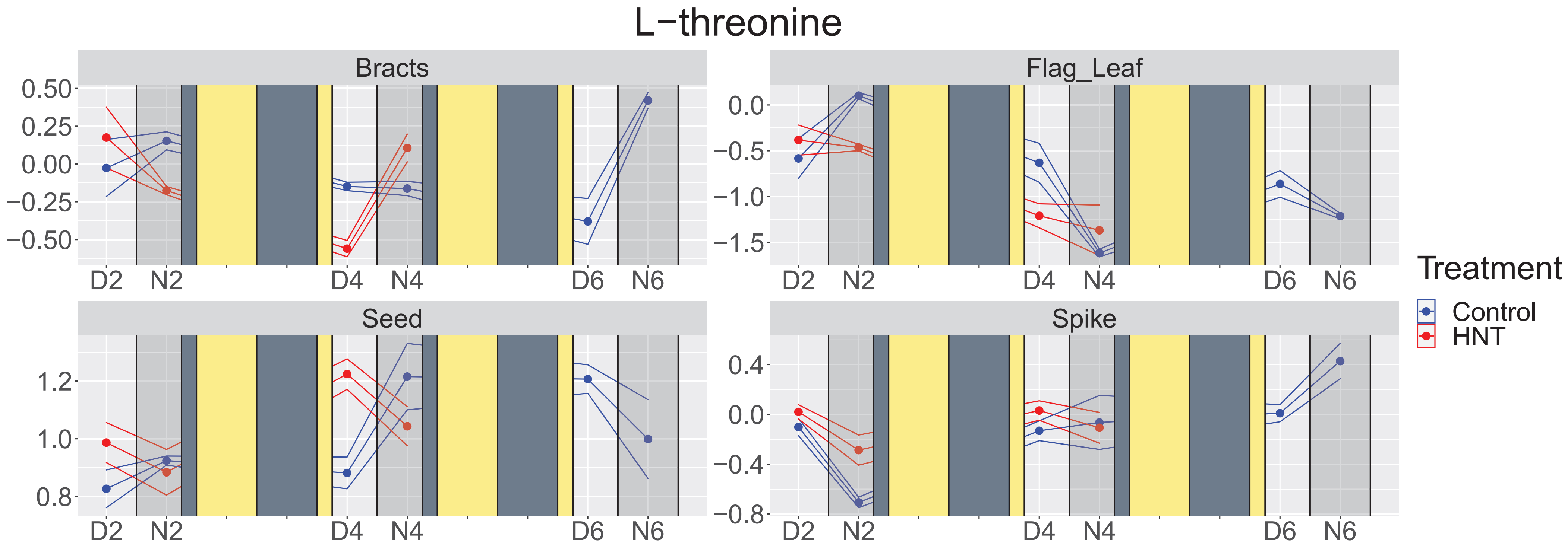
L−threonine
Bracts
Flag_Leaf
Seed
Spike
0.50
0.25
0.00
−0.25
−0.50
0.0
−0.5
−1.0
−1.5
1.2
1.0
0.8
0.4
0.0
−0.4
−0.8
D2 N2 D4 N4 D6 N6
Treatment
Control
HNT

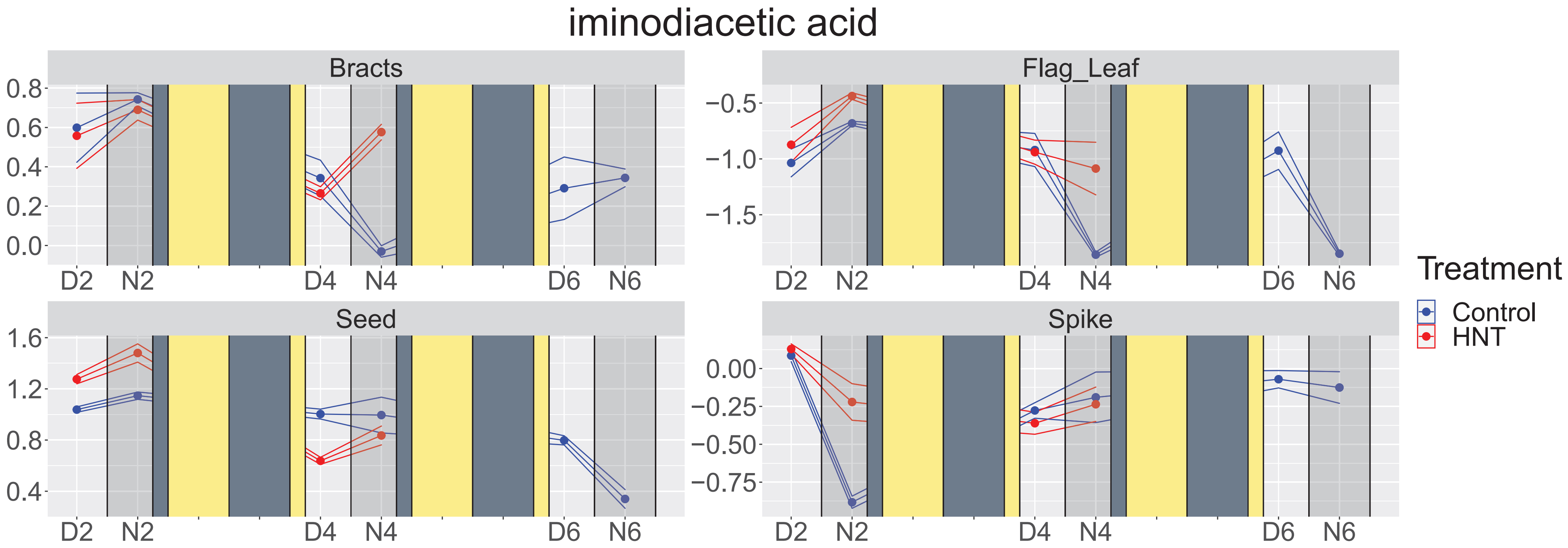

iminodiacetic acid
Bracts
Flag_Leaf
Seed
Spike
0.8
0.6
0.4
0.2
0.0
−0.5
−1.0
−1.5
1.6
1.2
0.8
0.4
0.00
−0.25
−0.50
−0.75
D2 N2 D4 N4 D6 N6
Treatment
Control
HNT

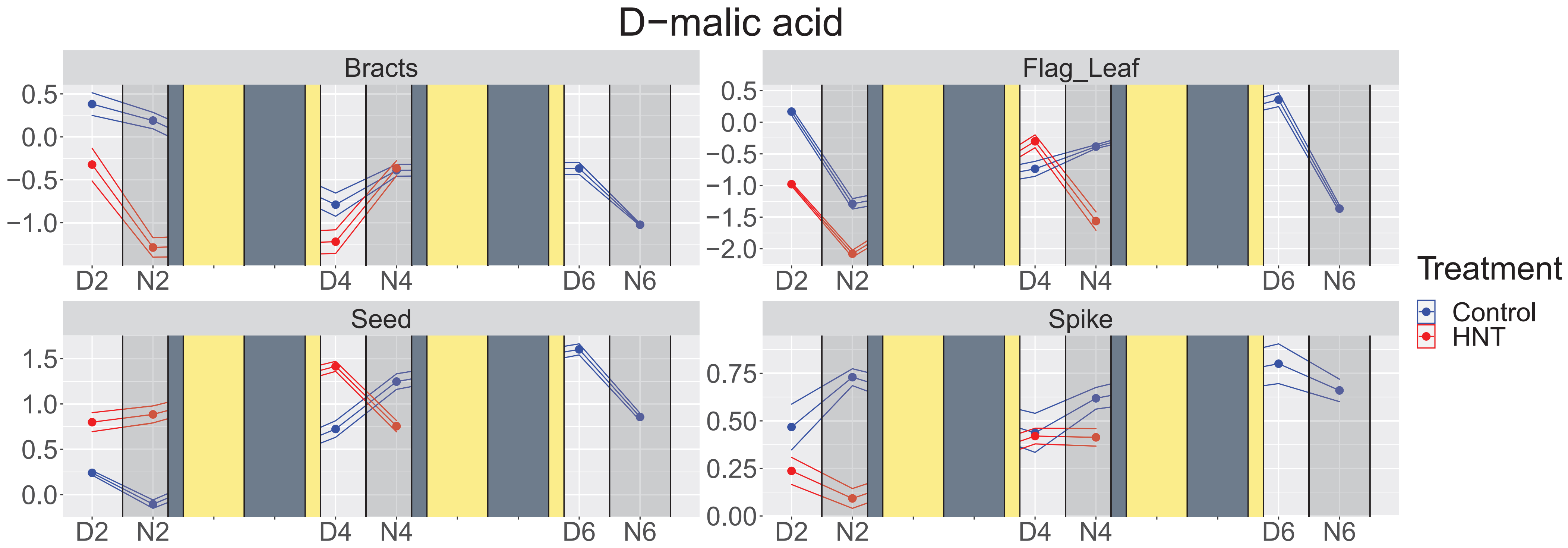

D−malic acid
Bracts
Flag_Leaf
Seed
Spike
D2
N2
D4
N4
D6
N6
Treatment
Control
HNT

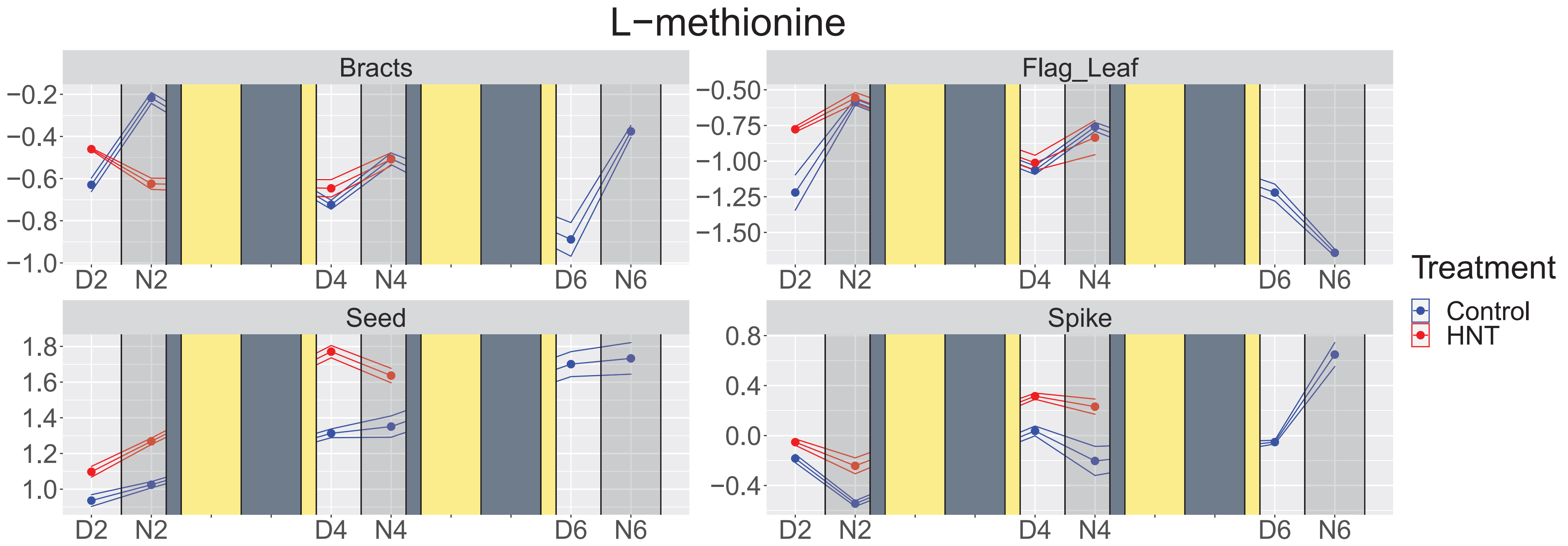

L−methionine
Bracts
Flag_Leaf
Seed
Spike
D2
N2
D4
N4
D6
N6
Treatment
Control
HNT

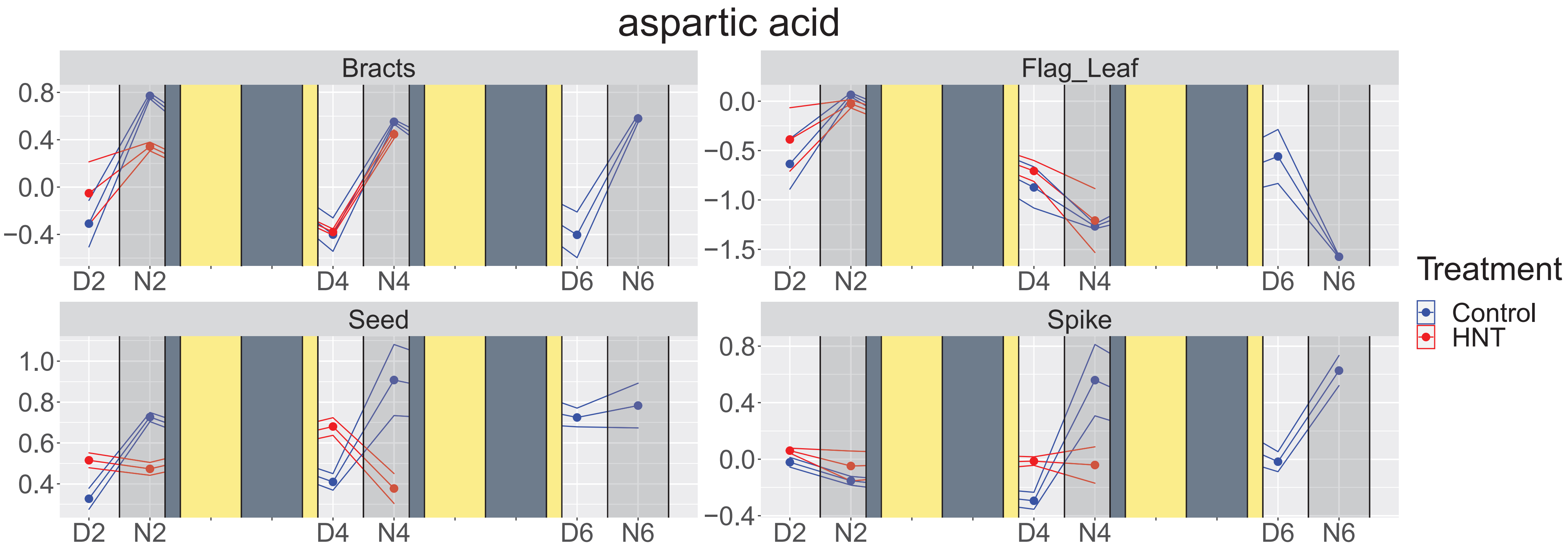
aspartic acid
Bracts
Flag_Leaf
Seed
Spike
D2
N2
D4
N4
D6
N6
Treatment
Control
HNT

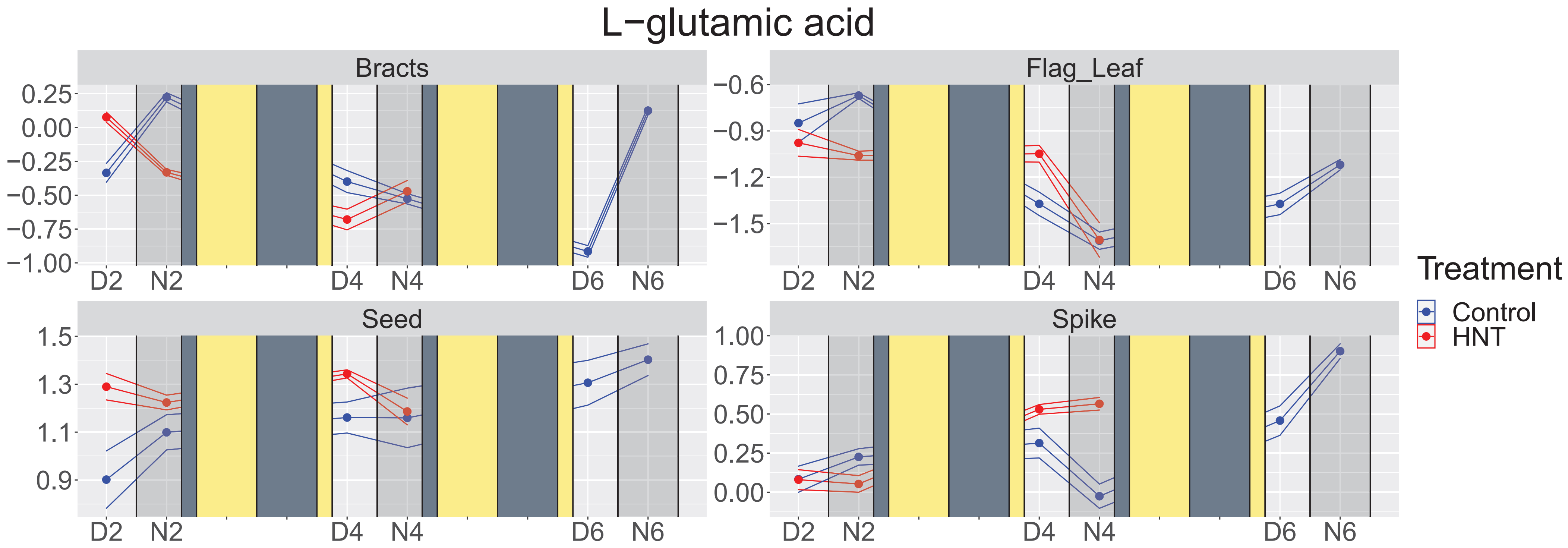
L−glutamic acid
Bracts
Flag_Leaf
Seed
Spike
0.25
0.00
−0.25
−0.50
−0.75
−1.00
−0.6
−0.9
−1.2
−1.5
1.5
1.3
1.1
0.9
1.00
0.75
0.50
0.25
0.00
D2
N2
D4
N4
D6
N6
Treatment
Control
HNT

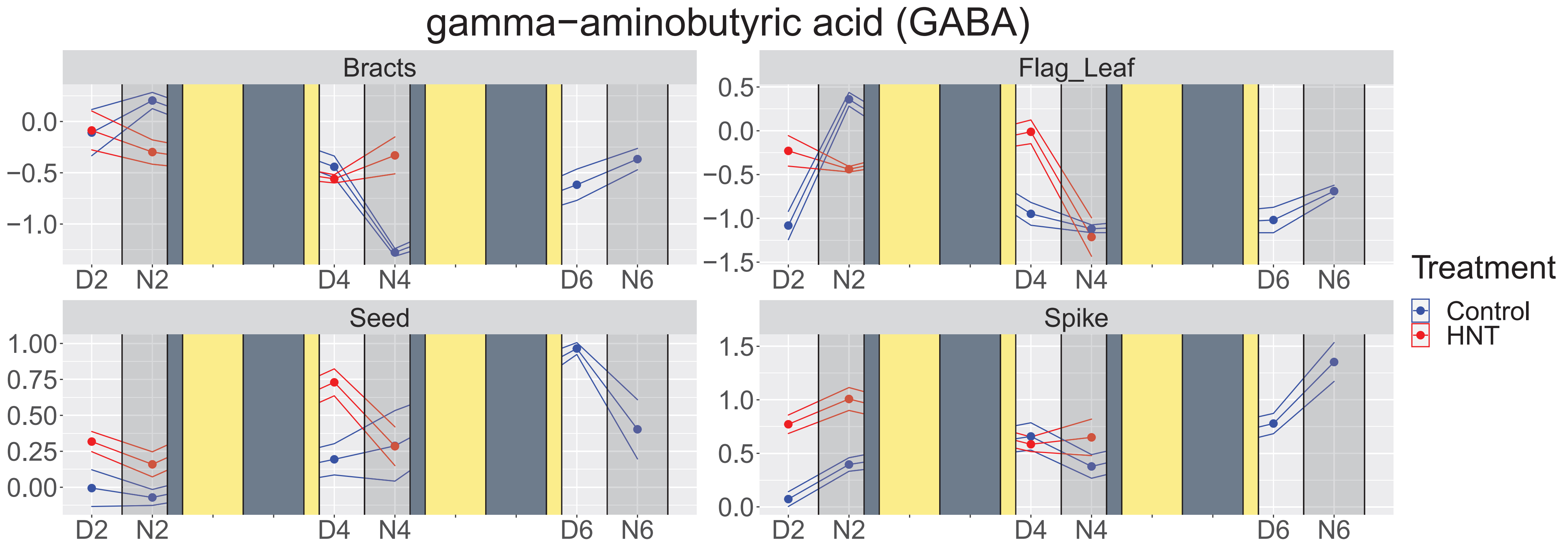

gamma−aminobutyric acid (GABA)
Bracts
Flag_Leaf
Seed
Spike
0.0
−0.5
−1.0
0.5
0.0
−0.5
−1.0
−1.5
1.00
0.75
0.50
0.25
0.00
1.5
1.0
0.5
0.0
D2
N2
D4
N4
D6
N6
Treatment
Control
HNT

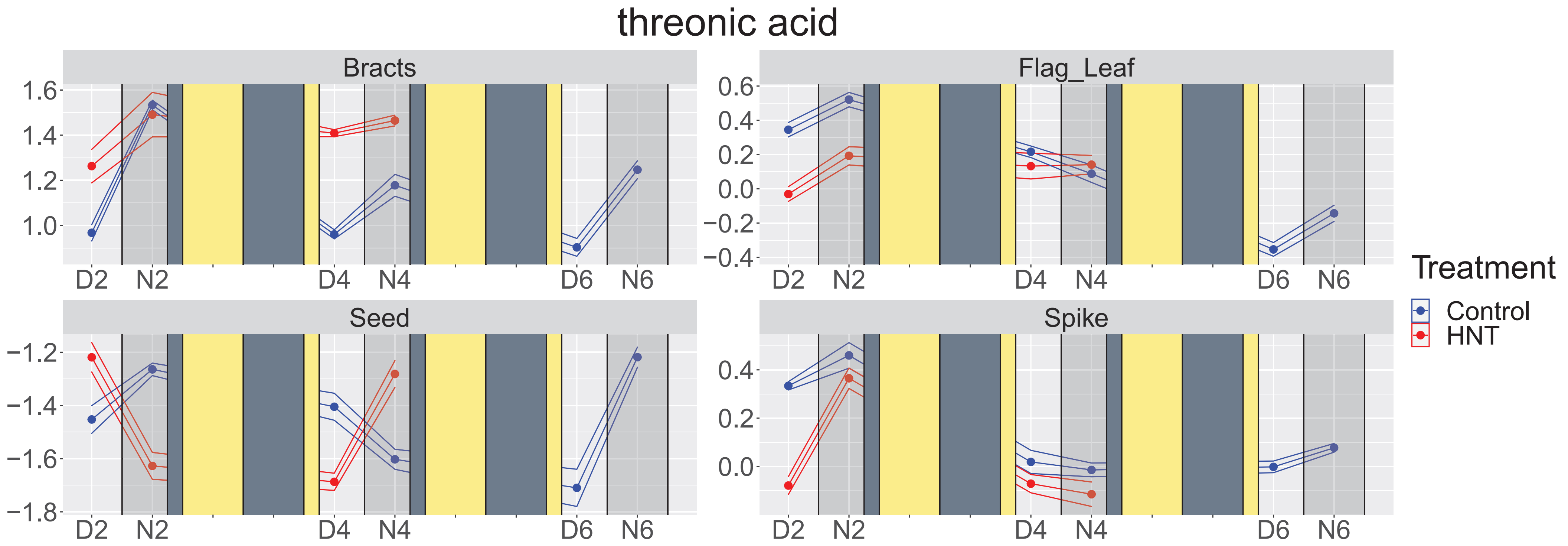
threonic acid
Bracts
Flag_Leaf
Seed
Spike
1.6
1.4
1.2
1.0
0.6
0.4
0.2
0.0
−0.2
−0.4
−1.2
−1.4
−1.6
−1.8
D2
N2
D4
N4
D6
N6
Treatment
Control
HNT

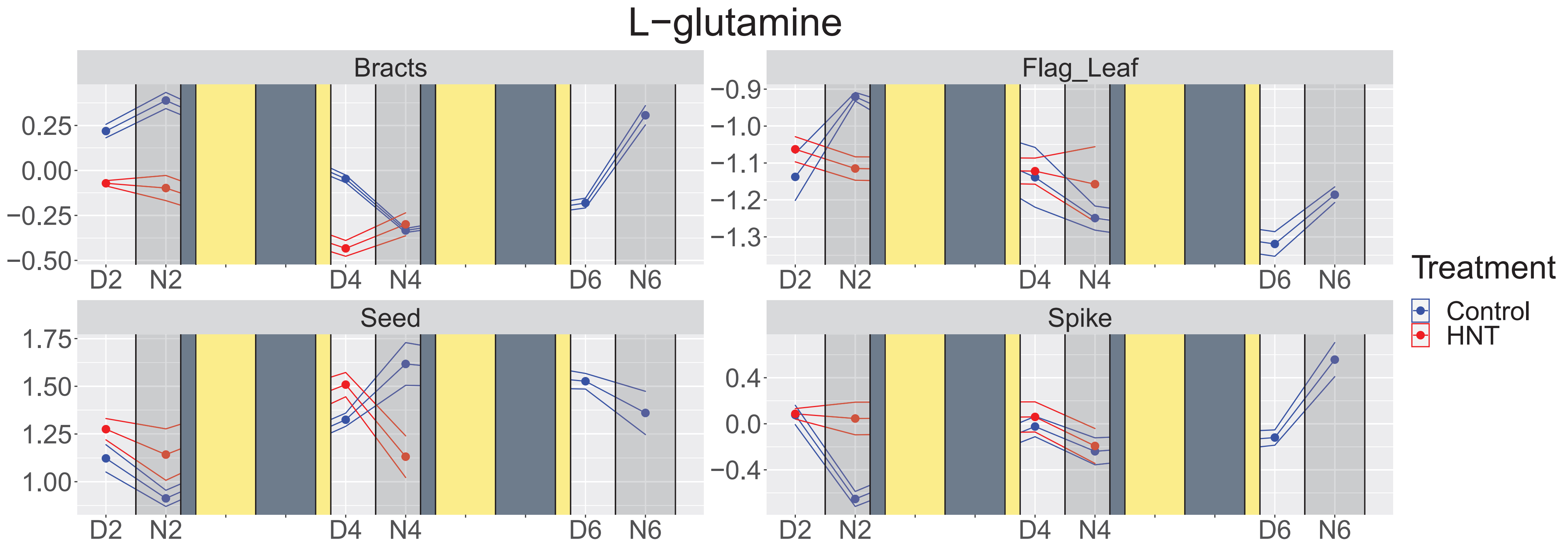

L−glutamine
Bracts
Flag_Leaf
Seed
Spike
0.25
0.00
−0.25
−0.50
−0.9
−1.0
−1.1
−1.2
−1.3
1.75
1.50
1.25
1.00
0.4
0.0
−0.4
D2
N2
D4
N4
D6
N6
Treatment
Control
HNT

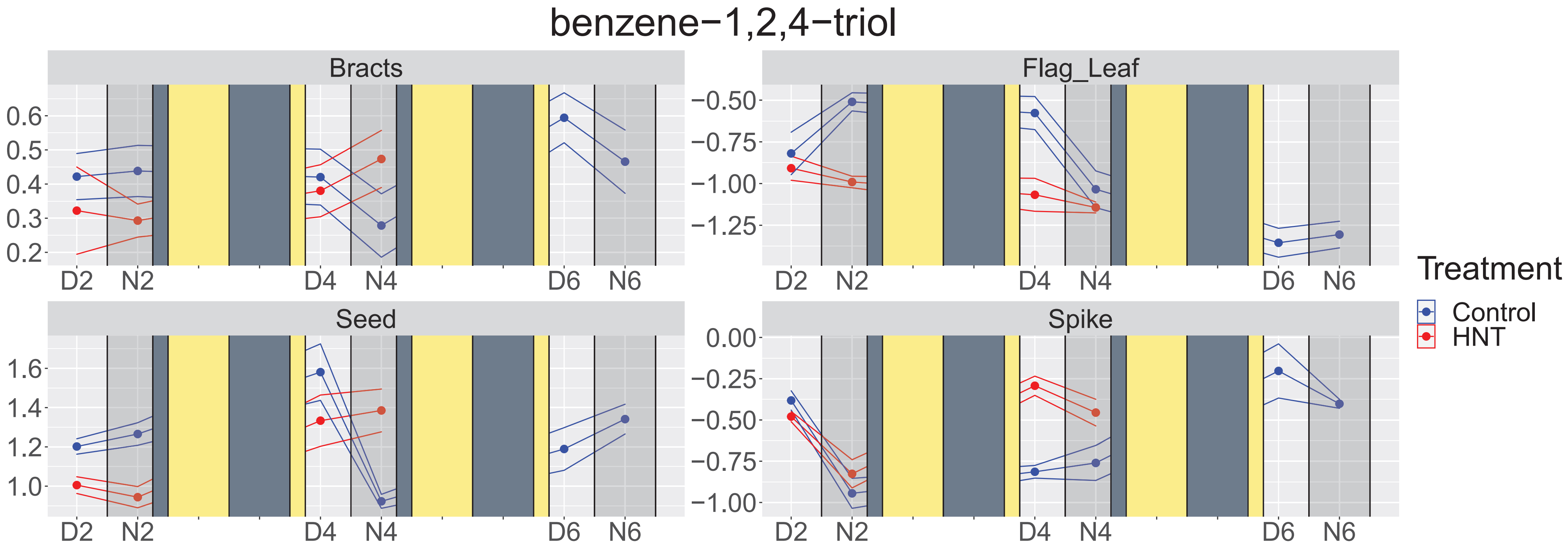

benzene−1,2,4−triol
Bracts
Flag_Leaf
Seed
Spike
0.6
0.5
0.4
0.3
0.2
−0.50
−0.75
−1.00
−1.25
1.6
1.4
1.2
1.0
0.00
−0.25
−0.50
−0.75
−1.00
D2
N2
D4
N4
D6
N6
Treatment
Control
HNT

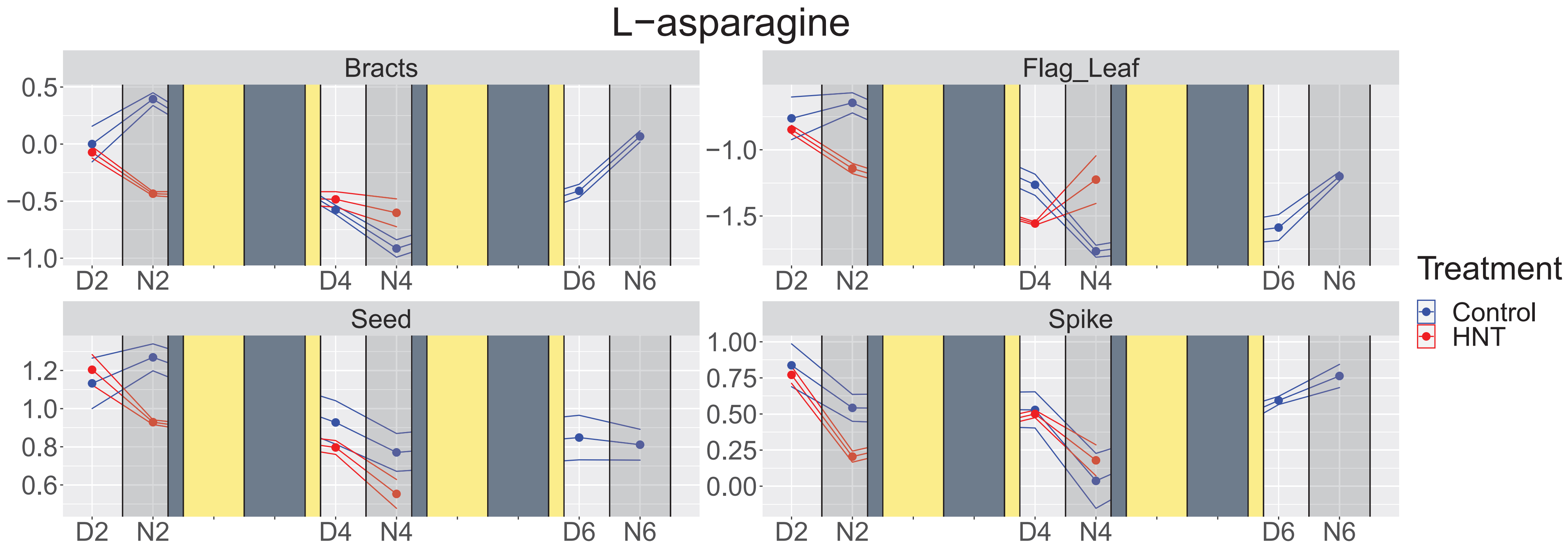
L−asparagine
Bracts
Flag_Leaf
Seed
Spike
0.5
0.0
−0.5
−1.0
−1.5
1.2
1.0
0.8
0.6
1.00
0.75
0.50
0.25
0.00
D2
N2
D4
N4
D6
N6
Treatment
Control
HNT

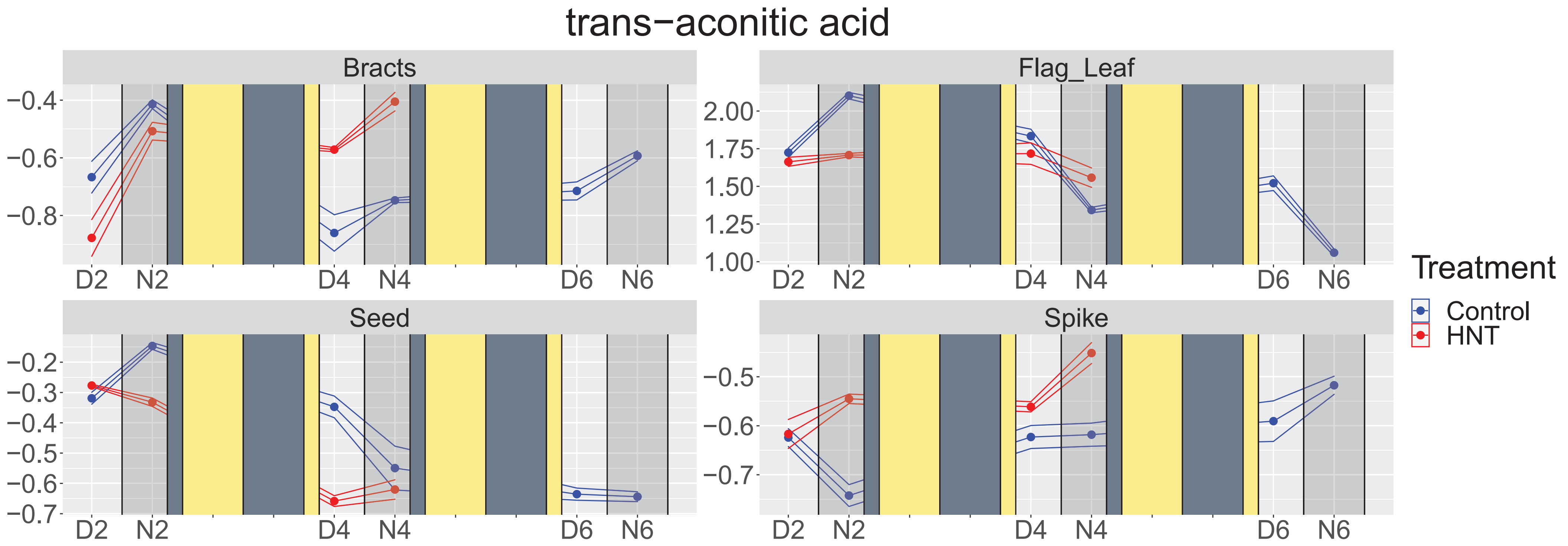

trans−aconitic acid
Bracts
Flag_Leaf
Seed
Spike
−0.4
−0.6
−0.8
2.00
1.75
1.50
1.25
1.00
−0.2
−0.3
−0.4
−0.5
−0.6
−0.7
−0.5
−0.6
−0.7
D2 N2 D4 N4 D6 N6
Treatment
Control
HNT

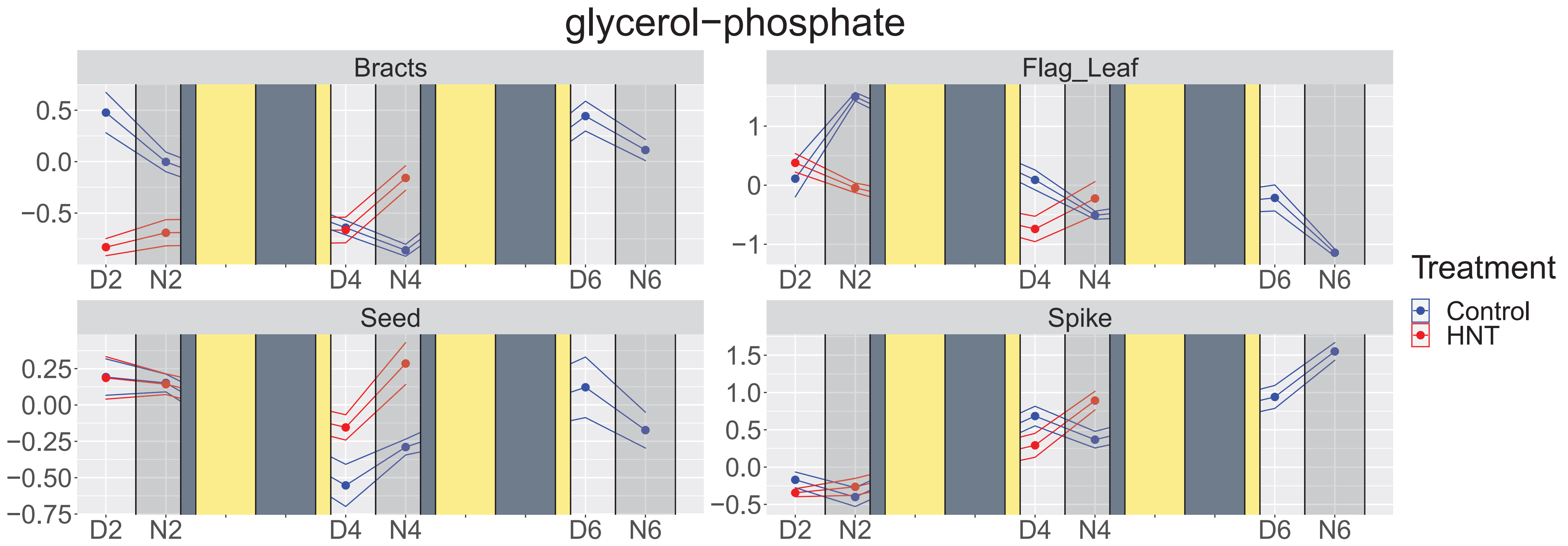
glycerol−phosphate
Bracts
Flag_Leaf
Seed
Spike
D2
N2
D4
N4
D6
N6
Treatment
Control
HNT
0.5
0.0
−0.5
1
0
−1
0.25
0.00
−0.25
−0.50
−0.75
1.5
1.0

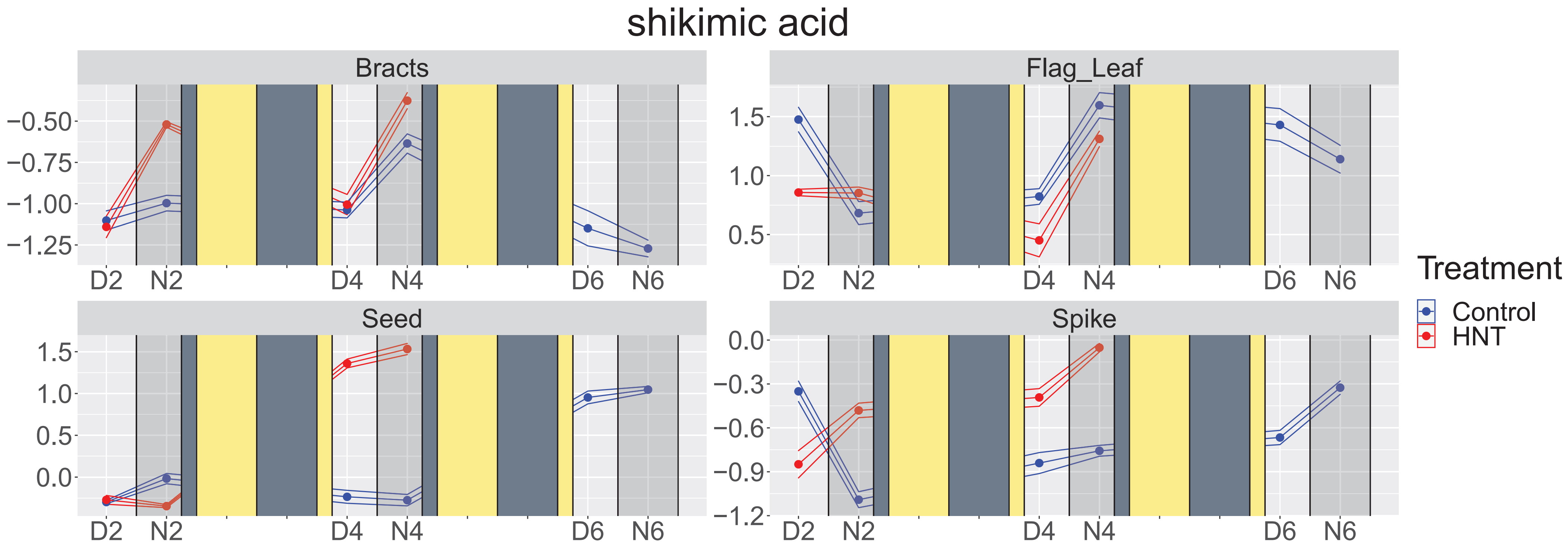

shikimic acid
Bracts
Flag_Leaf
Seed
Spike
−0.50
−0.75
−1.00
−1.25
1.5
1.0
0.5
0.0
0.0
−0.3
−0.6
−0.9
−1.2
D2
N2
D4
N4
D6
N6
Treatment
Control
HNT

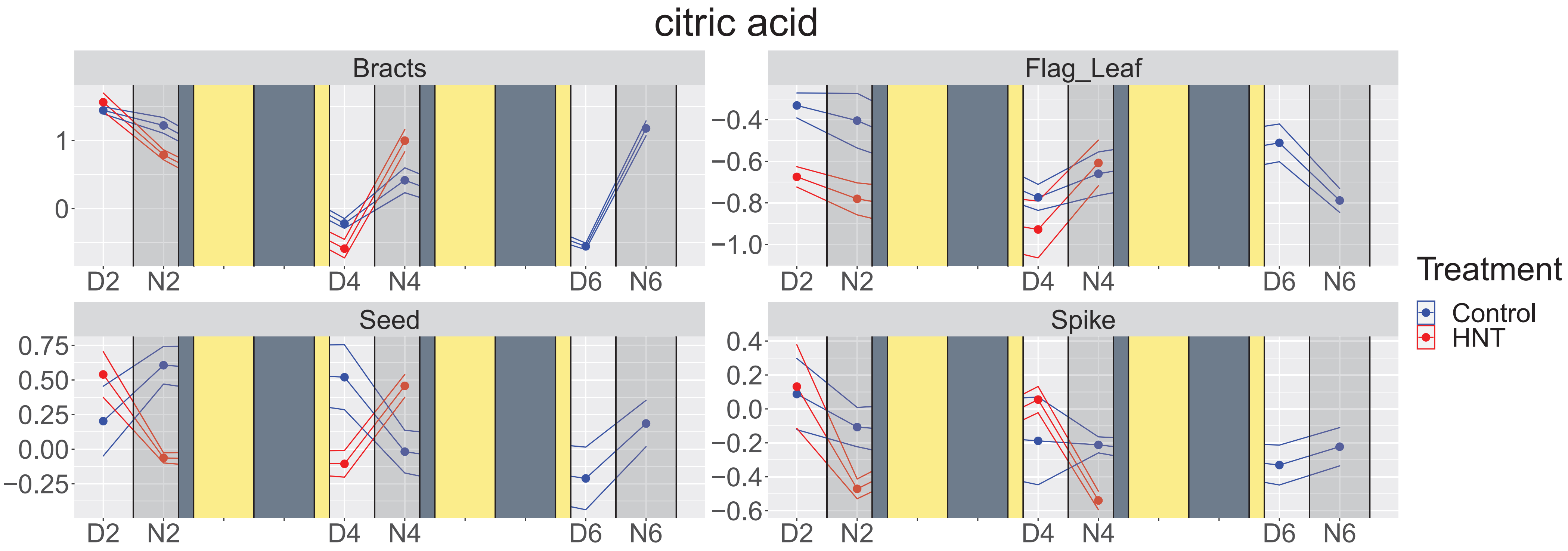
citric acid
Bracts
Flag_Leaf
Seed
Spike
1
0
−0.4
−0.6
−0.8
−1.0
0.75
0.50
0.25
0.00
−0.25
0.4
0.2
0.0
−0.2
−0.4
−0.6
D2
N2
D4
N4
D6
N6
Treatment
Control
HNT

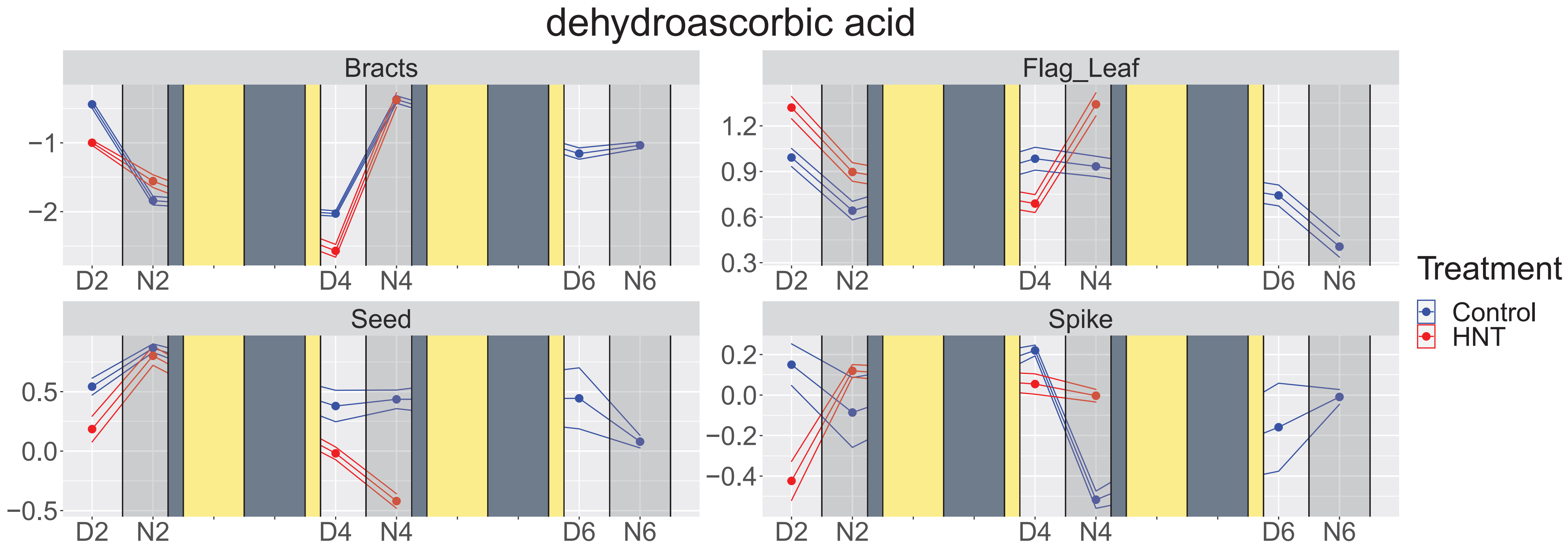
dehydroascorbic acid
Bracts
Flag_Leaf
Seed
Spike
−1
−2
1.2
0.9
0.6
0.3
0.5
0.0
−0.5
0.2
0.0
−0.2
−0.4
D2
N2
D4
N4
D6
N6
Treatment
Control
HNT

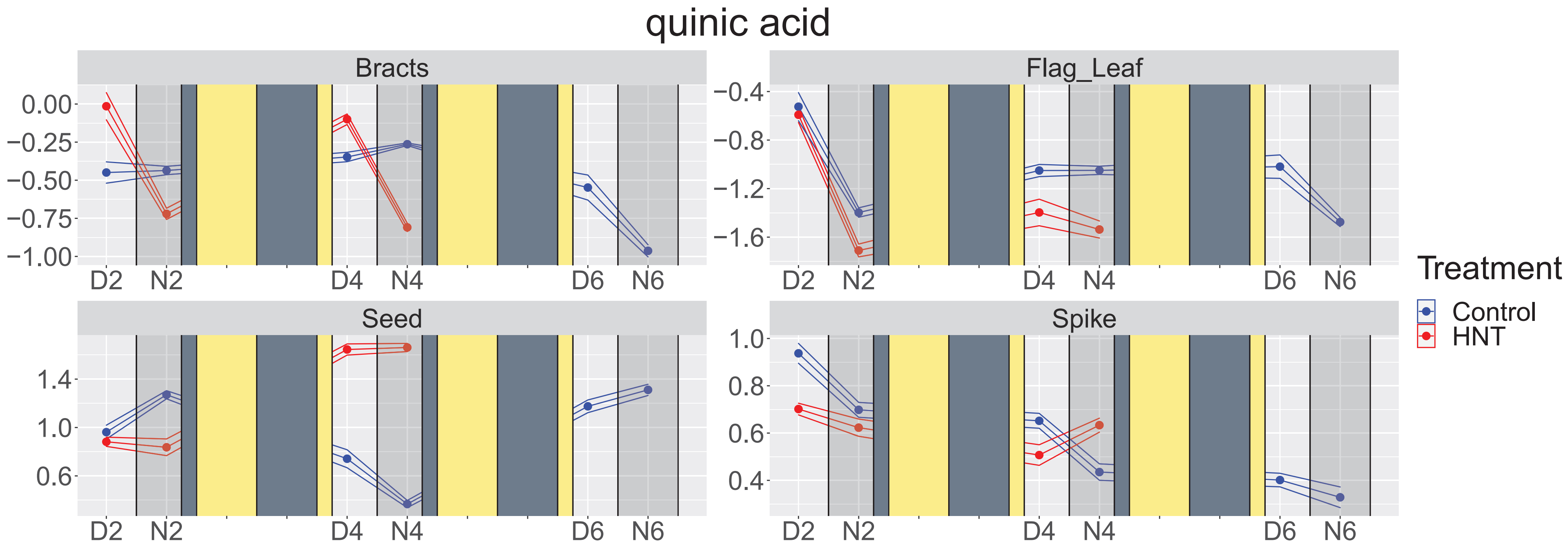
quinic acid
Bracts
Flag_Leaf
Seed
Spike
0.00
−0.25
−0.50
−0.75
−1.00
−0.4
−0.8
−1.2
−1.6
1.4
1.0
0.6
1.0
0.8
0.6
0.4
D2
N2
D4
N4
D6
N6
Treatment
Control
HNT

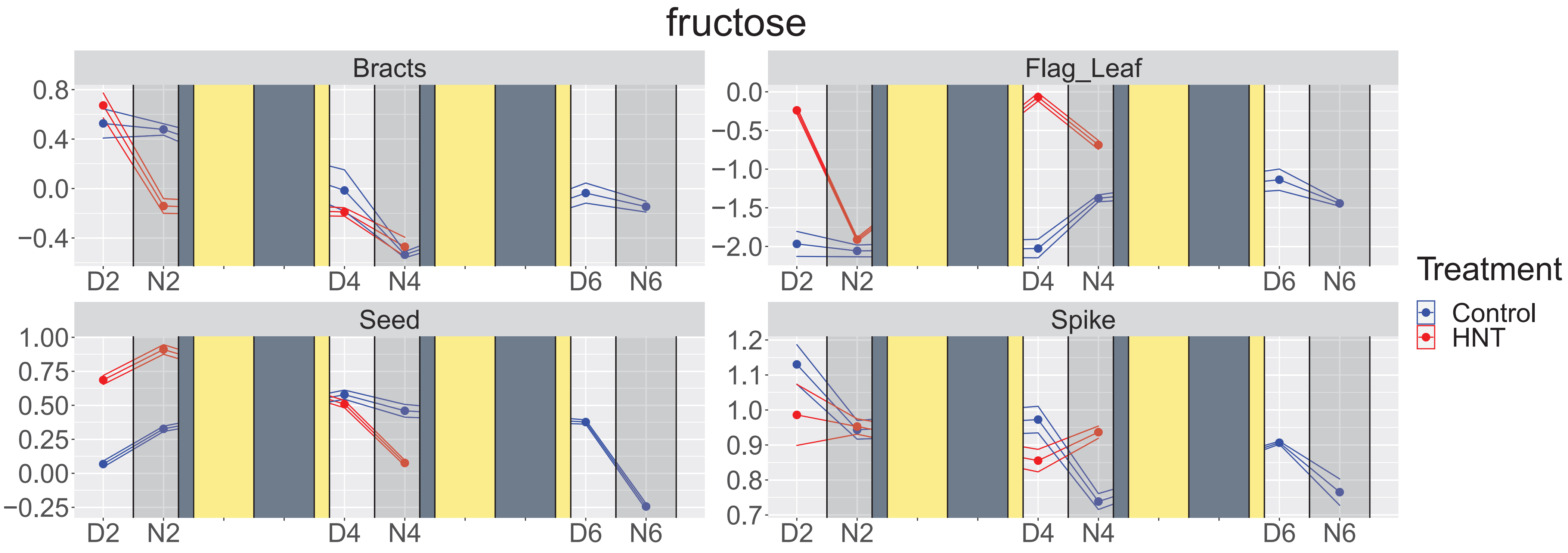

fructose
Bracts
Flag_Leaf
Seed
Spike
0.8
0.4
0.0
−0.4
0.0
−0.5
−1.0
−1.5
−2.0
1.00
0.75
0.50
0.25
0.00
−0.25
1.2
1.1
1.0
0.9
0.8
0.7
D2
N2
D4
N4
D6
N6
Treatment
Control
HNT

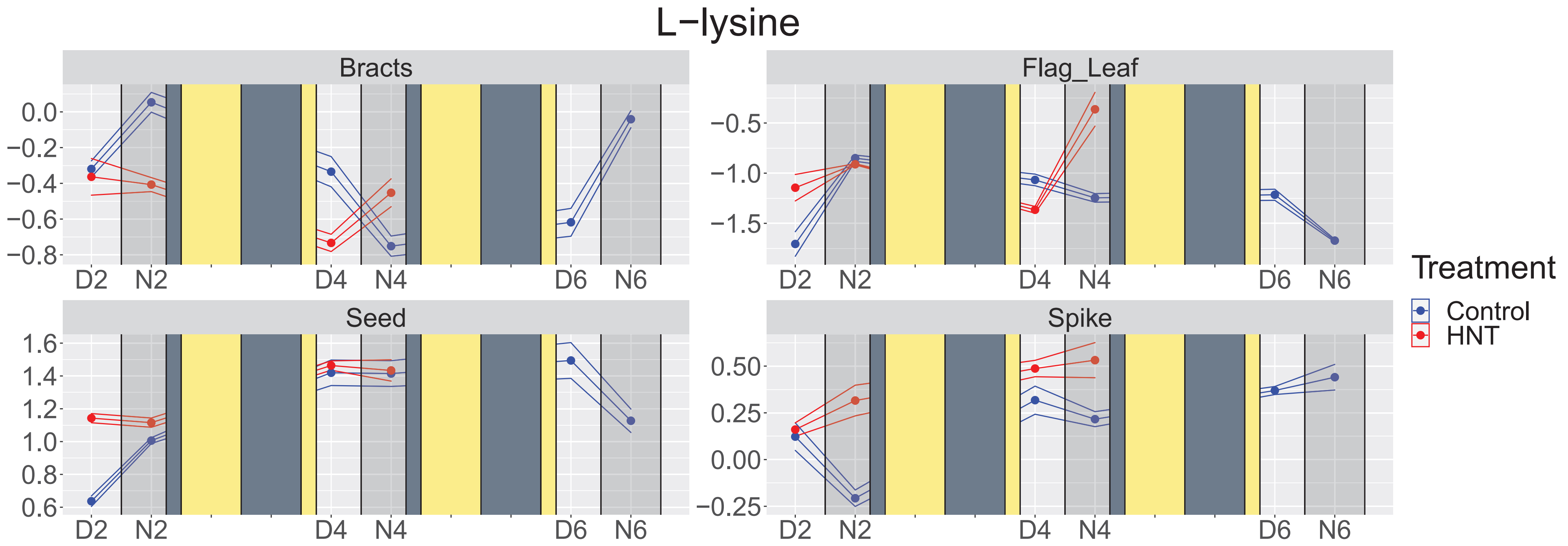

L−lysine
Bracts
Flag_Leaf
Seed
Spike
0.0
−0.2
−0.4
−0.6
−0.8
−0.5
−1.0
−1.5
1.6
1.4
1.2
1.0
0.8
0.6
0.50
0.25
0.00
−0.25
D2
N2
D4
N4
D6
N6
Treatment
Control
HNT

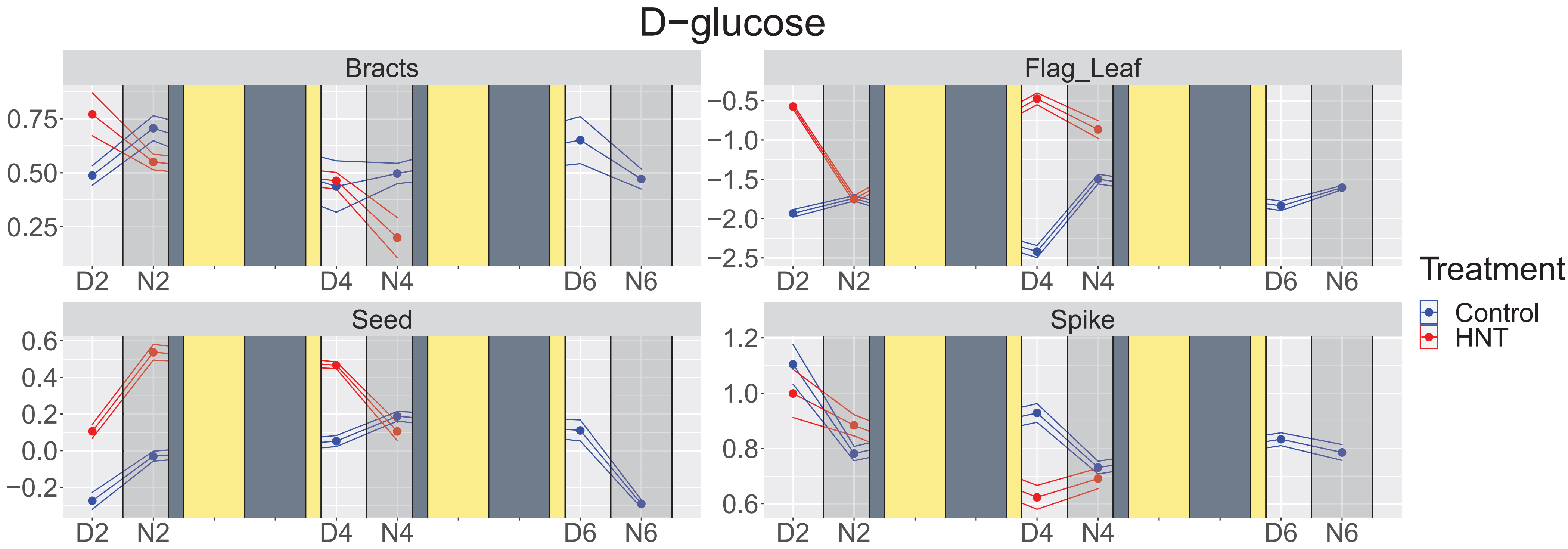

D−glucose
Bracts
Flag_Leaf
Seed
Spike
0.75
0.50
0.25
−0.5
−1.0
−1.5
−2.0
−2.5
0.6
0.4
0.2
0.0
−0.2
1.2
1.0
0.8
0.6
D2
N2
D4
N4
D6
N6
Treatment
Control
HNT

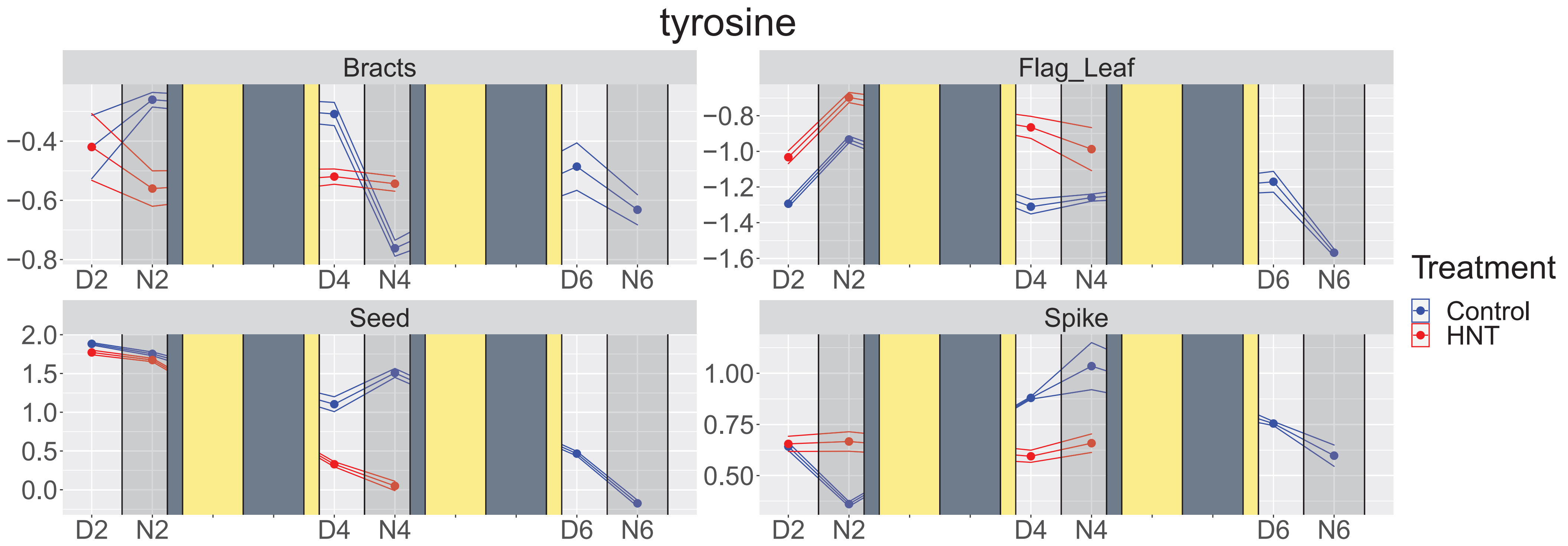

tyrosine
Bracts
Flag_Leaf
Seed
Spike
Treatment
Control
HNT
D2
N2
D4
N4
D6
N6

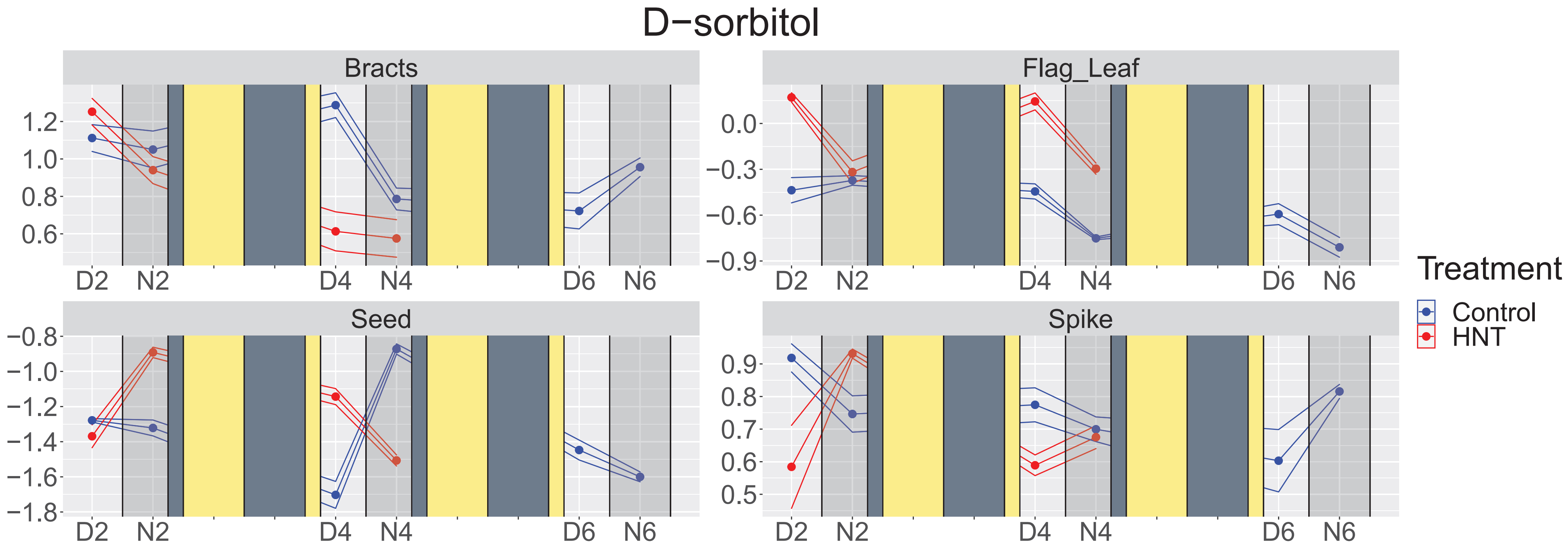

D−sorbitol
Bracts
Flag_Leaf
Seed
Spike
1.2
1.0
0.8
0.6
0.0
−0.3
−0.6
−0.9
−0.8
−1.0
−1.2
−1.4
−1.6
−1.8
0.9
0.8
0.7
0.6
0.5
D2
N2
D4
N4
D6
N6
Treatment
Control
HNT

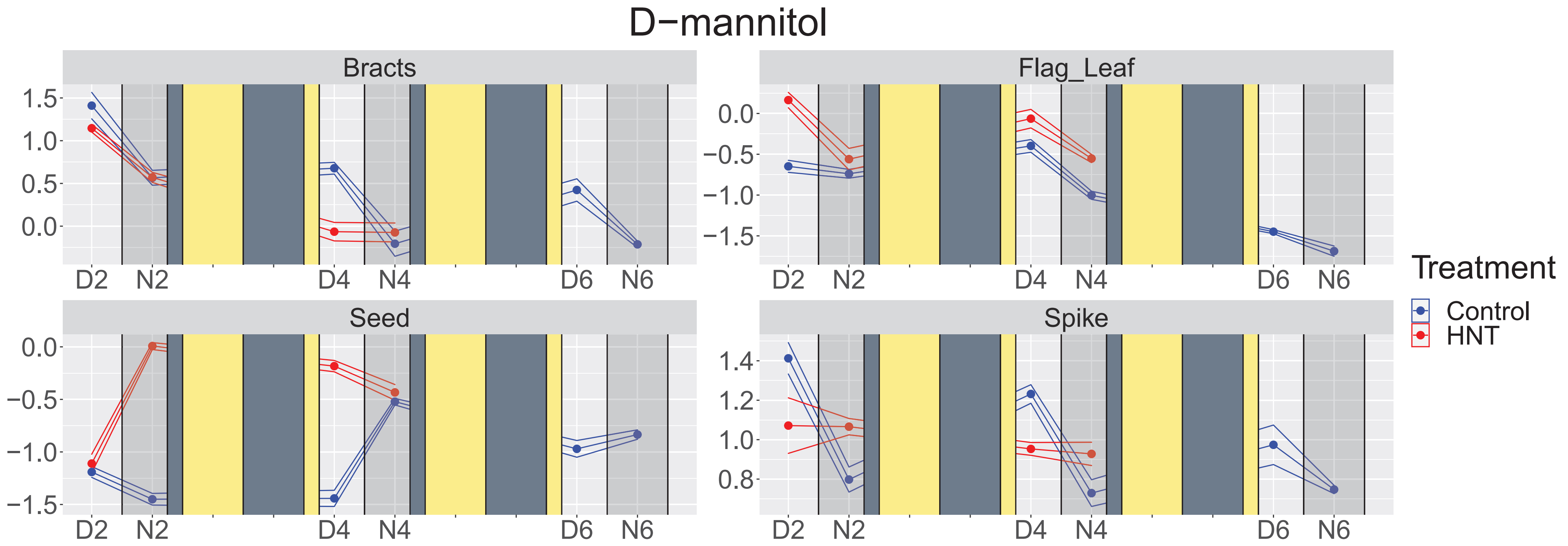

D−mannitol
Bracts
Flag_Leaf
Seed
Spike
1.5
1.0
0.5
0.0
−0.5
−1.0
−1.5
1.4
1.2
0.8
D2
N2
D4
N4
D6
N6
Treatment
Control
HNT

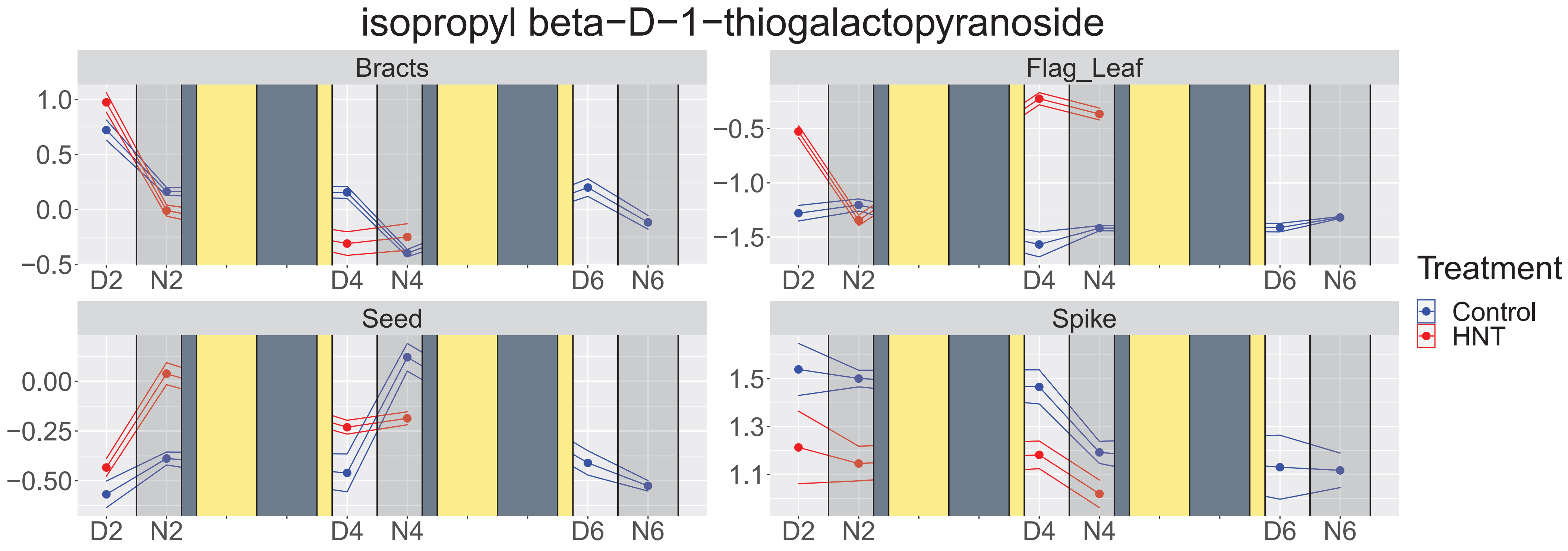

isopropyl beta−D−1−thiogalactopyranoside
Bracts
Flag_Leaf
Seed
Spike
1.0
0.5
0.0
−0.5
−1.0
−1.5
0.00
−0.25
−0.50
1.5
1.3
1.1
D2
N2
D4
N4
D6
N6
Treatment
Control
HNT

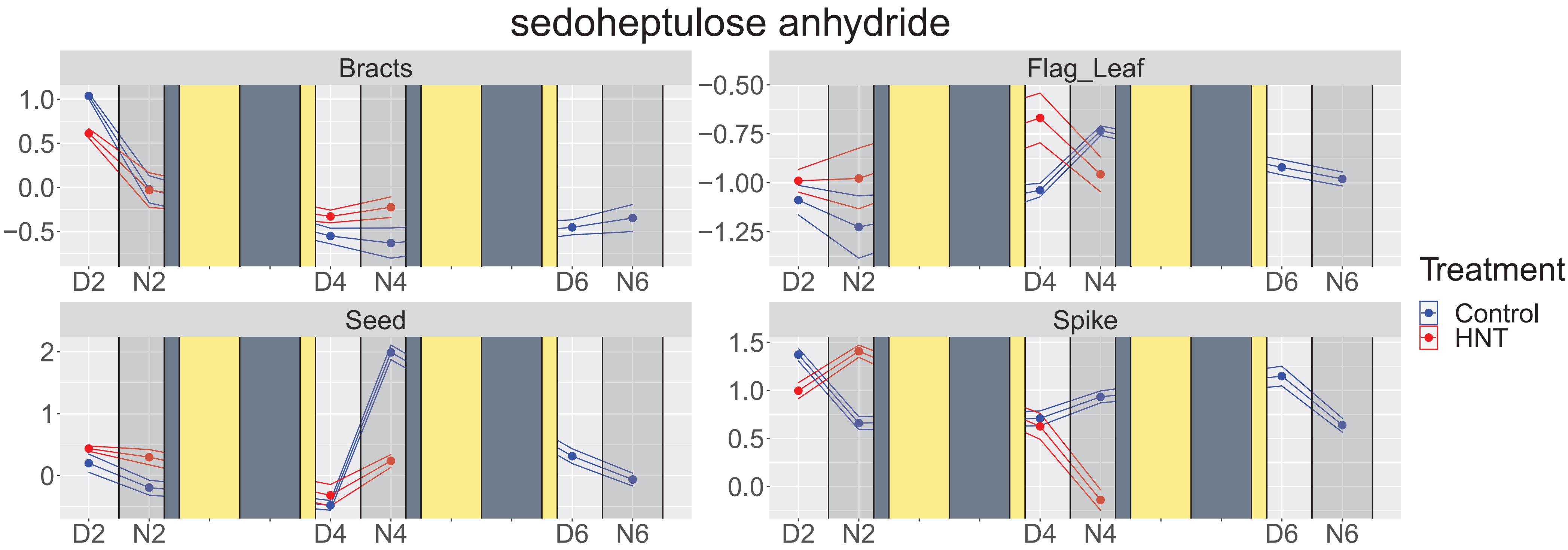

sedoheptulose anhydride
Bracts
Flag_Leaf
Seed
Spike
1.0
0.5
0.0
−0.5
−0.50
−0.75
−1.00
−1.25
2
1
0
1.5
1.0
0.5
0.0
D2
N2
D4
N4
D6
N6
Treatment
Control
HNT

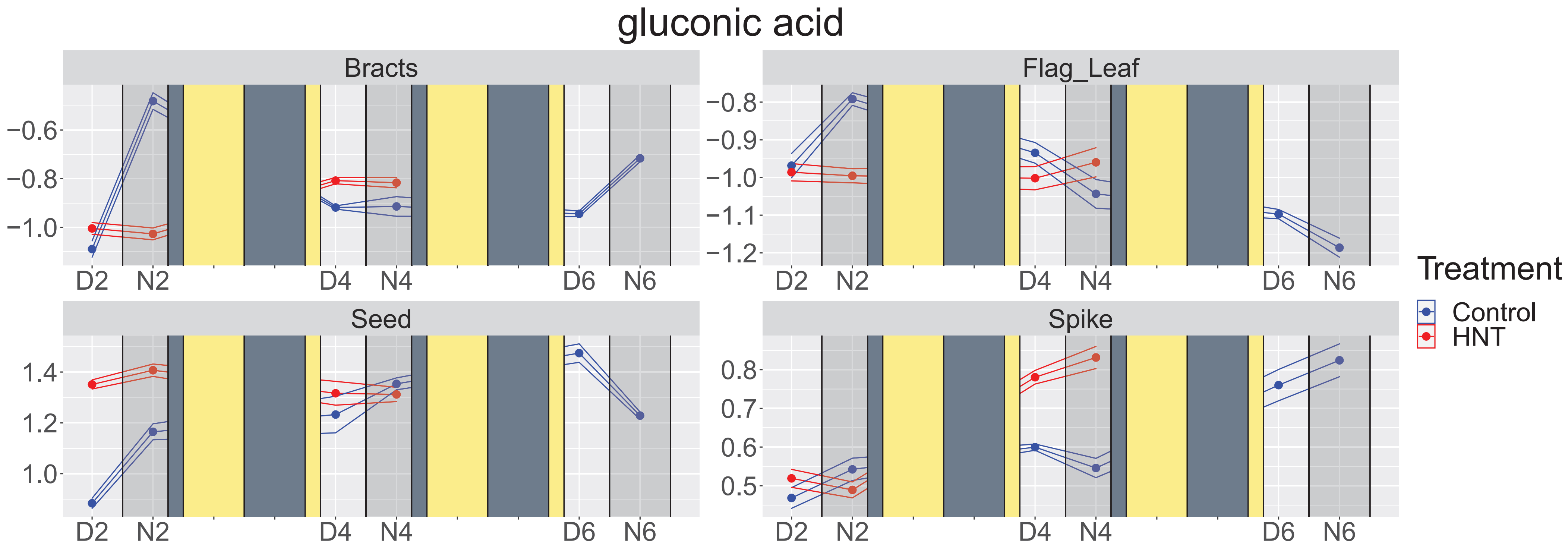

gluconic acid
Bracts
Flag_Leaf
Seed
Spike
−0.6
−0.8
−1.0
−0.8
−0.9
−1.0
−1.1
−1.2
1.4
1.2
1.0
0.8
0.7
0.6
0.5
D2
N2
D4
N4
D6
N6
Treatment
Control
HNT

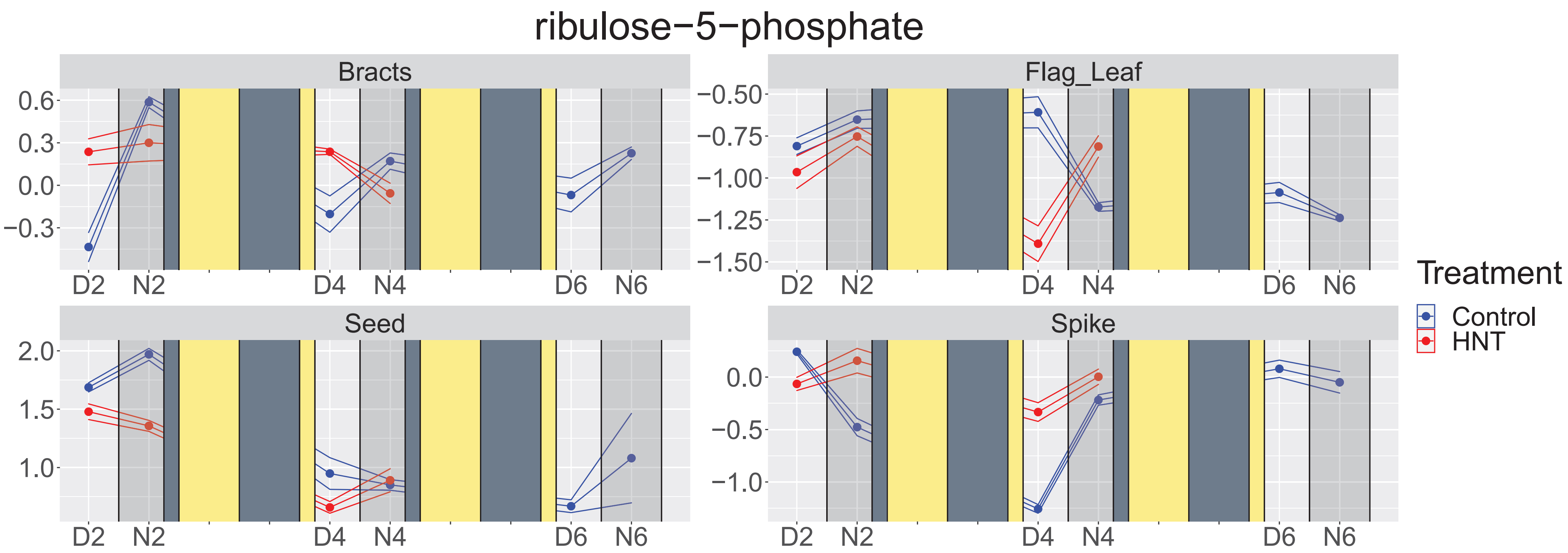

ribulose−5−phosphate
Bracts
Flag_Leaf
Seed
Spike
0.6
0.3
0.0
−0.3
−0.50
−0.75
−1.00
−1.25
−1.50
2.0
1.5
1.0
0.0
−0.5
−1.0
D2 N2 D4 N4 D6 N6
Treatment
Control
HNT

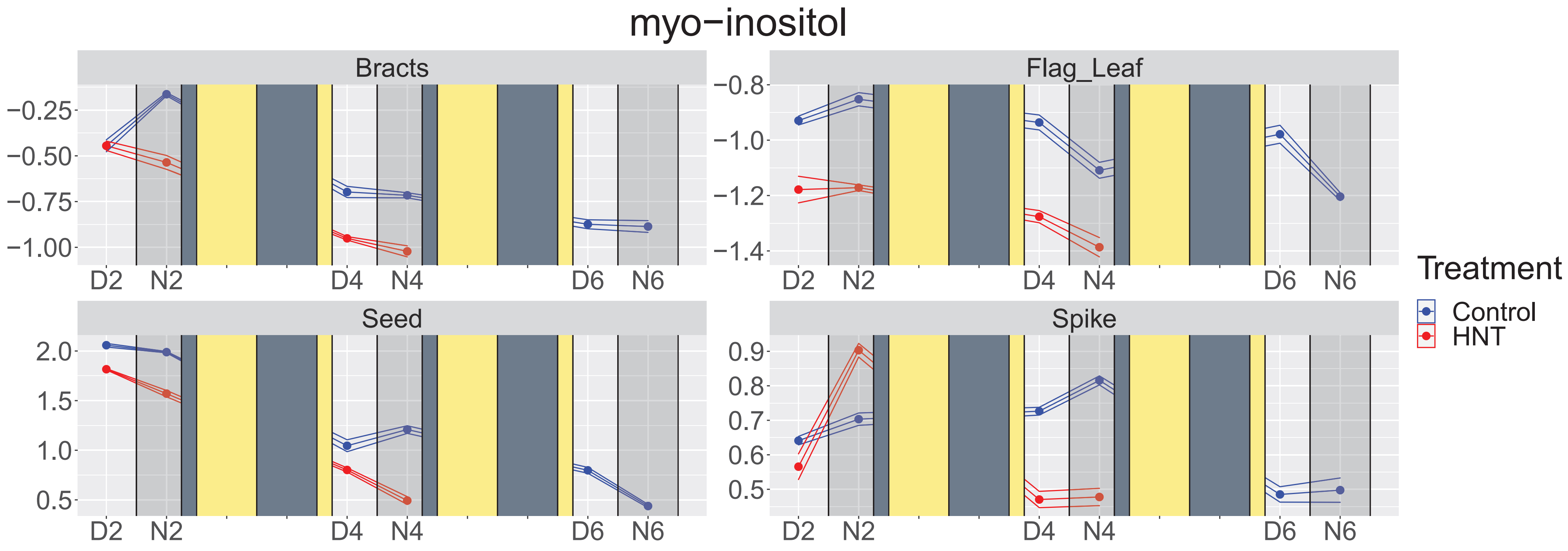

myo−inositol
Bracts
Flag_Leaf
Seed
Spike
−0.25
−0.50
−0.75
−1.00
−0.8
−1.0
−1.2
−1.4
2.0
1.5
1.0
0.5
0.9
0.8
0.7
0.6
0.5
D2
N2
D4
N4
D6
N6
Treatment
Control
HNT

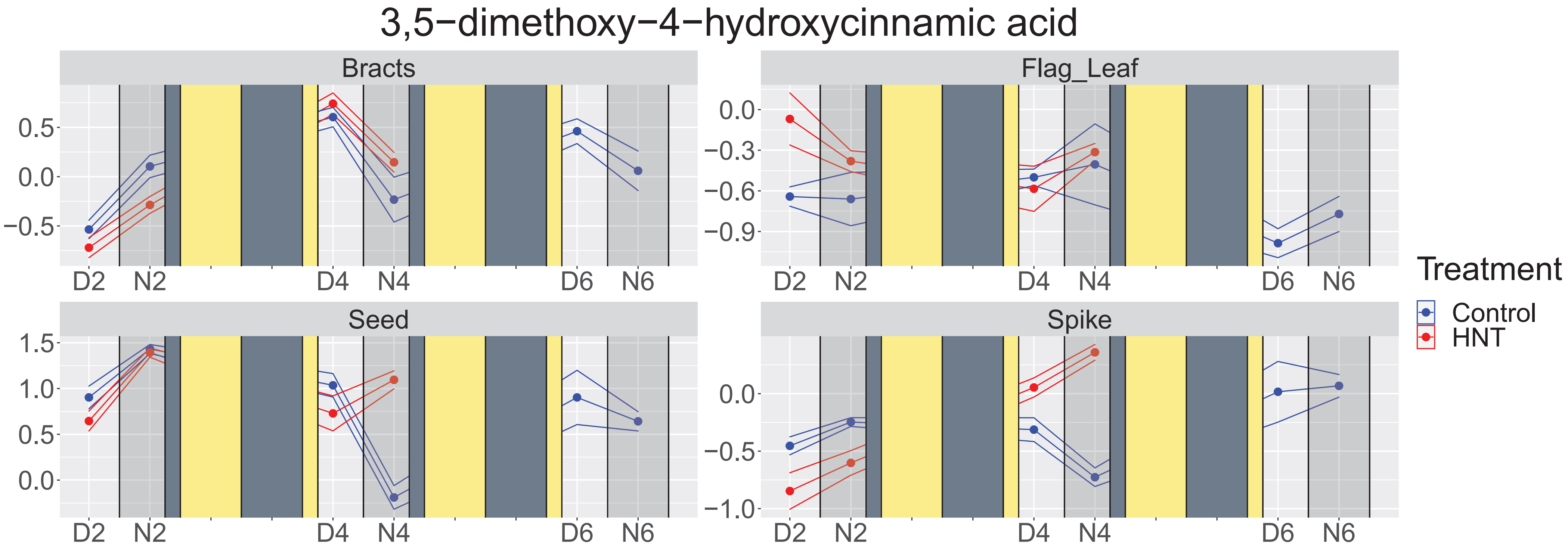
3,5−dimethoxy−4−hydroxycinnamic acid
Bracts
Flag_Leaf
Seed
Spike
D2
N2
D4
N4
D6
N6
Treatment
Control
HNT

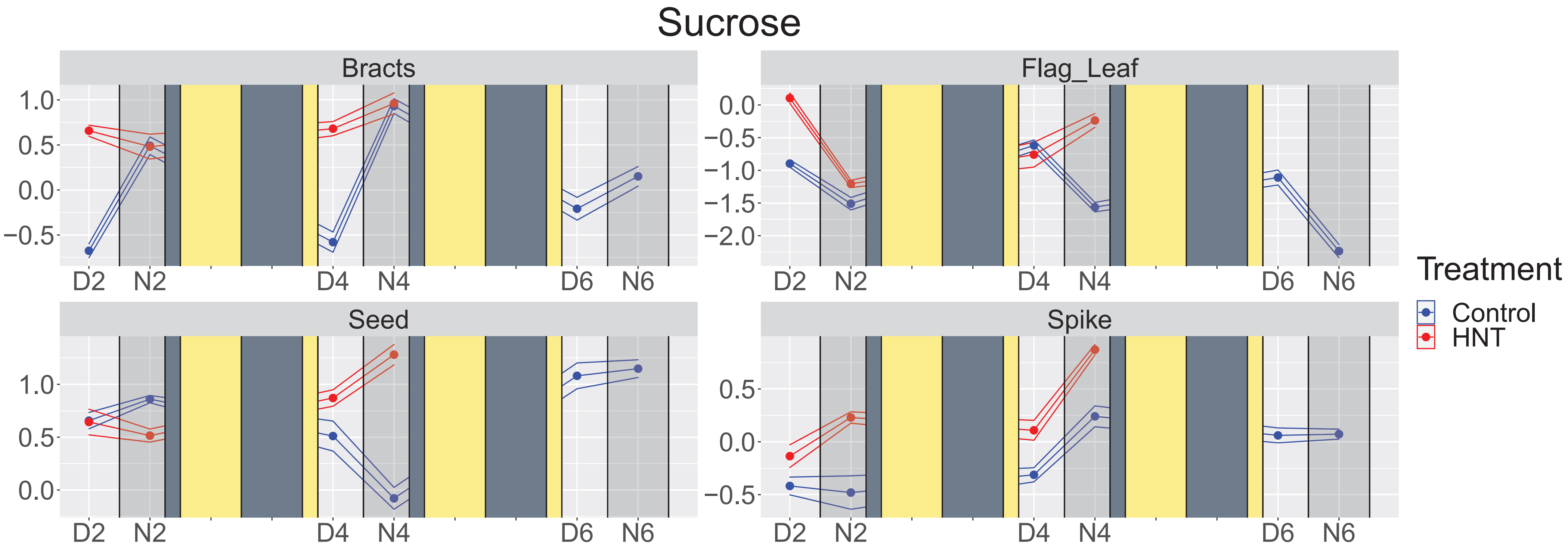

Sucrose
Bracts
Flag_Leaf
Seed
Spike
1.0
0.5
0.0
−0.5
−1.0
−1.5
−2.0
D2
N2
D4
N4
D6
N6
Treatment
Control
HNT

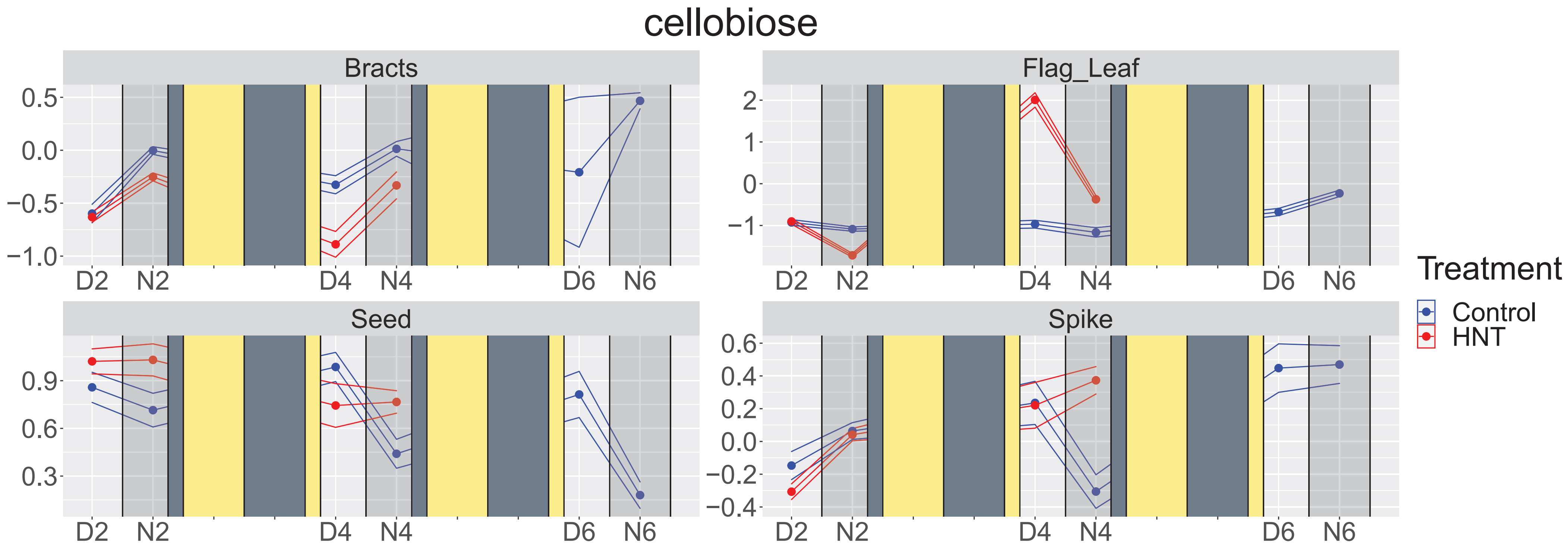
cellobiose
Bracts
Flag_Leaf
Seed
Spike
Treatment
Control
HNT
D2
N2
D4
N4
D6
N6

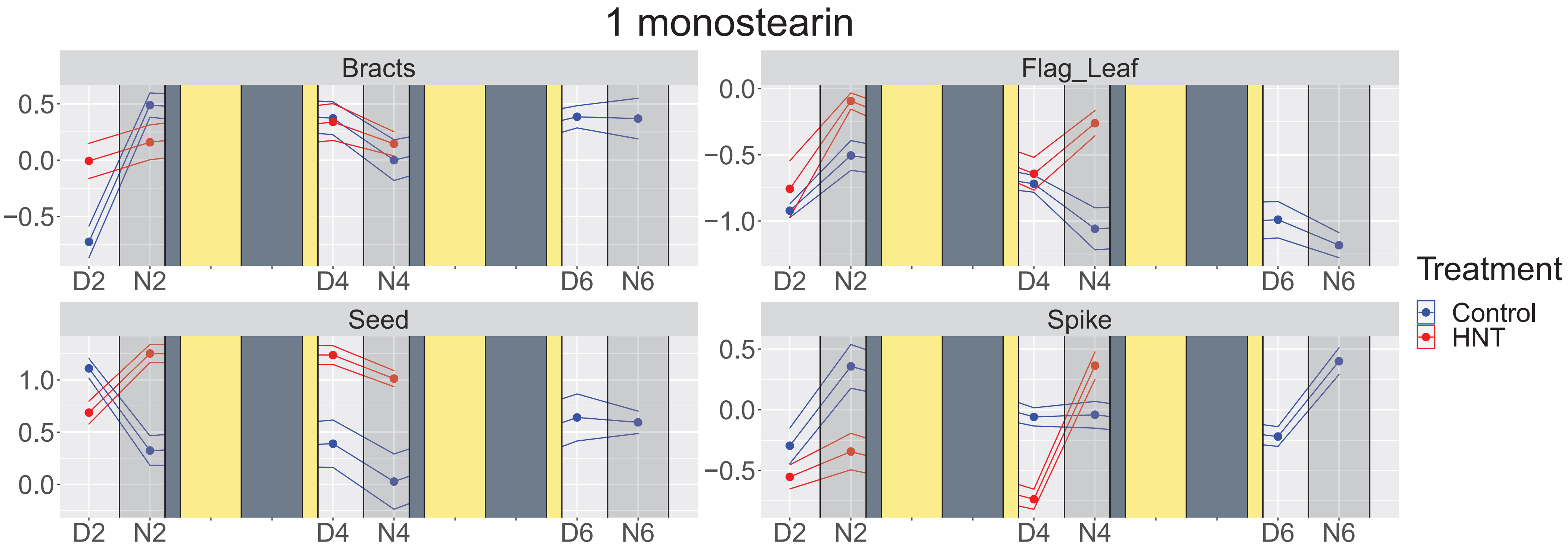

1 monostearin
Bracts
Flag_Leaf
Seed
Spike
0.5
0.0
−0.5
−1.0
1.0
D2
N2
D4
N4
D6
N6
Treatment
Control
HNT

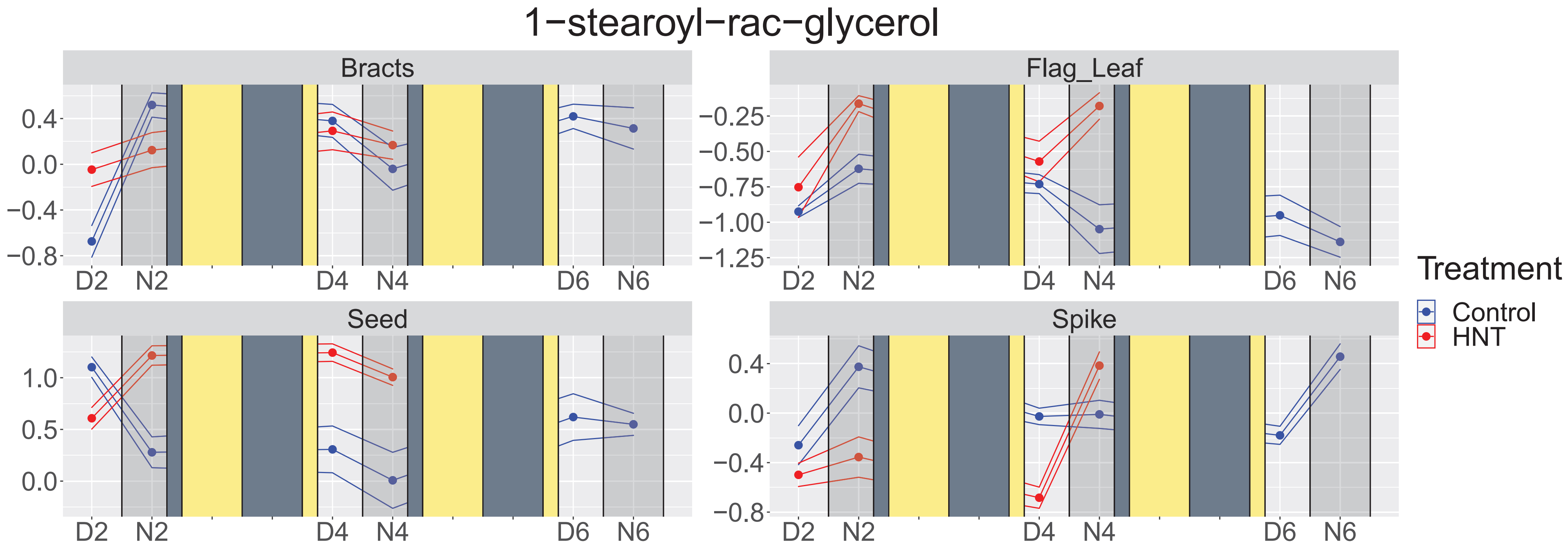

1−stearoyl−rac−glycerol
Bracts
Flag_Leaf
Seed
Spike
0.4
0.0
−0.4
−0.8
−0.25
−0.50
−0.75
−1.00
−1.25
1.0
0.5
0.0
0.4
0.0
−0.4
−0.8
D2
N2
D4
N4
D6
N6
Treatment
Control
HNT

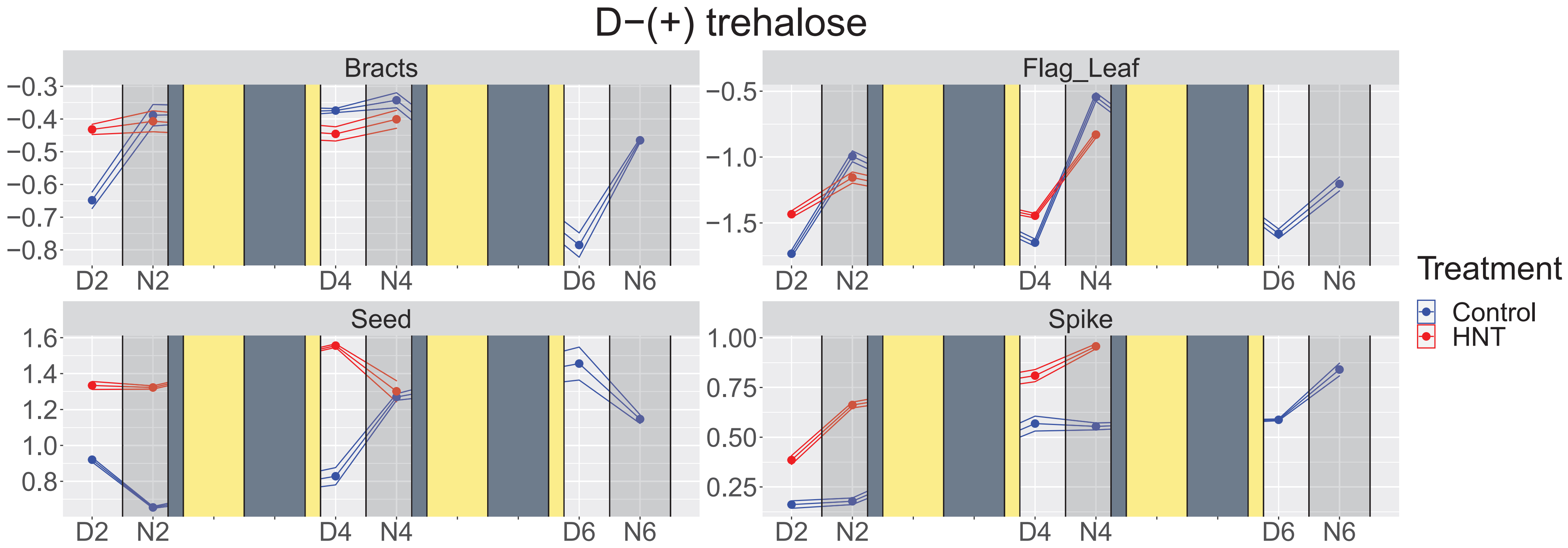

D−(+) trehalose
Bracts
Flag_Leaf
Seed
Spike
−0.3
−0.4
−0.5
−0.6
−0.7
−0.8
−0.5
−1.0
−1.5
1.6
1.4
1.2
1.0
0.8
1.00
0.75
0.50
0.25
D2
N2
D4
N4
D6
N6
Treatment
Control
HNT

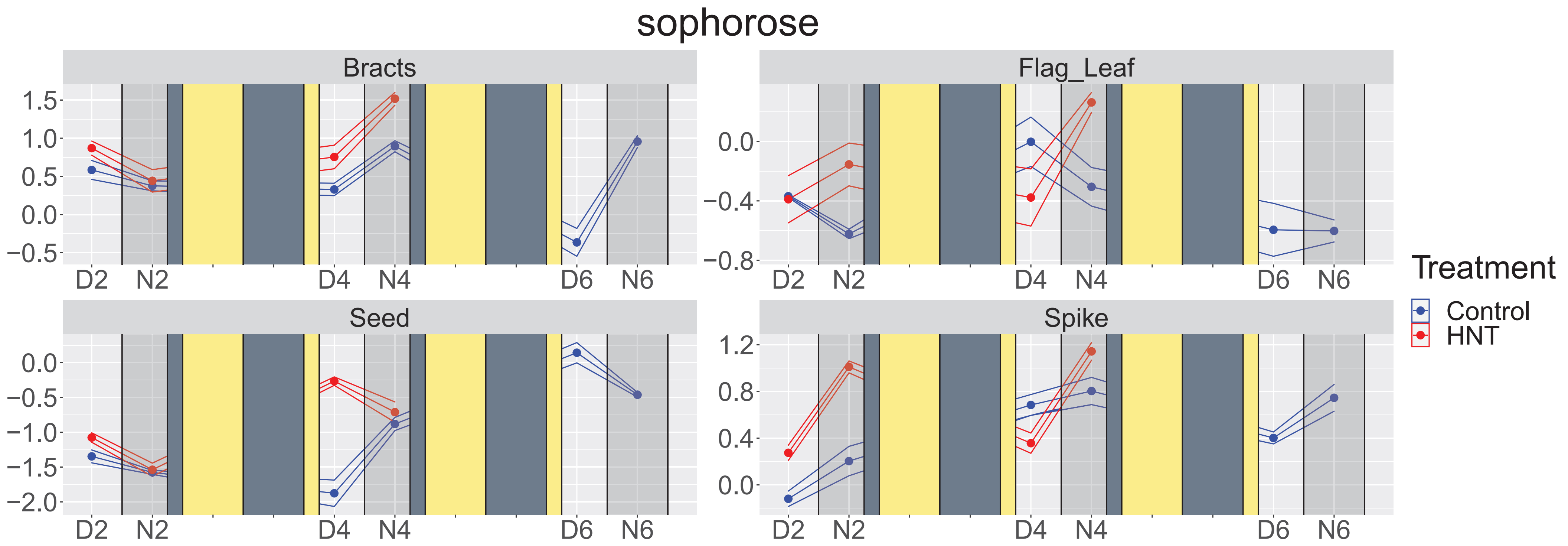

sophorose
Bracts
Flag_Leaf
Seed
Spike
D2
N2
D4
N4
D6
N6
Treatment
Control
HNT

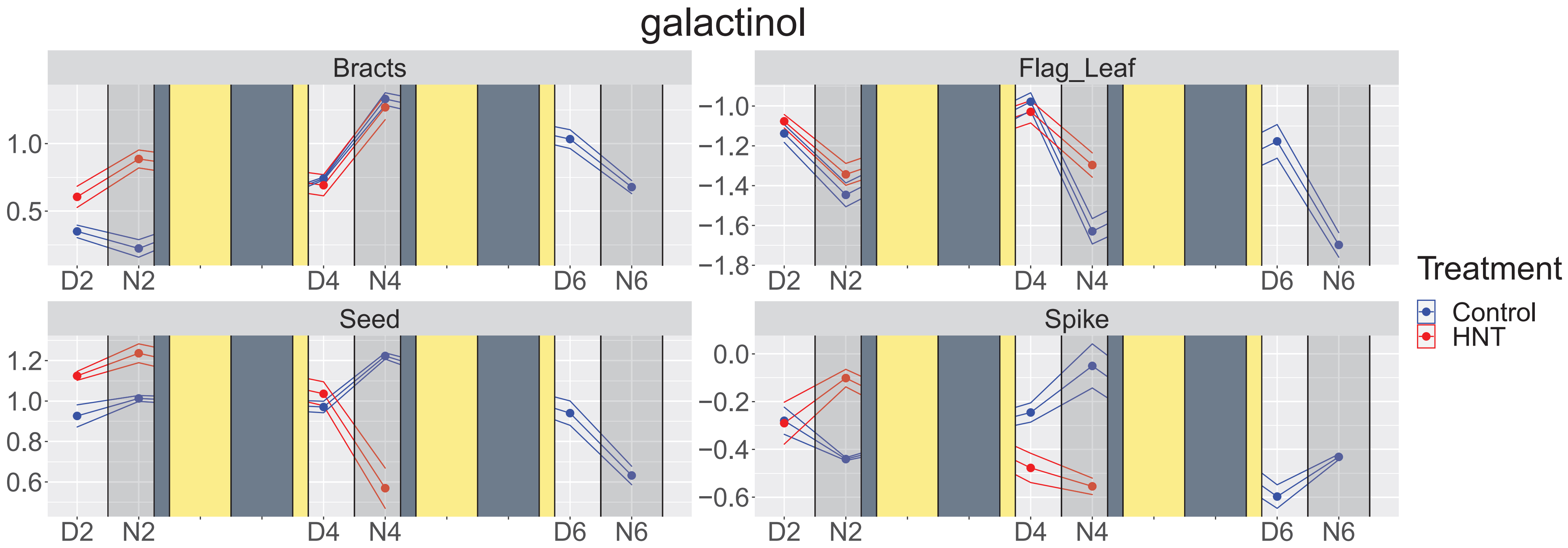

galactinol
Bracts
Flag_Leaf
Seed
Spike
1.0
0.5
−1.0
−1.2
−1.4
−1.6
−1.8
1.2
1.0
0.8
0.6
0.0
−0.2
−0.4
−0.6
D2
N2
D4
N4
D6
N6
Treatment
Control
HNT

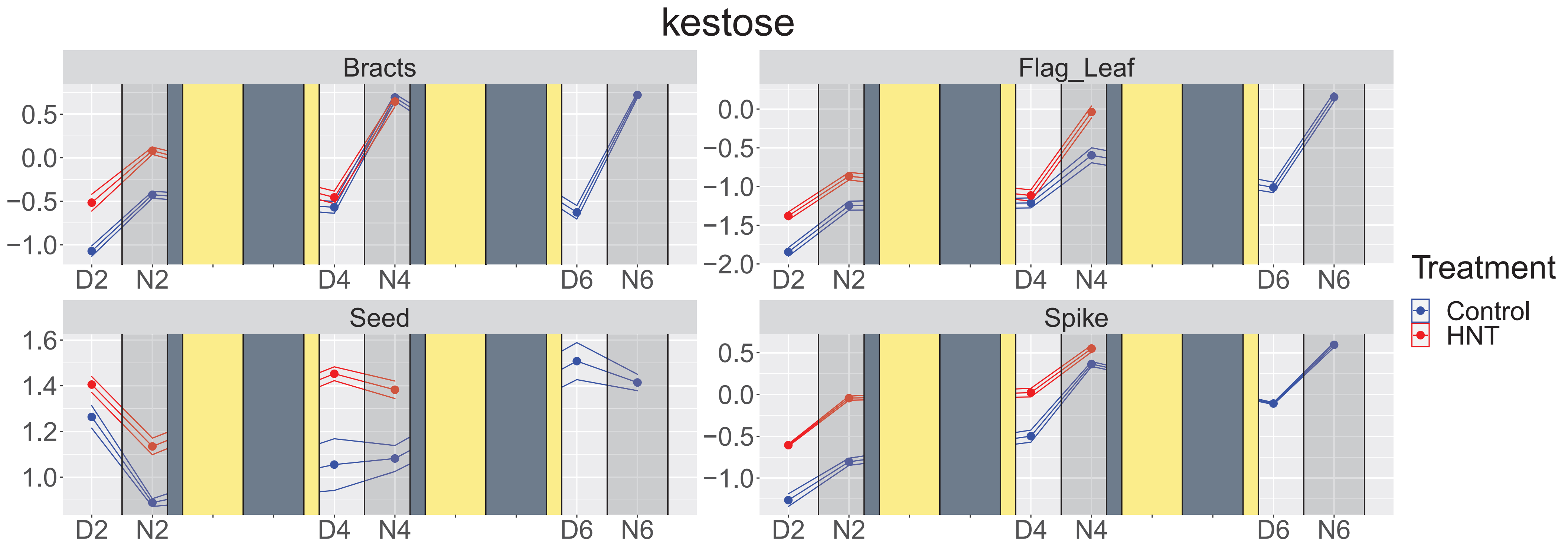
kestose
Bracts
Flag_Leaf
Seed
Spike
0.5
0.0
−0.5
−1.0
−1.5
−2.0
1.6
1.4
1.2
1.0
D2
N2
D4
N4
D6
N6
Treatment
Control
HNT
