# Supplementary material for: Tissue- and time-dependent metabolite profiles during early grain development under normal and high night-time temperature conditions
Source: BMC Plant Biol. 2024 Jun 18;24:568. doi: 10.1186/s12870-024-05190-6 (PMC11184705; doi:10.1186/s12870-024-05190-6)
Supplement: Supplementary file 5 — Supplementary Material 5. [file 12870_2024_5190_MOESM5_ESM.pdf]

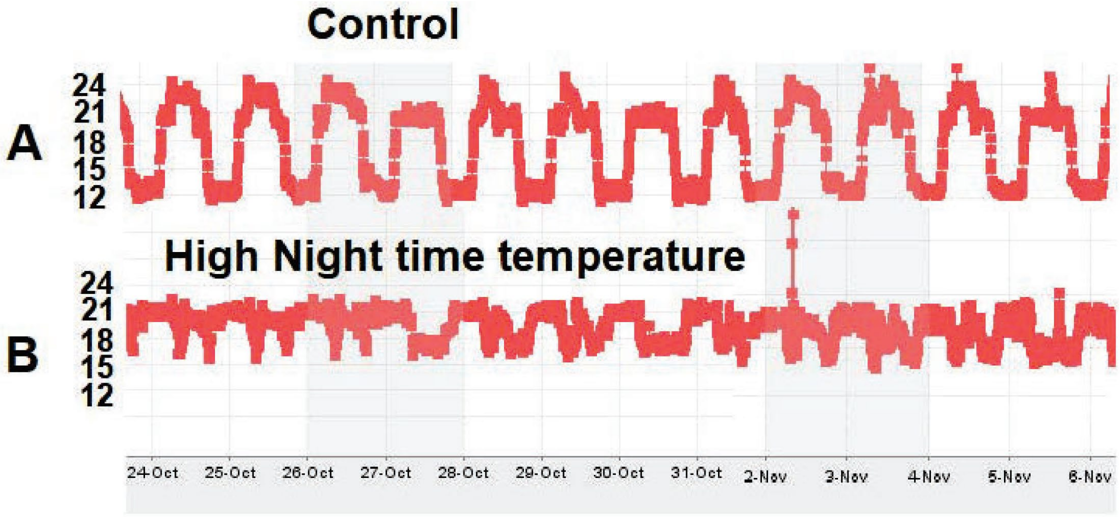

Supplemental Fig. S5. Greenhouse temperatures under control and high night temperature conditions. X axis shows day of measurement, and Y axis shows temperature in degrees Celsius. (a) Control greenhouse temperatures. (b) High night temperature greenhouse temperatures.
