# Supplementary material for: Tissue- and time-dependent metabolite profiles during early grain development under normal and high night-time temperature conditions
Source: BMC Plant Biol. 2024 Jun 18;24:568. doi: 10.1186/s12870-024-05190-6 (PMC11184705; doi:10.1186/s12870-024-05190-6)
Supplement: Supplementary file 6 — Supplementary Material 6. [file 12870_2024_5190_MOESM6_ESM.pdf]

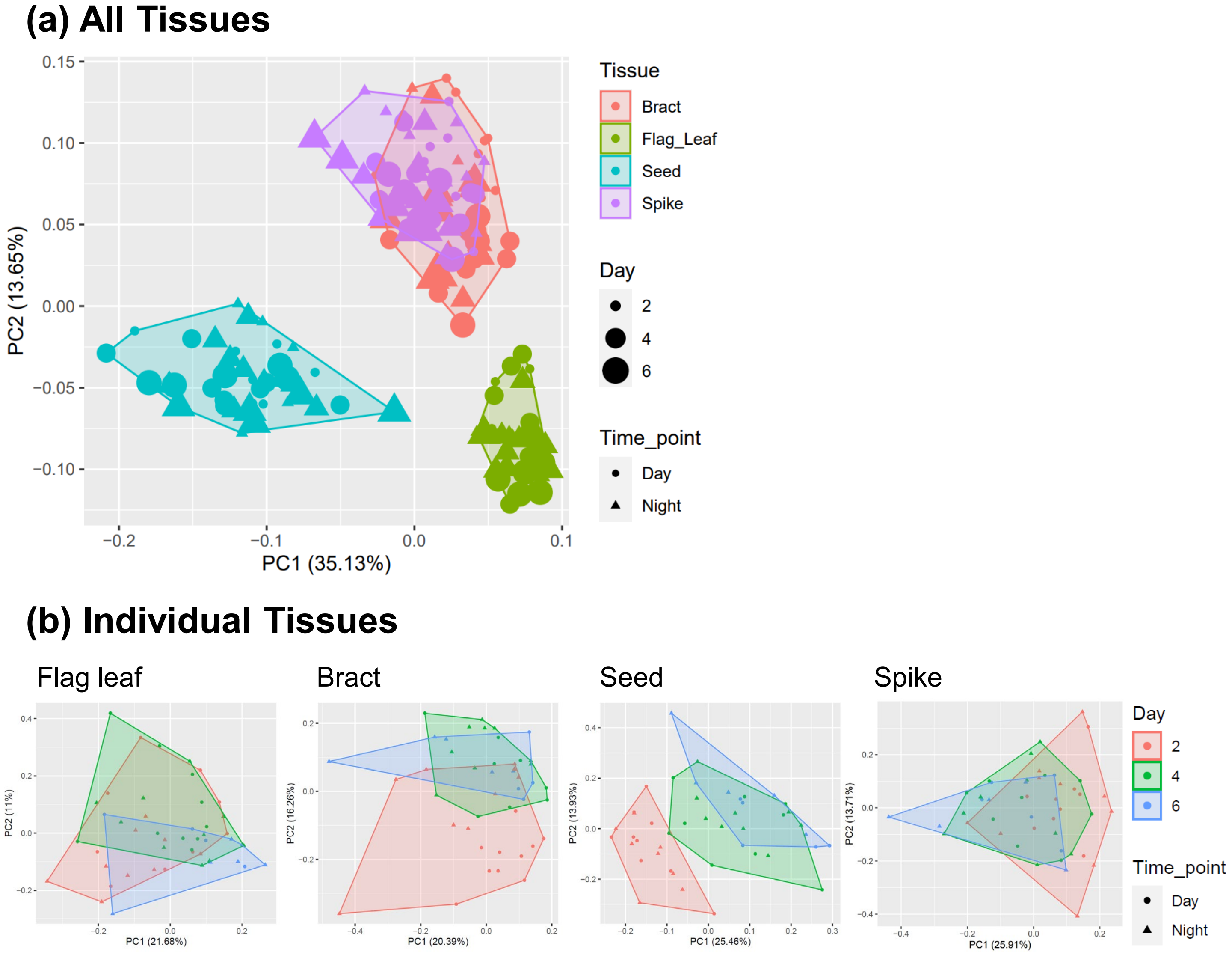

Supplemental Fig. S1. Global changes in metabolite profile depending on developmental stages and day/night. The score plots show the results of the principle component analysis (PCA) of global metabolite profiles. The shade indicate the minimum area include all data points in the category. (a) PCA of the metabolite profiles of all tissues. The color indicate the tissues (red, bract; green, flag leaf; blue, seed; purple, spike), size of the point indicates DAF (small, 2 DAF; medium, 4 DAF; large, 6 DAF), and the shape of the point indicates the time point in the day (circle, day; triangle, night). (b) PCA of the metabolite profiles in individual tissues. The color indicates DAF (red, 2 DAF; green, 4 DAF; blue, 6 DAF), and the shape of the point indicates the time point in the day (circle, day; triangle, night). The panels from the left; flag leaf, bract, seed, and spike.
